# Factors Affecting the Treatment Heterogeneity of PPARγ and Pan-PPAR Agonists in Type 2 Diabetes Mellitus: A Systematic Review and Machine Learning-Based Meta-Regression Analysis

**DOI:** 10.3390/ph19010139

**Published:** 2026-01-13

**Authors:** Xinlei Zhang, Yingning Liu, Ming Chu, Linong Ji, Xiantong Zou

**Affiliations:** 1 Beijing Key Laboratory of Innovative Drug and Device Translation in Endocrine and Metabolic Diseases, Department of Endocrinology and Metabolism, Peking University People’s Hospital, Beijing 100044, China; zhxl@stu.pku.edu.cn (X.Z.); yingningliu@pku.edu.cn (Y.L.); 2 Peking University Diabetes Center, Peking University Health Science Center, Beijing 100191, China; 3 NHC Key Laboratory of Medical Immunology, Department of Immunology, School of Basic Medical Sciences, Peking University, Beijing 100191, China; famous@bjmu.edu.cn

**Keywords:** peroxisome proliferator-activated receptor γ agonists, pan peroxisome proliferator-activated receptor γ agonists, type 2 diabetes mellitus, meta-analysis, glycaemic lowering effect

## Abstract

**Background/Objectives**: Significant heterogeneity in the treatment response to peroxisome proliferator-activated receptor γ (PPARγ) agonists exists, and predictive factors for their efficacy remain unclear. We aimed to assess the relationships between routinely available clinical features and the efficacy of PPARγ agonists and pan-PPAR agonists by meta-regression analysis. **Methods**: We searched PubMed, Embase, Cochrane Library, ClinicalTrials.gov and the WHO International Clinical Trials Registry Platform (ICTRP) and included randomised controlled trials involving type 2 diabetes patients with 12-week or longer treatment durations with PPARγ agonists or pan-PPAR agonists published before 11 November 2023 (PROSPERO registration number: CRD42024578987). We conducted mixed-effect meta-regression analyses between baseline variables and treatment response. Moreover, we developed a machine learning-based meta-forest model and ranked the relative importance of each variable. **Results**: In 147 studies involving 29,250 participants, PPARγ and pan-PPAR agonists significantly reduced HbA1c (mean difference(MD) = −0.8876 [95% confidence interval (CI): −0.8999, −0.8754]; *p* < 0.0001, *I*^2^ = 96.0%) and FPG = (MD = −1.7900 [95% CI: −1.9137, −1.6663]; *p* < 0.0001, *I*^2^ = 92.0%). Multivariable association analysis suggested that a greater proportion of female participants (*β* = 0.0066 [95% CI: 0.0012, 0.0121]; *p* = 0.017), younger age (*β* = −0.0314 [95% CI: −0.05, −0.0129]; *p* = 0.0009) and lower HDL-C levels (*β* = −0.9304 [95% CI: −1.5176, −0.3431]; *p* = 0.0019) were significantly associated with a greater decrease in HbA1c. A greater proportion of female participants (*β* = 0.0112 [95% CI: 0.0019, 0.0205]; *p* = 0.0178) and lower baseline HDL-C levels (*β* = −1.8722 [95% CI: −2.812, −0.9323]; *p* < 0.0001) were significantly associated with a greater decrease in FPG. These variables also ranked among the top five most important predictors of drug response in the meta-random forest models. **Conclusions**: Our study demonstrated that female sex, younger age, and lower HDL-C levels were associated with greater glycaemic lowering effect from PPARγ and pan-PPAR agonists.

## 1. Introduction

Type 2 diabetes mellitus (T2DM) is a heterogeneous chronic disease that exhibits significant variability in clinical features, pathophysiology, and disease progression. A key manifestation of this complexity is the variable treatment response to glucose-lowering medications, which is known as the heterogeneity of the treatment effect (HTE). The identification of routinely available clinical characteristics that contribute to HTE can facilitate patient-specific therapy selection and improve diabetes management. Both traditional subgroup analyses and machine learning methods have demonstrated features that are routinely collected in the clinical setting as being predictors of better glucose-lowering responses to various medications, including DPP4 inhibitors, SGLT2 inhibitors and GLP1 receptor agonists [1,2,3,4]. However, most studies have originated from individual clinical trials or databases, and the generalisability of their conclusions is restricted. A broader population-level understanding of HTE and the associated clinical characteristics is needed to identify more generalisable and robust predictors of drug efficacy.

PPARγ (peroxisome proliferator-activated receptor γ) agonists, including pioglitazone and rosiglitazone, have been used for over three decades in T2DM treatment. Despite controversies surrounding their side effects such as oedema, weight gain, and heart failure, these drugs offer distinct glucose-lowering benefits [5]. The recent development of pan-PPAR agonists like chiglitazar, which simultaneously activate multiple isoforms of PPAR (PPARα, PPARγ, and PPARβ/δ), is broadening the therapeutic potential beyond that of selective PPARγ agonists [6,7]. However, individual responses to these drugs are heterogeneous, and the factors influencing their efficacy are not fully understood. Previous machine learning analyses of CMAS and CMAP trials in Chinese T2DM patients identified drug responders to chiglitazar, with these responders more likely to be female and characterised by high BMI levels, low HDL-C levels, and high insulin resistance [8]. However, this result may be limited to only chiglitazar; moreover, the factors affecting the treatment effect of PPARγ agonists (especially across a variety of trials and cohorts) are still unknown. To more comprehensively assess routinely available clinical characteristics associated with the efficacy of PPARγ agonists and pan-PPAR agonists, we utilised data from published randomised controlled trials (RCTs) and performed a systematic review and meta-regression analysis.

## 2. Materials and Methods

The systematic review and meta-analysis were performed and reported following the Preferred Reporting Items for Systematic Reviews and Meta-Analyses (PRISMA) statements. The protocol was registered in The International Prospective Register of Systematic Reviews (PROSPERO) as CRD42024578987.

### 2.1. Data Sources and Searches

A comprehensive literature search was conducted in PubMed, Embase, the Cochrane Library, ClinicalTrials.gov and the WHO International Clinical Trials Registry Platform (ICTRP) from inception to 11 November 2023. The reference lists of the retrieved publications and relevant systematic reviews were also screened for potential eligible studies. The applied search terms for each database and the full search strategy are listed in the Supplementary Materials Section S1.

### 2.2. Study Selection

Eligible randomised controlled trials were required to have enrolled adults with type 2 diabetes who received PPARγ agonists or pan-PPAR agonists with a duration of intervention of at least 12 weeks and evaluated outcomes in terms of changes in HbA1c or fasting plasma glucose (FPG). Treatment was administered either as monotherapy or in combination with nonrandomised stable background glucose-lowering therapy. The comparator could be a placebo or any hypoglycaemic active drug. We excluded trials with a crossover design; trials enrolling patients with prediabetes or gestational diabetes; trials specifically recruiting patients with haematological disorders, malignancies or haemodialysis; trials where the background antidiabetic therapy could be altered. We did not exclude trials that used drugs that were withdrawn or not used in clinical practice.

After deduplication via Zotero 6.0 (Corporation for Digital Scholarship, Vienna, VA, USA), two reviewers (X.Zh. and Y.L.) independently screened the titles and abstracts of the retrieved records and examined the full texts of potentially eligible records. Discrepancies were resolved via discussions within the review team.

### 2.3. Data Extraction and Quality Assessment

For each eligible study, we extracted various data using a unified form. When available, we also extracted stratified outcome data according to different baseline characteristics of patients within each study.

For the primary and secondary outcome data, the mean difference between preintervention and postintervention values and their standard errors (SE) were extracted as the summary measure of the treatment effect. If the standard error data were missing and could not be alternatively calculated, these data were imputed with the minimum correlation coefficient between preintervention and postintervention values estimated from studies with similar populations [9]. For HbA1c, mean values were converted to % using the formula 0.09148 × mmol/mol + 2.152, while SE values were converted by multiplying by 0.09148. For fasting plasma glucose, both mean and SE values were converted from mg/dL to mmol/L by dividing by 18. For multi-arm studies using varying dosages of the same drug, we merged these treatment groups according to the formula in Chapter 6.5 of the Cochrane Handbook to avoid units of analysis errors [10]. The risk of bias for each study was evaluated with the Risk of Bias 2 tool (RoB 2).

### 2.4. Data Synthesis and Analysis

We first conducted an inverse variance random-effects meta-analysis. The restricted maximum likelihood estimator and Knapp–Hartung adjustment were applied in the model. We quantified the between-study heterogeneity via a Cochran’s Q test and the I^2^ statistic. The potential risk of publication bias was assessed by constructing funnel plots, and asymmetry was assessed via the Egger’s test and Begg–Mazumdar’s test.

To identify the clinical characteristics contributing to treatment heterogeneity, we conducted meta-regression analyses. For categorical variables, we employed a mixed-effects subgroup analysis. For continuous baseline characteristics, we performed univariable meta-regression analyses (mixed-effect model) including age, female proportion, diabetes duration, body mass index (BMI), homeostatic model assessment of insulin resistance (HOMA-IR), fasting insulin, high-density lipoprotein cholesterol (HDL-C), low-density lipoprotein cholesterol (LDL-C), and baseline levels of HbA1c and FPG. We separately performed subgroup meta-regression analyses for trials in which glitazones (PPARγ agonists) and glitazars (dual and pan-PPAR agonists) were used as treatments. We also performed a meta-regression adjusting for baseline HbA1c/FPG levels by incorporating these factors as covariates in the regression. Variables demonstrating statistical significance or that were considered to be clinically significant were subsequently incorporated into an exploratory multivariable meta-regression model. We excluded regression analyses with fewer than 10 studies [11]. *p* values were adjusted for multiple comparisons via the Benjamini–Hochberg method.

To further identify the sources of treatment heterogeneity, we employed metaforest, which is a machine learning-based approach that optimises a random forest algorithm for meta-analysis [12,13]. A metaforest model was subsequently generated, consisting of 10,000 regression trees, with four candidate variables per split and a minimum of three cases per terminal node. The relative importance of each variable was quantified via the (shapley additive explanation(SHAP) value [14].

To evaluate the robustness of our findings, we conducted several sensitivity analyses of the meta-regression. These analyses included (1) the exclusion of studies with imputed standard deviations; (2) the exclusion of studies exhibiting a high risk of bias; (3) subgroup analyses of studies using the drug as add-on therapy or monotherapy; (4) the exclusion of studies specifically enrolling patients with comorbidities (such as coronary artery disease and non-alcoholic fatty liver disease); and (5) the exclusion of studies with small sample sizes (fewer than 15 patients per treatment arm). Furthermore, we separately analysed placebo-controlled studies to assess the influence of the placebo response on treatment effects.

The statistical analyses were performed with R version 4.3.1 software (R Core Team, Vienna, Austria) with the R packages meta (version 7.0.0), metafor (version 4.6.0) and metaforest (version 0.1.4).

## 3. Results

### 3.1. Search Results

**Figure 1:**
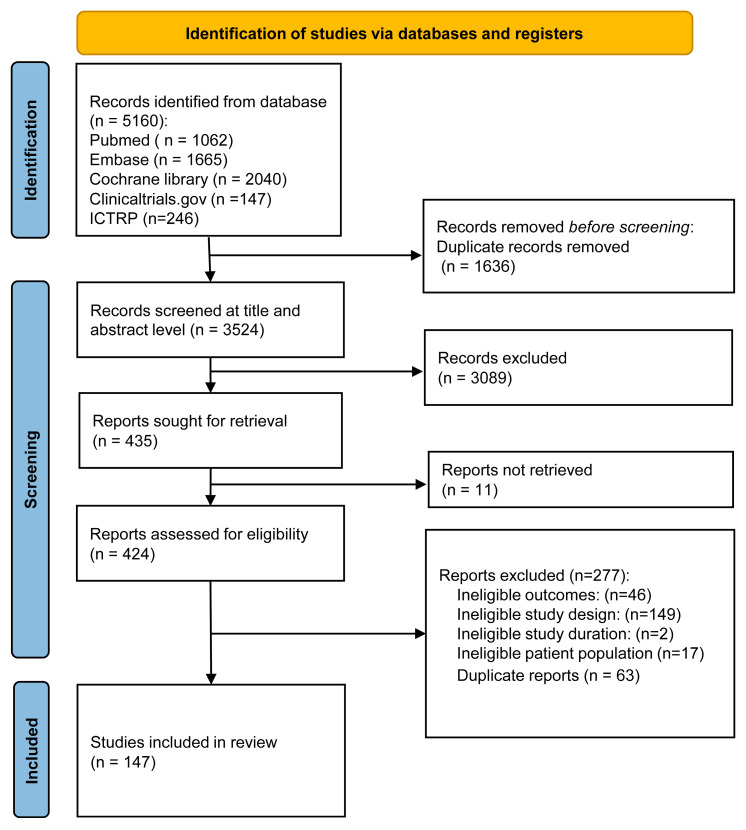
Flowchart of study selection for the meta-analysis.

A total of 5160 citations were retrieved from the search. After deduplication and screening for titles and abstracts, 424 reports were included for full-text screening. After excluding 277 reports that did not meet the eligibility criteria, 147 studies were included in the final analysis. The results of the search and screening are summarised in Figure 1.

### 3.2. Study Characteristics

The included studies spanned the years 1995–2022 and collectively enrolled 29,250 individuals with type 2 diabetes. 139 Articles were published in English, 6 in Chinese, and 2 in Korean. The median number of participants in each study was 83 (interquartile range (IQR): 32–174). The following PPARγ agonists or pan-PPAR agonists were employed as treatment interventions in the studies: pioglitazone (n = 91), rosiglitazone (n = 40), troglitazone (n = 7), lobeglitazone (n = 4), rivoglitazone (n = 3), balaglitazone (n = 1), tesaglitazar (n = 5), muraglitazar (n = 6), aleglitazar (n = 3), chiglitazar (n = 2), and ragaglitazar (n = 1). Treatment interventions were classified as monotherapy in 83 trials and as add-on therapy in 64 trials. Furthermore, 56 trials were placebo-controlled, and 91 trials were active-controlled. A summary of characteristics of included studies is shown in Supplementary Table S1.

We assessed the risk of bias using the RoB 2 tool. For both HbA1c and FPG outcomes, the majority of domains showed a low risk of bias, particularly in outcome measurement and reporting. Some concerns were noted regarding the randomization process, deviation from intended interventions, and missing outcome data in a minority of studies. (Supplementary Figure S1, Tables S3 and S4). The funnel plots for the endpoints of both HbA1c reduction and FPG reduction exhibited symmetric distributions (Supplementary Figures S2 and S3). The results of Egger’s test and Begg–Mazumdar’s test for changes in HbA1c (*p* = 0.6035 and *p* = 0.1196, respectively) and FPG (*p* = 0.3143 and *p* = 0.0909, respectively) were nonsignificant, thus indicating the absence of publication bias.

### 3.3. Treatment Effects of PPARγ Agonists and Pan-PPAR Agonists

**Figure 2:**
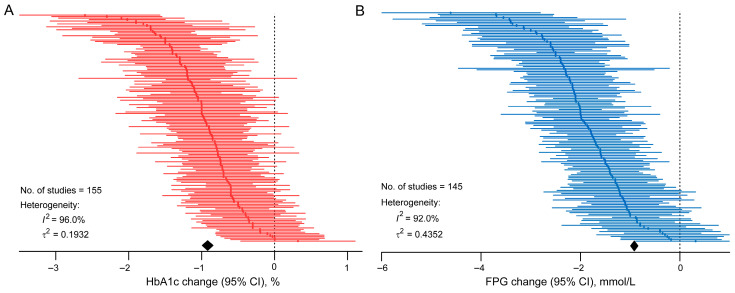
Caterpillar plot for the change in HbA1c and FPG after the treatment of PPARγ agonists or pan-PPAR agonists. Effect sizes (mean difference with 95% confidence intervals) of each study were depicted as individual dots and lines. The pooled effect size was summarized by the diamond shape below each plot, calculated using a random-effects model. (**A**) Changes in HbA1c; (**B**) Changes in FPG. FPG: fasting plasma glucose.

Meta-analysis revealed a significant reduction in HbA1c (mean difference (MD) = −0.8876 [95% CI: −0.8999, −0.8754]; *p* < 0.0001) and FPG (MD = −1.7900 [95% CI: −1.9137, −1.6663]; *p* < 0.0001) after treatment with PPARγ agonists and pan-PPAR agonists (Figure 2, Supplementary Figures S4 and S5). Considerable heterogeneity between the studies existed for both HbA1c and FPG endpoints, with *p* values of the Cochran Q test being lower than 0.0001 and Higgins’s *I*^2^ statistics of 96.0% and 92.0%, respectively.

### 3.4. Factors Associated with Treatment Response

#### 3.4.1. Univariable Association Analysis

**Figure 3:**
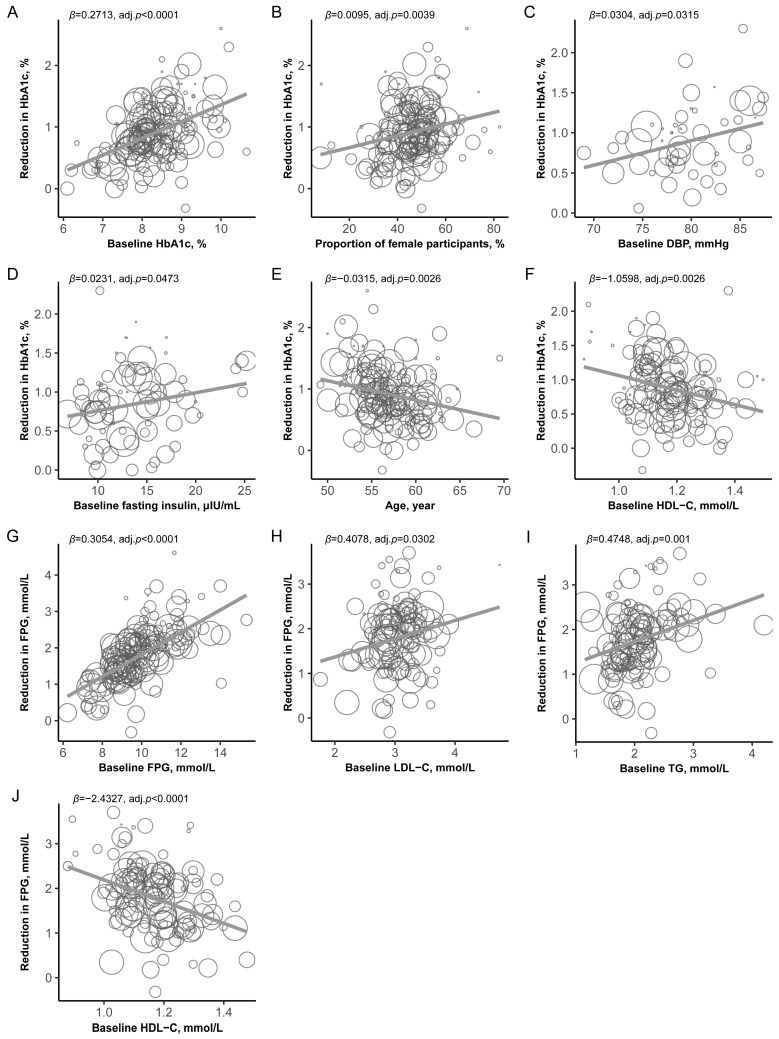
Factors associated with HbA1c decline and FPG decline. Association between HbA1c decline and baseline HbA1c (**A**), female proportion (**B**), baseline DBP (**C**), baseline fasting insulin (**D**), age (**E**) and baseline HDL-C (**F**). Association between FPG decline and baseline FPG (**G**), baseline LDL-C (**H**), baseline TG (**I**) and baseline HDL-C (**J**). The circles represent individual studies. The size of each circle represents the weight (inverse variance) assigned to the study. The solid grey line represents the fitted meta-regression slope. FPG: fasting plasma glucose; HDL-C: high-density lipoprotein cholesterol; LDL-C: low-density lipoprotein cholesterol; TG: triglyceride. *p* values were adjusted by the Benjamini–Hochberg method.

Univariable meta-regression analyses revealed several factors that were significantly associated with treatment response and accounted for high treatment heterogeneity (adjusted *p* < 0.05; Supplementary Table S6, Figure 3). After correcting for multiple tests, higher baseline HbA1c levels (*β* = 0.2713 [95% CI: 0.2005, 0.3421]; adj.*p* < 0.0001) (Figure 3A), a greater proportion of female participants (*β* = 0.0095 [95% CI: 0.0038, 0.0152]; adj.*p* = 0.0039) (Figure 3B), elevated baseline diastolic blood pressure (*β* = 0.0304 [95% CI: 0.0061, 0.0547]; adj.*p* = 0.0315) (Figure 3C), and higher fasting insulin levels (*β* = 0.0231, [95% CI: 0.0028, 0.0435]; adj.*p* = 0.0473) (Figure 3D) demonstrated significant positive correlations with the magnitude of HbA1c reduction. Conversely, both age (*β* = −0.0315 [95% CI: −0.0497, −0.0133]; adj.*p* = 0.0026) (Figure 3E) and baseline HDL-C levels (*β* = −1.0598 [95% CI: −1.6706, −0.449]; adj.*p* = 0.0026) (Figure 3F) were significantly negatively correlated with the decline in HbA1c. After adjusting for baseline HbA1c levels, most of the associations remained significant, with the exceptions of baseline DBP (*β* = 0.0251, [95% CI: 0.0022, 0.048]; adj.*p* = 0.0529); moreover, baseline triglyceride levels (*β* = −0.1881, [95% CI: −0.331, −0.0452]; adj.*p* = 0.0329) were negatively associated with the decline in HbA1c (Table 1).

After applying corrections for multiple testing, higher baseline levels of FPG (*β* = 0.3054 [95% CI: 0.2571, 0.3537]; adj.*p* < 0.0001) (Figure 3G), LDL-C(*β* = 0.4078, [95% CI: 0.0935, 0.722]; adj.*p* = 0.0302) (Figure 3H), and TGs (*β* = 0.4748, [95% CI: 0.2193, 0.7303]; adj.*p* = 0.001) (Figure 3I), as well as lower baseline HDL-C levels (*β* = −2.4327, [95% CI: −3.45, −1.4154]; adj.*p* < 0.0001) (Figure 3J), were significantly associated with a greater reduction in FPG. A greater proportion of female participants and higher baseline HOMA-IR index was initially associated with a greater decline in FPG; however, these associations became nonsignificant after multiple testing correction (*β* = 0.0105, [95% CI: 0.0011, 0.0198], *p* = 0.0279, adj.*p* = 0.06; *β* = 0.0816, [95% CI: 0.0067, 0.1565], *p* = 0.0327, adj.*p* = 0.06, respectively) (Supplementary Table S5). After adjusting for baseline FPG levels, no associations remained significant with the exceptions of baseline HDL-C (*β* = −1.1985, [95% CI: −1.991, −0.406]; adj.*p* = 0.0304) (Table 1).

In the PPARγ mono-agonist(glitazones) subgroup, the significant associations observed in the unadjusted univariable meta-regression analyses remained consistent (Supplementary Figure S6). In the pan-PPAR agonist or dual-PPAR agonist (glitazars) subgroup, only younger age and higher baseline fasting insulin demonstrated robust correlations with HbA1c reduction. (Supplementary Figure S6). These findings remained consistent when baseline HbA1c or FPG adjustments were incorporated into the meta-regression models (Supplementary Tables S7 and S8). In drug-specific analyses, only lower baseline TG levels were significantly associated with HbA1c decline in patients treated with pioglitazone (*β* = −0.365, [95% CI: −0.5508, −0.1781], adj.*p* = 0.001) (Supplementary Table S9). For rosiglitazone, although a higher proportion of female participants and baseline fasting insulin were initially associated with HbA1c decline, these correlations became non-significant after multiple testing correction (*β* = 0.0113, [95% CI: 0.0029, 0.0197], *p* = 0.0082, adj.*p* = 0.082; *β* = 0.0293, [95% CI: 0.0004, 0.0581], *p* = 0.0466, adj.*p* = 0.191, respectively) (Supplementary Table S10).

#### 3.4.2. Multivariable Association Analysis

Based on the previous results, several factors were deemed to be significant and were included in the multivariable meta-regression model, including baseline HbA1c/FPG; proportion of female participants; age; and baseline levels of HDL-C, BMI and TGs. Furthermore, to mitigate potential confounding from trial design, the model also incorporated ‘Background therapy’ (monotherapy vs. add-on therapy) and ‘Drug type’ (glitazones vs. glitazars). ‘Background therapy’ was defined by the treatment strategy (PPAR agonist used alone or with other glucose-lowering drugs), while ‘Drug type’ was categorized by the drug’s mechanism of action (PPARγ agonists vs. pan-PPAR agonists). Multicollinearity was checked by variance inflation factors, with all values being < 2.0 (Supplementary Table S11). We found that a higher baseline HbA1c (*β* = 0.1881, [95% CI: 0.1051, 0.2711], *p* < 0.0001), higher proportion of female participants (*β* = 0.0066, [95% CI: 0.0012, 0.0121], *p* = 0.017), younger age (*β* = −0.0314, [95% CI: −0.05, −0.0129], *p* = 0.0009) and lower HDL-C levels (*β* = −0.9304, [95% CI: −1.5176, −0.3431], *p* = 0.0019) were significantly associated with a more pronounced decline in HbA1c. Similarly, higher baseline FPG levels (*β* = 0.2799, [95% CI: 0.2099, 0.3499]; *p* < 0.0001), a greater proportion of female participants (*β* = 0.0112 [95% CI: 0.0019, 0.0205]; *p* = 0.0178) and lower baseline HDL-C levels (*β* = −1.8722 [95% CI: −2.812, −0.9323]; *p* < 0.0001) were significantly associated with a greater decline in FPG (Table 2).

#### 3.4.3. Predictors Identified via the Metaforest Model

**Figure 4:**
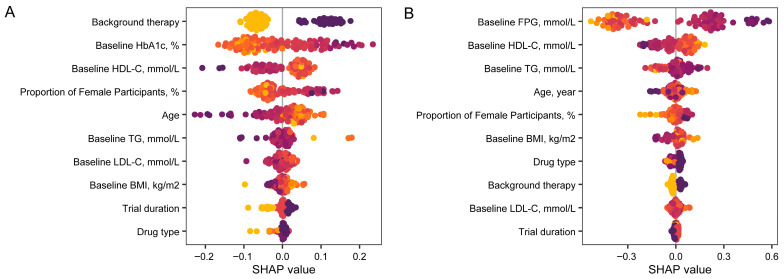
Relative importance of variables in predicting treatment response to PPARγ agonists and pan-PPAR agonists using a meta-random forest model. A meta-random forest model was developed to predict the decline in HbA1c or FPG following treatment. Shapley additive explanations (SHAP) summary plot showing the importance of each variable on the prediction of HbA1c reduction (**A**) or FPG reduction (**B**) with treatment of PPARγ agonists and pan-PPAR agonists. The y-axis represents predictor variables ranked in descending order of importance, while the x-axis indicates the impact of each variable on the model output. Larger SHAP values indicate a stronger positive impact on the model output. Each point represents a single clinical trial, with color gradients reflecting the corresponding value of each variable. FPG: fasting plasma glucose; BMI: body mass index; FPG: fasting plasma glucose; LDL-C: low-density lipoprotein cholesterol; HDL-C: high-density lipoprotein cholesterol; TG: triglyceride.

We incorporated baseline variables from the multivariable regression analysis to construct metaforest models for reductions in HbA1c and FPG levels. The R_oob_^2^ values for these aforementioned models were positive (0.2763 and 0.4198, respectively), thereby indicating that the fitted models demonstrated good explanatory power for the heterogeneity of the treatment response. Using SHAP values to rank the relative importance of each variable, we observed that background therapy, higher baseline HbA1c levels and lower baseline HDL-C levels exhibited the greatest influence on HbA1c decline, followed by a greater proportion of females and younger age. Higher baseline FPG levels, lower HDL-C levels, higher TG levels, younger age and a greater proportion of females were the five key factors impacting FPG decline, which are ranked in descending order of importance (Figure 4). Finally, partial dependence plots elucidated the potentially non-linear associations between these baseline characteristics and the changes in HbA1c and FPG levels (Supplementary Figure S7 and Table S8).

### 3.5. Sensitivity Analysis

The results of the univariable meta-regression analysis remained robust based on the sensitivity analysis, which involved excluding studies with a high risk of bias (Supplementary Table S12), studies with small sample sizes (Supplementary Table S13), studies with imputed standard deviations (SD) (Supplementary Table S14) and studies enrolling patients with specific complications (Supplementary Table S15). In the add-on therapy subgroup, higher baseline HOMA-IR was also significantly correlated with a reduction in HbA1c levels, whereas the correlations of female proportion and baseline TG with treatment response were no longer significant (Supplementary Table S17). Interestingly, when accounting for the placebo response in the meta-regression analysis, no factors were observed to be associated with treatment response (Supplementary Table S19). Moreover, we did not identify any factors associated with the placebo response (Supplementary Table S20).

In a multivariable meta-regression that additionally incorporated DBP into the regression model, the overall correlations remained consistent except for baseline age. Moreover, the positive association between baseline DBP and HbA1c decline achieved borderline significance (*β* = 0.027 [95% CI: 0.0, 0.054]; *p* = 0.05) (Supplementary Table S21). Consistently, no significant correlation between BMI and treatment response was identified.

## 4. Discussion

In this meta-analysis of 139 studies comprising 28,534 T2D patients, we demonstrated a significant reduction in both HbA1c and FPG levels following PPARγ agonist and pan-PPAR agonist therapy and revealed that female sex, younger age, and lower HDL levels were significantly associated with greater glucose-lowering effects of PPARγ agonists and pan-PPAR agonists.

Our analysis revealed that the proportion of female participants is a significant factor influencing the treatment response to PPARγ agonists and pan-PPAR agonists. Both the univariable and multivariable meta-regression analyses revealed that a greater proportion of females was associated with a greater decline in HbA1c, thus suggesting increased efficacy of PPARγ agonists and pan-PPAR agonists in women compared to men. This finding aligns with previous studies demonstrating stronger glycaemic effects of pioglitazone and rosiglitazone in female subgroups. Responder groups for rosiglitazone and ciglitazone also exhibited a higher proportion of females compared to nonresponders [8,15,16,17]. These observations may be attributed to several factors. First, women typically possess a greater total amount of subcutaneous adipose tissue, which is the primary site of PPARγ agonist action [15]. Moreover, oestrogen promotes PPARγ expression and activity in adipose tissue, whereas androgens lack the same effect [18]. Additionally, the liver enzyme CYP2C8, which metabolises PPARγ agonists, is expressed at lower levels in women, thus resulting in slower clearance of the drug and prolonged activity [19]. These mechanisms may collectively account for the sex differences observed in the efficacy of PPARγ agonists and pan-PPAR agonists.

Both univariable and multivariable meta-regression analyses indicated a significant negative correlation between age and HbA1c reduction, thereby suggesting that younger patients may experience greater therapeutic efficacy with PPARγ agonists and pan-PPAR agonists. This finding differed from previous findings demonstrating equivalent efficacy in elderly patients (age > 65/60 years) compared with younger patients (age < 65/60 years) [20,21,22]. However, despite this observed efficacy, the use of PPARγ agonists in elderly populations is often limited, which is primarily due to concerns about oedema, increased risk of heart failure, and increased fracture risk. Therefore, in conjunction with our findings, PPARγ agonists may be a suboptimal treatment option for elderly patients [23].

Our analysis did not identify baseline BMI as a robust predictor of the efficacy of PPARγ agonists and pan-PPAR agonists. Although several subgroup analyses have demonstrated superior glycaemic benefits of PPARγ agonists in high-BMI versus low-BMI groups, other investigations have indicated that the waist-to-hip ratio (WHR) affects PPARγ agonist efficacy rather than BMI [15,24]. This result suggests that central obesity is associated with better treatment outcomes rather than extreme general obesity [16]. One study also revealed that mildly obese patients derived greater glycaemic benefits from troglitazone than did severely obese patients [25]. These findings may suggest a potential nonlinear relationship between baseline BMI and PPARγ agonist efficacy. However, few studies have reported results regarding waist circumference or the WHR; therefore, we were unable to investigate the effects of central obesity on the efficacy of PPARγ agonists and pan-PPAR agonists.

Surprisingly, we observed that baseline DBP levels were significantly positively correlated with reductions in HbA1c in both the univariable and multivariable regression analyses; however, this significance was lost after adjusting for multiple tests in the univariable regression analysis. These findings, while preliminary and requiring caution, indicate that baseline DBP is a potential predictor of efficacy of PPARγ agonist and pan-PPAR agonist. The influence of baseline blood pressure on the efficacy of PPARγ agonists and pan-PPAR agonists has rarely been reported in previous studies. A randomised controlled trial evaluating rosiglitazone revealed that responders demonstrated significantly greater baseline systolic blood pressure compared to nonresponders, which aligns with our findings [16]. In hypertensive patients, the activation of the RAAS system is often accompanied by the upregulation of the Wnt/β-catenin pathway, which further reduces PPARγ expression and inhibits its pathway activity. These changes may partially explain the increased efficacy of PPARγ agonists and pan-PPAR agonists observed in hypertensive populations [26,27].

Our study consistently revealed that lower baseline HDL-C levels were associated with greater glucose-lowering efficacy of PPARγ agonist and pan-PPAR agonist treatment across multiple adjustments and sensitivity analyses. This finding aligns with previous reports showing lower HDL-C among pioglitazone responders compared with non-responders [28]. In contrast, the association of baseline triglycerides (TGs) with treatment response was more complex. In the univariable regression adjusted for baseline HbA1c levels, lower baseline TG levels were significantly associated with a greater reduction in HbA1c, although this association was weakened in the multivariable analysis. Conversely, higher TGs were associated with a greater reduction in FPG, but this relationship disappeared after adjusting for baseline FPG, possibly suggesting regression-to-the-mean effects. These divergent patterns indicate that HDL-C and TGs are not interchangeable predictors. Mechanistically, high TGs reflect severe insulin resistance and increased circulating lipids, which may initially amplify FPG reduction with therapy. However, persistent postprandial defects and possible PPARγ receptor desensitization from chronic exposure to endogenous fatty acid ligands could limit overall HbA1c improvement [29]. In contrast, low HDL-C not only reflects insulin resistance but also chronic inflammation, oxidative stress, and endothelial dysfunction [30]. A cornerstone of PPARγ agonist action, distinct from its metabolic effects, is its potent anti-inflammatory activity. PPARγ activation can inhibit the activity of key pro-inflammatory transcription factors, including NF-κB and AP-1 [29]. Therefore, in patients with low baseline HDL-C, PPARγ agonists have a significant “inflammatory burden” to correct, resulting in a more pronounced improvement in glycaemic control. Taken together, these findings suggest that baseline dyslipidaemia, particularly low HDL-C, may enhance the glycaemic efficacy of PPARγ agonists and pan-PPAR agonists, underscoring the interplay between lipid metabolism, inflammation, and glucose regulation.

Our findings were further supported by machine learning-based analysis, which indicated that a combination of features could predict the treatment response to PPARγ agonists and pan-PPAR agonists. Age, female sex, and lower baseline HDL levels were associated with reductions in both HbA1c and FPG, whereas a greater reduction in FPG was additionally associated with higher baseline TG levels. Similarly, in our previous study, machine learning identified low HDL-C and high TG levels as key predictors of the chiglitazar response [8]. In contrast to traditional one-variable-at-a-time regression analyses, the random forest model provides a quantitative, cross-validated measure of moderator importance. Furthermore, this method effectively mitigates issues such as overfitting and multicollinearity, and it also accounts for potential interactions between variables.

The main findings of this study were consistent between the primary and sensitivity analyses. Notably, no factors associated with a reduction in HbA1c were identified within the pan-PPAR (glitazars) subgroup. This may be explained by the subgroup’s intrinsic heterogeneity regarding PPARγ affinity. Crucially, when analysing pioglitazone and rosiglitazone separately, the robust associations observed in the combined analysis were attenuated. This discrepancy suggests that while these factors represent shared biological drivers of response across the PPARγ agonist class, they are less consistent when isolated to specific drugs, partially due to reduced sample sizes. Similarly, adjusting for the placebo response negated previous associations. This is plausibly attributed to compromised statistical power and potential selection bias, as the number of eligible studies dropped significantly from 147 to 59 in this adjustment.

Our meta-regression analysis identified several characteristics, such as female sex, younger age, and lower HDL-C levels, that may influence the therapeutic effects of PPAR agonists. The clinical significance lies in its potential to inform a more stratified approach to treatment, generating hypotheses for precision medicine in this area. However, physicians should be aware that although these factors represent biologically plausible predictors rooted in the shared PPARγ mechanism, the robustness of their predictive value may vary by specific drug. Furthermore, the clinical application of these predictors must be carefully balanced against the established safety profile of PPARγ agonists. Well-documented adverse effects such as fluid retention, weight gain, and potential cardiovascular risks associated with specific agents remain significant clinical barriers [5]. Specifically, the observed efficacy benefits in women must be weighed against their heightened susceptibility to PPARγ-induced bone mineral density loss and fractures [31].

This study has several limitations. Firstly, our analysis relied exclusively on between-study comparisons rather than within-study comparisons, thereby potentially reducing the statistical power to detect significant differences and increasing susceptibility to confounding factors [32]. Furthermore, our findings are susceptible to ecological bias, as differences in “average” treatment responses across populations may not reflect genuine variations in the clinical characteristics of subject groups; rather, these differences may arise due to heterogeneity in trial protocols, study environments, measurement methodologies, and other confounding variables [33,34]. Thirdly, not all studies were eligible for inclusion in the regression analysis due to incomplete data reporting, which potentially introduces selection bias. Fourthly, our study cannot establish cut-off values for continuous variables to guide optimal anti-diabetic drug selection. Finally, our analysis was restricted to glycaemic efficacy. We did not investigate factors modifying critical adverse events or long-term clinical outcomes. In summary, the findings from our study should be interpreted with caution and warrant further validation with individual-level data.

## 5. Conclusions

The meta -analysis demonstrates significant heterogeneity in the therapeutic efficacy of PPARγ agonists and pan-PPAR agonists. Specifically, female sex, younger age and low HDL-C levels were consistently associated with greater glycaemic lowering benefits. While these observations offer insights into personalized approaches for type 2 diabetes mellitus, their clinical implication requires a careful evaluation of the safety profiles associated with the drugs. Further validation is needed to confirm their clinical relevance.

## Figures and Tables

**Table 1:** Univariable meta-regression analysis assessing the association between treatment response and baseline factors * represents adjustment for baseline HbA1c; ^‡^ represents adjustment for baseline FPG. *p* values were adjusted by the Benjamini–Hochberg method. BMI: body mass index; FPG: fasting plasma glucose; SBP: systolic blood pressure; DBP: diastolic blood pressure; HOMA-IR: homeostatic model assessment of insulin resistance; LDL-C: low-density lipoprotein cholesterol; HDL-C: high-density lipoprotein cholesterol; TG: triglyceride.

	* Reduction in HbA1c, %					^‡^ Reduction in FPG, mmol/L				
	β	95% CI	*p*	adj.*p*	N	β	95% CI	*p*	adj.*p*	N
Proportion of female participants, %	0.0068	[0.0018, 0.0119]	0.0082	0.0329	147	0.0073	[3 × 10^−4^, 0.0143]	0.0406	0.2032	137
Age, year	−0.0217	[−0.0379, −0.0056]	0.0083	0.0329	148	−0.0102	[−0.0323, 0.0119]	0.3671	0.7342	139
Baseline BMI, kg/m^2^	−0.0055	[−0.0238, 0.0127]	0.5515	0.5515	148	−0.0054	[−0.0299, 0.0191]	0.6659	0.8324	141
Baseline SBP, mmHg	−0.0051	[−0.0187, 0.0085]	0.4644	0.516	53	−0.0156	[−0.0346, 0.0034]	0.1066	0.3554	49
Baseline DBP, mmHg	0.0251	[0.0022, 0.048]	0.0318	0.0529	52	0.01	[−0.0241, 0.0441]	0.5652	0.8324	48
Baseline HOMA-IR	0.0264	[−0.0198, 0.0726]	0.2633	0.3291	57	0.0057	[−0.0525, 0.0639]	0.8473	0.8918	51
Baseline LDL-C, mmol/L	−0.1058	[−0.2817, 0.0701]	0.2386	0.3291	112	0.0173	[−0.2325, 0.2672]	0.8918	0.8918	106
Baseline HDL-C, mmol/L	−0.6595	[−1.2303, −0.0887]	0.0235	0.0471	123	−1.1985	[−1.991, −0.406]	0.003	0.0304	116
Baseline TG, mmol/L	−0.1881	[−0.331, −0.0452]	0.0099	0.0329	117	−0.1102	[−0.3217, 0.1013]	0.3072	0.7342	111
Baseline fasting insulin, uIU⁄ mL	0.0196	[0.0029, 0.0362]	0.0211	0.0471	77	0.0057	[−0.0198, 0.0311]	0.6627	0.8324	72

**Table 2:** Multivariable meta-regression analysis assessing the association between treatment response and baseline factors. BMI: body mass index; FPG: fasting plasma glucose; SBP: systolic blood pressure; DBP: diastolic blood pressure; HDL-C: high-density lipoprotein cholesterol; TG: triglyceride.

	Reduction in HbA1c, %				Reduction in FPG, mmol/L			
	β	95% CI	*p*	N	β	95% CI	*p*	N
Baseline HbA1c/FPG	0.1881	[0.1051, 0.2711]	<0.0001	109	0.2799	[0.2099, 0.3499]	<0.0001	103
Proportion of female participants, %	0.0066	[0.0012, 0.0121]	0.017	109	0.0112	[0.0019, 0.0205]	0.0178	103
Age, year	−0.0314	[−0.05, −0.0129]	0.0009	109	−0.0011	[−0.0318, 0.0296]	0.9449	103
Baseline BMI, kg/m^2^	−0.0137	[−0.0326, 0.0052]	0.1549	109	−0.0147	[−0.0453, 0.0159]	0.3474	103
Baseline HDL-C, mmol/L	−0.9304	[−1.5176, −0.3431]	0.0019	109	−1.8722	[−2.812, −0.9323]	<0.0001	103
Baseline TG, mmol/L	−0.1265	[−0.2578, 0.0047]	0.0589	109	−0.1438	[−0.3699, 0.0823]	0.2126	103
Duration of the trial, week								
Short (<24 weeks)	Ref				Ref			
Middle (≥24 weeks and <48 weeks)	0.0827	[−0.0429, 0.2083]	0.197	109	−0.0927	[−0.2928, 0.1075]	0.3642	103
Long (≥48 weeks)	0.294	[0.1198, 0.4682]	0.0009	109	−0.0851	[−0.3563, 0.1861]	0.5384	103
Background Therapy								
Monotherapy	Ref				Ref			
Add-on therapy	0.2357	[0.1096, 0.3617]	0.0002	109	0.1568	[−0.031, 0.3445]	0.1017	103

## Data Availability

All data generated in the study are included in the article or uploaded in Supplementary Materials. The original data of the included studies are available in the original publications.

## Supplementary Materials

The following supporting information can be downloaded at: https://www.mdpi.com/article/10.3390/ph19010139/s1, Figure S1: Summary of domain-specific risk of bias assessments; Figure S2: Funnel plot of HbA1c change; Figure S3: Funnel plot of FPG change; Figure S4: Forest plot of HbA1c change; Figure S5: Forest plot of FPG change; Figure S6: Statistically significant factors associated with HbA1c and FPG reduction in glitazone and glitazar subgroups; Figure S7: Partial dependency plot of baseline characteristics with HbA1c change; Figure S8: Partial dependency plot of baseline characteristics with FPG change; Table S1: Summary of characteristics of included studies; Table S2: Basic characteristics of included studies; Table S3: Risk of bias assessment of included studies for HbA1c change; Table S4: Risk of bias assessment of included studies for FPG change; Table S5: Univariable meta-regression analysis of factors associated with treatment response (unadjusted); Table S6: Univariable meta-regression analysis of factors associated with treatment response(multi-variable adjusted); Table S7: Sensitivity analysis of univariable meta regression in glitazone subgroup; Table S8: Sensitivity analysis of univariable meta regression in glitazar subgroup; Table S9: Sensitivity analysis of univariable meta regression in pioglitazone subgroup; Table S10: Sensitivity analysis of univariable meta regression in rosiglitazone subgroup; Table S11: Variance inflation factors in the multivariate regression model; Table S12: Sensitivity analysis of univariable meta regression by excluding trials with high risk of bias; Table S13: Sensitivity analysis of univariable meta regression by excluding trials with participants fewer than 15 per treatment arm; Table S14: Sensitivity analysis of univariable meta regression by excluding trials with imputed standard deviation; Table S15: Sensitivity analysis of univariable meta regression by excluding trials systematically recruiting patients with certain complications; Table S16: Sensitivity analysis of univariable meta regression in monotherapy subgroup; Table S17: Sensitivity analysis of univariable meta regression in add-on therapy subgroup; Table S18: Sensitivity analysis of placebo-corrected univariable meta regression (unadjusted); Table S19: Sensitivity analysis of placebo-corrected univariable meta regression (adjusted for baseline HbA1c/FPG); Table S20: Meta regression of factors associated with placebo response; Table S21: Sensitivity analysis of multivariate meta regression including baseline diastolic blood pressure as covariate. References [6,7,16,17,35,36,37,38,39,40,41,42,43,44,45,46,47,48,49,50,51,52,53,54,55,56,57,58,59,60,61,62,63,64,65,66,67,68,69,70,71,72,73,74,75,76,77,78,79,80,81,82,83,84,85,86,87,88,89,90,91,92,93,94,95,96,97,98,99,100,101,102,103,104,105,106,107,108,109,110,111,112,113,114,115,116,117,118,119,120,121,122,123,124,125,126,127,128,129,130,131,132,133,134,135,136,137,138,139,140,141,142,143,144,145,146,147,148,149,150,151,152,153,154,155,156,157,158,159,160,161,162,163,164,165,166,167,168,169,170,171,172,173,174,175,176,177] are cited in the Supplementary Materials.
