# Supplementary material for: Factors Affecting the Treatment Heterogeneity of PPARγ and Pan-PPAR Agonists in Type 2 Diabetes Mellitus: A Systematic Review and Machine Learning-Based Meta-Regression Analysis
Source: Pharmaceuticals (Basel). 2026 Jan 13;19(1):139. doi: 10.3390/ph19010139 (PMC12845350; doi:10.3390/ph19010139)
Supplement: Supplementary file 1 [file pharmaceuticals-19-00139-s001.zip › pharmaceuticals-4030453-supplementary.pdf]

Supplementary Materials

Table of Content

Section S1 Search Strategy 2
Section S2 Characteristics of included studies 9
Table S1 Summary of characteristics of included studies 9
Table S2 Basic characteristics of included studies 10
Section S3 Risk of bias assessment of included studies for each outcome 28
Table S3 Risk of bias assessment of included studies for HbA1c change 28
Table S4 Risk of bias assessment of included studies for FPG change 33
Fig. S1 Summary of domain-specific risk of bias assessments 37
Section S4 Other main results 38
Fig. S2 Funnel plot of HbA1c change. 38
Fig. S3 Funnel plot of FPG change. 38
Fig. S4 Forest plot of HbA1c change 39
Fig. S5 Forest plot of FPG change 40
Table S5 Univariable meta-regression analysis of factors associated with treatment response (unadjusted) 41
Table S6 Univariable meta-regression analysis of factors associated with treatment response(multi-variable adjusted) 42
Section S5 Sensitivity analysis 43
Fig. S6 Statistically significant factors associated with HbA1c and FPG reduction in glitazone and glitazar subgroups 43
Table S7 Sensitivity analysis of univariable meta regression in glitazone subgroup 44
Table S8 Sensitivity analysis of univariable meta regression in glitazar subgroup 45
Table S9 Sensitivity analysis of univariable meta regression in pioglitazone subgroup 46
Table S10 Sensitivity analysis of univariable meta regression in rosiglitazone subgroup 47
Table S11 Variance inflation factors in the multivariate regression model 47
Table S12 Sensitivity analysis of univariable meta regression by excluding trials with high risk of bias 48
Table S13 Sensitivity analysis of univariable meta regression by excluding trials with participants fewer than 15 per treatment arm 49
Table S14 Sensitivity analysis of univariable meta regression by excluding trials with imputed standard deviation 50
Table S15 Sensitivity analysis of univariable meta regression by excluding trials systematically recruiting patients with certain complications 51
Table S16 Sensitivity analysis of univariable meta regression in monotherapy subgroup 52
Table S17 Sensitivity analysis of univariable meta regression in add-on therapy subgroup 53
Table S18 Sensitivity analysis of placebo-corrected univariable meta regression (unadjusted) 54
Table S19 Sensitivity analysis of placebo-corrected univariable meta regression (adjusted for baseline HbA1c/FPG). 55
Table S20 Meta regression of factors associated with placebo response 56
Table S21 Sensitivity analysis of multivariate meta regression including baseline diastolic blood pressure as covariate 57
Fig. S7 Partial dependency plot of baseline characteristics with HbA1c change 58
Fig. S8 Partial dependency plot of baseline characteristics with FPG change 59
Section S6 Reference list for included studies 60

Section S1 Search Strategy

1.1 Pubmed

#1 "Diabetes Mellitus, Type 2"[Mesh]

#2 NIDDM[TIAB]

#3 "type 2"[TIAB] OR "Type2"[TIAB] OR Type II[TIAB] OR TypeII[TIAB] OR Maturity-Onset[TIAB] OR "Maturity Onset"[TIAB] OR Adult-Onset[TIAB] OR Ketosis-Resistant[TIAB] OR "Ketosis Resistant"[TIAB] OR "Non Insulin"[TIAB] OR Non-Insulin[TIAB] OR Noninsulin[TIAB]

#4 diabet*[TIAB]

#5 #2 OR (#3 AND #4)

#6 #1 OR #5

#7 "PPAR-gamma Agonists"[MeSH]

#8 "Peroxisome Proliferator-Activated Receptor-gamma"[TIAB] OR "PPAR-gamma"[TIAB] OR (("Peroxisome Proliferator Activated Receptor"[TIAB] OR "Peroxisome Proliferator-Activated Receptor"[TIAB] OR "PPAR"[TIAB]) AND gamma[TIAB])

#9 "agonist"[TIAB]

#10 (#8 AND #9) OR #7

#11 ("pan-peroxisome proliferator-activated receptor"[TIAB] OR "pan-peroxisome proliferator activated receptor"[TIAB] OR "pan peroxisome proliferator activated receptor"[TIAB] OR "PAN PPAR"[TIAB] OR "PAN-PPAR"[TIAB]) AND "agonist"[TIAB]

#12 ("peroxisome proliferator-activated receptor"[TIAB] OR "peroxisome proliferator activated receptor"[TIAB] OR "PPAR"[TIAB]) AND ("pan agonist"[TIAB] OR "pan-agonist"[TIAB])

#13 #11 OR #12

#14 "Thiazolidinediones"[Mesh] OR "Thiazolidinediones"[TIAB] OR "TZD"[TIAB] OR "TZDs"[TIAB] OR "Glitazones"[TIAB]

#15 "Troglitazone" [Mesh] OR "Troglitazone"[TIAB] OR "Rezulin"[TIAB] OR "Prelay"[TIAB]

#16 "Pioglitazone" [Mesh] OR "Pioglitazone"[TIAB] OR "Actos"[TIAB]

#17 "Rosiglitazone" [Mesh] OR "Rosiglitazone"[TIAB] OR "Avandia"[TIAB]

#18 "darglitazone" [Supplementary Concept] OR darglitazone[TIAB]

#19 "netoglitazone" [Supplementary Concept] OR netoglitazone[TIAB]

#20 "ciglitazone" [Supplementary Concept] OR "ciglitazone" [TIAB] OR "ciglitizone" [TIAB]

#21 "Balaglitazone" [Supplementary Concept] OR "Balaglitazone"[TIAB]

#22 "rivoglitazone" [Supplementary Concept] OR "rivoglitazone"[TIAB]

#23 "glitazar"[TIAB] OR "glitazars"[TIAB]

#24 "aleglitazar" [Supplementary Concept] OR "aleglitazar"[TIAB]

#25 "tesaglitazar" [Supplementary Concept] OR "tesaglitazar" [TIAB]

#26 "Ragaglitazar" [Supplementary Concept] OR "Ragaglitazar" [TIAB]

#27 "Muraglitazar" [Supplementary Concept] OR "Muraglitazar" [TIAB] OR "Pargluva" [TIAB]

#28 "farglitazar" [Supplementary Concept] OR "farglitazar" [TIAB]

#29 "Chiglitazar" [Supplementary Concept] OR "chiglitazar" [TIAB]

#30 "Saroglitazar" [Supplementary Concept] OR "Saroglitazar" [TIAB] OR "Lipaglyn" [TIAB]

#31 #10 OR #13 OR #14 OR #15 OR #16 OR #17 OR #18 OR #19 OR #20 OR #21 OR #22 OR #23 OR #24 OR #25 OR #26 OR #27 OR #28 OR #29 OR #30

#32 "Glycated Hemoglobin"[Mesh] OR "Hb A1c"[TW] OR "HbA1c"[TW] OR "Glycohemoglobin"[TW] OR

"Glycohaemoglobin"[TW] OR (("Glycated"[TW] OR "Glycosylated"[TW]) AND ("Hemoglobin"[TW] OR "Haemoglobin"[TW]))

#33 ("fasting"[TW] AND "glucose"[TW]) OR "FPG"[TW] OR "FG"[TW]

#34 ("Postprandial"[TW] AND "glucose"[TW]) OR "PPG"[TW] OR "PG"[TW]

#35 "Blood Glucose"[TW] OR "Blood sugar"[TW]

#36 "safe"[TW] OR "safety"[TW] OR tolerability[TW] OR "toxicity"[TW] OR

"side effect"[TW] OR "side effects"[TW] OR "side event "[TW] OR "side events"[TW] OR "side reaction"[TW] OR "side reactions"[TW] OR "adverse event "[TW] OR "adverse events"[TW] OR "adverse reaction"[TW] OR "adverse reactions "[TW]

#37 #32 OR #33 OR #34 OR #35 OR #36

#38 (randomized controlled trial[pt] OR controlled clinical trial[pt] OR randomized[tiab] OR placebo[tiab] OR clinical trials as topic[mesh:noexp] OR randomly[tiab] OR trial[ti] OR randomised[tiab] OR RCT[tiab]) NOT (animals[mh] NOT (humans[mh] AND animals[mh])) NOT (animals[mh] NOT (humans[mh] AND animals[mh])) NOT(Editorial[pt] OR Letter[pt] OR Case Reports[pt] OR Comment[pt] OR Review[pt] OR Systematic Review[pt] OR Meta-analysis)

39 #6 AND #31 AND #37 AND #38

1.2 Embase

#1 'non insulin dependent diabetes mellitus'/exp

#2 'niddm':ab,kw,ti

#3 'type 2':ab,kw,ti OR 'type2':ab,kw,ti OR 'type ii':ab,kw,ti OR 'typeii':ab,kw,ti OR 'maturity-onset':ab,kw,ti OR 'maturity onset':ab,kw,ti OR 'adult-onset':ab,kw,ti OR 'ketosis-resistant':ab,kw,ti OR 'ketosis resistant':ab,kw,ti OR 'non insulin':ab,kw,ti OR 'non-insulin':ab,kw,ti OR 'noninsulin':ab,kw,ti

#4 'diabet*':ab,kw,ti

#5 #2 OR (#3 AND #4)

#6 #1 OR #5

#7 'peroxisome proliferator activated receptor gamma agonist'/exp

#8 'peroxisome proliferator-activated receptor-gamma':ab,kw,ti OR 'ppar-gamma':ab,kw,ti OR (('peroxisome proliferator activated receptor':ab,kw,ti OR 'peroxisome proliferator-activated receptor':ab,kw,ti OR 'ppar':ab,kw,ti) AND 'gamma':ab,kw,ti)

#9 'agonist':ab,kw,ti

#10 (#8 AND #9) OR #7

#11 ('pan-peroxisome proliferator-activated receptor':ab,kw,ti OR 'pan-peroxisome proliferator activated receptor':ab,kw,ti OR 'pan peroxisome proliferator activated receptor':ab,kw,ti OR 'pan ppar':ab,kw,ti OR 'pan-ppar':ab,kw,ti) AND 'agonist':ab,kw,ti

#12 ('peroxisome proliferator-activated receptor':ab,kw,ti OR 'peroxisome proliferator activated receptor':ab,kw,ti OR 'ppar':ab,kw,ti) AND ('pan agonist':ab,kw,ti OR 'pan-agonist':ab,kw,ti)

#13 #11 OR #12

#14 'glitazone derivative'/exp OR 'thiazolidinediones':ab,kw,ti OR 'tzd':ab,kw,ti OR 'tzds':ab,kw,ti OR 'glitazones':ab,kw,ti

#15 'troglitazone'/exp OR 'troglitazone':ab,kw,ti OR 'rezulin':ab,kw,ti OR 'prelay':ab,kw,ti

#16 'pioglitazone'/exp OR 'pioglitazone':ab,kw,ti OR 'actos':ab,kw,ti

#17 'rosiglitazone'/exp OR 'rosiglitazone':ab,kw,ti OR 'avandia':ab,kw,ti

#18 'darglitazone'/exp OR darglitazone:ab,kw,ti

#19 'netoglitazone'/exp OR netoglitazone:ab,kw,ti

#20 'ciglitazone'/exp OR 'ciglitazone':ab,kw,ti OR 'ciglitizone':ab,kw,ti

#21 'balaglitazone'/exp OR 'balaglitazone':ab,kw,ti

#22 'rivoglitazone'/exp OR 'rivoglitazone':ab,kw,ti

#23 'glitazar derivative'/exp OR 'glitazar':ab,kw,ti

#24 'aleglitazar'/exp OR 'aleglitazar':ab,kw,ti

#25 'tesaglitazar'/exp OR 'tesaglitazar':ab,kw,ti

#26 'ragaglitazar'/exp OR 'ragaglitazar':ab,kw,ti

#27 'muraglitazar'/exp OR 'muraglitazar':ab,kw,ti OR 'pargluva':ab,kw,ti

#28 'farglitazar'/exp OR 'farglitazar':ab,kw,ti

#29 'chiglitazar'/exp OR 'chiglitazar':ab,kw,ti

#30 'saroglitazar'/exp OR 'saroglitazar':ab,kw,ti OR 'lipaglyn':ab,kw,ti

#31 #10 OR #13 OR #14 OR #15 OR #16 OR #17 OR #18 OR #19 OR #20 OR #21 OR #22 OR #23 OR #24 OR #25 OR #26 OR #27 OR #28 OR #29 OR #30

#32 'glycated hemoglobin'/exp OR 'hb a1c' OR 'hba1c' OR 'glycohemoglobin' OR 'glycohaemoglobin' OR (('glycated' OR 'glycosylated') AND ('hemoglobin' OR 'haemoglobin'))

#33 'fasting' AND 'glucose' OR 'fpg' OR 'fg'

#34 'postprandial' AND 'glucose' OR 'ppg' OR 'pg'

#35 'blood glucose' OR 'blood sugar'

#36 'safe' OR 'safety' OR tolerability OR 'toxicity' OR 'side effect' OR 'side effects' OR 'side event' OR 'side events' OR 'side reaction' OR 'side reactions' OR 'adverse event' OR 'adverse events' OR 'adverse reaction' OR 'adverse reactions'

#37 #32 OR #33 OR #34 OR #35 OR #36

#38 ('randomized controlled trial'/exp OR randomized:ab,kw,ti OR randomised:ab,kw,ti OR placebo:ab,kw,ti OR randomly:ab,kw,ti OR trial:ti OR RCT:ab,kw,ti) NOT ('conference abstract'/it OR 'editorial'/it OR 'letter'/it OR 'note'/it OR 'review'/it OR 'conference review'/it OR 'erratum'/it) NOT ('meta analysis'/de OR 'systematic review'/de) NOT [animals]/lim

39 #6 AND #31 AND #37 AND #38

1.3 Cochrane

#1 MeSH descriptor: [Diabetes Mellitus, Type 2] explode all trees

#2 'niddm':ab,kw,ti

#3 'type 2':ab,kw,ti OR 'type2':ab,kw,ti OR 'type ii':ab,kw,ti OR 'typeii':ab,kw,ti OR 'maturity-onset':ab,kw,ti OR 'maturity onset':ab,kw,ti OR 'adult-onset':ab,kw,ti OR 'ketosis-resistant':ab,kw,ti OR 'ketosis resistant':ab,kw,ti OR 'non insulin':ab,kw,ti OR 'non-insulin':ab,kw,ti OR 'noninsulin':ab,kw,ti

#4 diabet*:ab,kw,ti

#5 #2 OR (#3 AND #4)

#6 #1 OR #5

#7 MeSH descriptor: [PPAR-gamma Agonists] explode all trees

#8 'peroxisome proliferator-activated receptor-gamma':ab,kw,ti OR 'ppar-gamma':ab,kw,ti OR (('peroxisome proliferator activated receptor':ab,kw,ti OR 'peroxisome proliferator-activated receptor':ab,kw,ti OR 'ppar':ab,kw,ti) AND 'gamma':ab,kw,ti)

#9 'agonist':ab,kw,ti

#10 (#8 AND #9) OR #7

#11 ('pan-peroxisome proliferator-activated receptor':ab,kw,ti OR 'pan-peroxisome proliferator activated receptor':ab,kw,ti OR 'pan peroxisome proliferator activated receptor':ab,kw,ti OR 'pan ppar':ab,kw,ti OR 'pan-ppar':ab,kw,ti) AND 'agonist':ab,kw,ti

#12 ('peroxisome proliferator-activated receptor':ab,kw,ti OR 'peroxisome proliferator activated receptor':ab,kw,ti OR 'ppar':ab,kw,ti) AND ('pan agonist':ab,kw,ti OR 'pan-agonist':ab,kw,ti)

#13 #11 OR #12

#14 MeSH descriptor: [Thiazolidinediones] explode all trees

#15 'thiazolidinediones':ab,kw,ti OR 'tzd':ab,kw,ti OR 'tzds':ab,kw,ti OR 'glitazones':ab,kw,ti

#16 'troglitazone':ab,kw,ti OR 'rezulin':ab,kw,ti OR 'prelay':ab,kw,ti

#17 'rosiglitazone':ab,kw,ti OR 'avandia':ab,kw,ti

#18 'pioglitazone':ab,kw,ti OR 'actos':ab,kw,ti

#19 darglitazone:ab,kw,ti

#20 netoglitazone:ab,kw,ti

#21 'ciglitazone':ab,kw,ti OR 'ciglitizone':ab,kw,ti

#22 'balaglitazone':ab,kw,ti

#23 'rivoglitazone':ab,kw,ti

#24 'glitazar':ab,kw,ti OR 'glitazars':ab,kw,ti

#25 'aleglitazar':ab,kw,ti

#26 'tesaglitazar':ab,kw,ti

#27 'ragaglitazar':ab,kw,ti

#28 'muraglitazar':ab,kw,ti OR 'pargluva':ab,kw,ti

#29 'farglitazar':ab,kw,ti

#30 'chiglitazar':ab,kw,ti

#31 'saroglitazar':ab,kw,ti OR 'lipaglyn':ab,kw,ti

#32 #10 OR #13 OR #14 OR #15 OR #16 OR #17 OR #18 OR #19 OR #20 OR #21 OR #22 OR #23 OR #24 OR #25 OR #26 OR #27 OR #28 OR #29 OR #30 OR #31

#33 MeSH descriptor: [Glycated Hemoglobin] explode all trees

#34 'hb a1c' OR 'hba1c' OR 'glycohemoglobin' OR 'glycohaemoglobin' OR (('glycated' OR 'glycosylated') AND ('hemoglobin' OR 'haemoglobin'))

#35 'fasting' AND 'glucose' OR 'fpg' OR 'fg'

#36 'postprandial' AND 'glucose' OR 'ppg' OR 'pg'

#37 'blood glucose' OR 'blood sugar'

#38 'safe' OR 'safety' OR tolerability OR 'toxicity' OR 'side effect' OR 'side effects' OR 'side event' OR 'side events' OR 'side reaction' OR 'side reactions' OR 'adverse event' OR 'adverse events' OR 'adverse reaction' OR 'adverse reactions'

#39 #33 OR #34 OR #35 OR #36 OR #37 OR #38

#40 MeSH descriptor: [Randomized Controlled Trial] explode all trees

#41 randomized:ab,kw,ti OR randomised:ab,kw,ti OR placebo:ab,kw,ti OR randomly:ab,kw,ti OR trial:ti OR RCT:ab,kw,ti

#42 #40 OR #41

#43 #6 AND #32 AND #39 AND #42

#44 #43 in Trials

1.4 Clinicaltrials.gov

peroxisome proliferator activated receptor gamma agonist OR pan-peroxisome proliferator activated receptor agonist OR thiazolidinediones OR Troglitazone OR Rosiglitazone OR pioglitazone OR darglitazone OR netoglitazone OR ciglitazone OR balaglitazone OR rivoglitazone OR glitazar OR aleglitazar OR tesaglitazar OR ragaglitazar OR muraglitazar OR farglitazar OR chiglitazar OR saroglitazar | Studies With Results | Interventional Studies | Diabetes Mellitus, Type 2

1.5 WHO ICTRP

((thiazolidinedione* OR glitazone* OR glitazar* OR troglitazone OR rosiglitazone OR pioglitazone OR darglitazone OR netoglitazone OR ciglitazone OR balaglitazone OR rivoglitazone OR aleglitazar OR tesaglitazar OR ragaglitazar OR muraglitazar OR farglitazar OR chiglitazar OR saroglitazar) AND ("type 2 diabetes" OR T2DM))

Section S2 Characteristics of included studies

Table S1 Summary of characteristics of included studies

		Glitazones	Glitazars	Overall
Study characteristics				
No. of treatment arms		146	16	162
No. of participants (median [IQR])		69.50 [30.00, 160.50]	269.00 [135.75, 415.25]	82.50 [32.00, 174.25]
Treatment duration, n (%)	< 24 weeks	58 (39.7)	6 (37.5)	64 (39.5)
	>=24 weeks and <48 weeks	65 (44.5)	9 (56.2)	74 (45.7)
	>= 48 weeks	23 (15.8)	1 (6.2)	24 (14.8)
Participants				
% of female (median [IQR])		46.70 [38.00, 52.94]	47.73 [43.00, 52.81]	46.75 [39.52, 53.04]
Age, years (mean (SD))		56.88 (3.60)	55.38 (3.30)	56.73 (3.59)
BMI (median [IQR])		29.50 [25.70, 31.30]	31.00 [30.43, 31.71]	29.65 [25.80, 31.39]
Duration of diabetes, years (median [IQR])		6.00 [4.64, 8.52]	5.20 [2.62, 6.59]	6.00 [4.50, 8.16]
Baseline HbA1c, % (mean (SD))		8.37 (0.83)	8.02 (0.46)	8.33 (0.81)
Baseline FPG, mmol/L (median [IQR])		9.83 [8.80, 10.83]	9.25 [9.09, 9.62]	9.74 [8.80, 10.75]
Baseline SBP, mmHg (mean (SD))		133.88 (7.86)	130.75 (8.70)	133.77 (7.82)
Baseline DBP, mmHg (mean (SD))		80.02 (4.35)	77.80 (3.39)	79.94 (4.31)
Baseline HOMA-IR (median [IQR])		4.78 [4.09, 5.98]	4.88 [4.69, 5.24]	4.78 [4.10, 5.90]
Baseline LDL-C, mmol/L (median [IQR])		3.07 [2.83, 3.29]	2.92 [2.79, 3.15]	3.04 [2.82, 3.29]
Baseline HDL-C, mmol/L (mean (SD))		1.17 (0.12)	1.16 (0.05)	1.17 (0.12)
Baseline triglyceride, mmol/L (median [IQR])		2.02 [1.77, 2.29]	2.08 [1.77, 2.17]	2.03 [1.77, 2.28]
Baseline fasting insulin, uIU/mL (median [IQR])		12.96 [10.42, 16.14]	13.95 [11.81, 14.65]	12.98 [10.50, 15.81]

Data are presented as means ± SD or median [IQR] for continuous variables and percentage for categorical variables. SD: standard deviation; IQR: interquartile range; BMI: body mass index; FPG: fasting plasma glucose; SBP: systolic blood pressure; DBP: diastolic blood pressure; HOMA-IR: homeostatic Model Assessment of Insulin Resistance; LDL-C: low-density lipoprotein cholesterol; HDL-C: high-density lipoprotein cholesterol.

Table S2 Basic characteristics of included studies

Study identifier (unique trial registration number)	Treatment duration, weeks	Drug class	Study arm included in the meta-analysis	Background therapy	No. of patients randomized	Proportion of female participants, %	Age, years	BMI, kg/m^2	Diabetes duration, years	Baseline HbA1c, %	Baseline FPG, mmol/L
Davidson 2007[1]	24	Glitazone	rosiglitazone 8 mg daily	sulfonylurea	117	54.7	52 ± 11.9	31.3 ± 5.7	6 ± 5.2	9.2 ± 1.3	10.8 ± 2.7
		Placebo	placebo	sulfonylurea	116	51.7	53 ± 10.4	31.9 ± 5.6	6.2 ± 5.3	9.4 ± 1.4	10.8 ± 2.8
Chou 2012 (NCT00484198)[2]	26	Glitazone	rivoglitazone 1-1.5 mg daily	none	1024	49.8	55.1 ± 10.6	29.6 ± 5.4	4.5 ± 4.7	7.7 ± 0.6	8.9 ± 2.3
		Glitazone	pioglitazone 45 mg daily	none	751	47	55 ± 10.8	30 ± 5.8	4.4 ± 5	7.7 ± 0.6	9 ± 2.4
		Placebo	placebo	none	137	51.1	55.4 ± 12.3	30.1 ± 5.4	4.9 ± 6.1	7.7 ± 0.5	8.9 ± 2.5
Truitt 2010[3]	26	Glitazone	rivoglitazone 1-2 mg daily	none	172	45.3	55.2 ± 10.9	33 ± 6.2	5.7 ± 5.4	8 ± 0.8	9.7 ± 2.7
		Glitazone	pioglitazone 45 mg daily	none	92	41.8	56.6 ± 10.1	32.9 ± 5.7	6.6 ± 7.5	8 ± 0.8	9.5 ± 2.5
		Placebo	placebo	none	92	48.9	55.3 ± 9.3	32.2 ± 5.8	6.7 ± 5.6	8.2 ± 1	9.8 ± 2.5
Goldberg 2005[4]	12	Glitazone	pioglitazone 30 mg daily	none	369	46.1	55.9 ± 10.5	33.7 ± 12.9	3.9 ± 4.4	7.6 ± 1.2	10 ± 3.2
		Glitazone	rosiglitazone 4 mg daily	none	366	45.1	56.3 ± 11.3	32.6 ± 6.6	4 ± 4.6	7.5 ± 1.2	9.8 ± 3.1
John 2002[5]	52	Glitazone	rosiglitazone 8 mg daily	none	104	25	55.1 ± 9	NA	5.3 ± 6.2	9.1 ± 1.7	NA
Bays 2007	24	Glitazar	tesaglitazar	none	683	47.7	58.2 ± NA	32 ± 6.6	4.7 ± NA	7.2 ± 1	8.6 ± 2.3

(NCT00214565)[6]			0.5-1 mg daily								
		Glitazone	pioglitazone 15-45 mg daily	none	1024	52.6	56.8 ± NA	31.8 ± 6.4	4.5 ± NA	7.2 ± 1.1	8.7 ± 2.4
Ryang 2022 (NCT03770052)[7]	24	Glitazone	lobeglitazone 0.25-0.5 mg daily	metformin and DPP-4 inhibitor	147	51.6	61.4 ± 8.7	25.4 ± 3.2	9.7 ± 6.1	7.7 ± 0.5	8.4 ± 1.4
Hanefeld 2007[8]	52	Glitazone	rosiglitazone 4-8 mg daily	none	189	37	60.5 ± 8.7	28.8 ± 3.7	5.9 ± 6.5	8.2 ± 1.4	10.7 ± 3.3
Raskin 2001[9]	26	Glitazone	rosiglitazone 4-8 mg daily	insulin	209	44.5	57.4 ± 10.1	32.2 ± 4.9	12.6 ± 7.7	9.1 ± 1.3	11.7 ± 3.2
		Placebo	placebo	insulin	104	44.3	55.6 ± 10.3	32.7 ± 4.5	11.7 ± 6.2	8.9 ± 1.1	10.8 ± 2.9
Lü 2011[10]	16	Glitazone	pioglitazone 30 mg daily	sulfonylurea	119	47.9	56.3 ± 8.2	25.1 ± 3.1	5.8 ± 4.4	8.6 ± 1.1	9.8 ± 1.8
		Placebo	placebo	sulfonylurea	117	48.7	55.7 ± 8.3	24.6 ± 3	6.3 ± 4.6	8.8 ± 1.2	9.6 ± 1.6
Herz 2003[11]	16	Glitazone	pioglitazone 30-45 mg daily	none	198	44	58.5 ± 11	31.2 ± 4.9	1.8 ± 3.4	7.6 ± 0.8	NA
		Placebo	placebo	none	99	50.5	58 ± 10.7	31.7 ± 4.5	1.4 ± 2.5	7.5 ± 0.3	NA
Kong 2011 (NCT00575874)[12]	12	Glitazone	pioglitazone 30 mg daily	none	37	43.2	53.6 ± 7.6	24.9 ± 3.3	5.6 ± 4.6	7.5 ± 0.8	9.7 ± 2.1
		Glitazone	rivoglitazone 1-1.5 mg daily	none	69	43.5	52.8 ± 8.5	25.8 ± 3.9	4.7 ± 3.4	7.4 ± 0.7	9.2 ± 2.2
		Placebo	placebo	none	32	40.6	54 ± 8.5	25.5 ± 4	5.8 ± 3.9	7.3 ± 0.6	9.2 ± 1.8
Wolffenbuttel 2000[13]	24	Glitazone	rosiglitazone 4 mg daily	sulfonylurea	382	42.4	60.8 ± 9	28.1 ± 3.9	7 ± NA	9.2 ± 1.2	11.4 ± 2.7

		Placebo	placebo	sulfonylurea	192	42.7	61.9 ± 9.1	28.1 ± 4.1	8 ± NA	9.2 ± 1.3	11.5 ± 2.4
Davidson 2006[14]	24	Glitazone	pioglitazone 30-45 mg daily	insulin	345	45	56.4 ± 10.7	33.2 ± 5.5	13.1 ± NA	9.8 ± 1.6	11.1 ± 4.2
Zhu 2003[15]	24	Glitazone	rosiglitazone 4-8 mg daily	sulfonylurea	425	55.5	59 ± 7.2	24.8 ± 3.3	7.5 ± NA	9.8 ± 1.6	10.2 ± 2
		Placebo	placebo	sulfonylurea	105	54	58.8 ± 7.7	25.1 ± 2.8	7.6 ± NA	9.8 ± 1.3	10.3 ± 1.8
Stirban 2016 (NCT01729403)[16]	16	Glitazar	aleglitazar 0.15 mg daily	metformin	29	20.7	59.4 ± 8.4	31.4 ± 3.3	8.6 ± 7.2	7.3 ± 0.5	8.4 ± 2.6
		Placebo	placebo	metformin	28	21.4	60.1 ± 7	30.2 ± 2.8	9.6 ± 8	7.6 ± 0.6	9.2 ± 1.6
Wallace 2003[17]	12	Glitazone	pioglitazone 30-45 mg daily	none	19	26.3	61.4 ± NA	29.8 ± NA	2.6 ± NA	6.7 ± 0.9	7.4 ± 1.6
		Placebo	placebo	none	11	27.3	62.6 ± NA	28.9 ± NA	2.5 ± NA	6.7 ± 0.9	7.9 ± 1.2
Vongthavaravat 2002[18]	26	Glitazone	rosiglitazone 4 mg daily	sulfonylurea	164	54.3	54.6 ± NA	27.1 ± NA	NA	9.1 ± 1	11.1 ± 2.4
		Placebo	placebo	sulfonylurea	170	57.6	57.3 ± NA	27.1 ± NA	NA	8.9 ± 1.2	10.8 ± 2.5
Satoh 2003[19]	12	Glitazone	pioglitazone 30 mg daily	sulfonylurea /diet	70	54.3	61.2 ± 10.9	23.4 ± 3.3	NA	8.1 ± 0.8	9.6 ± 3.3
Kadoglou 2007[20]	26	Glitazone	rosiglitazone 8 mg daily	sulfonylurea and metformin	35	60	63.8 ± 7.3	29.5 ± 3.8	8.5 ± 4.6	8.2 ± 1.2	10.7 ± 2.3
		Placebo	placebo	sulfonylurea and metformin	35	54.3	66.7 ± 9.6	29.9 ± 4.3	7.5 ± 5.9	8 ± 0.8	11 ± 1
Bertrand 2010 (NCT00169832)[21]	52	Glitazone	rosiglitazone 4-8 mg daily	not specified	95	8	64.2 ± 7.3	30.2 ± 4.2	NA	6.9 ± 0.8	7.7 ± 2

		Placebo	placebo	not specified	98	8	65.1 ± 6.9	29.5 ± 4.6	NA	6.9 ± 1.3	7.5 ± 1.6
Jia 2021 (NCT02173457)[22]	24	Glitazar	chiglitazar 32-48 mg daily	none	491	49.1	50.9 ± 9.6	25.7 ± 3.1	1.4 ± 2.3	8.5 ± 0.7	9.2 ± 2.3
Wei 2013[23]	12	Glitazone	rosiglitazone 4 mg daily	not specified	20	30	60.6 ± 9.6	25.2 ± 4	NA	7.8 ± 1.6	7.3 ± 1.7
		Placebo	placebo	not specified	20	30	60.4 ± 8.5	24.4 ± 2.8	NA	7.5 ± 1.8	7.4 ± 2.9
Raskin 2004[24]	24	Glitazone	rosiglitazone 4-8 mg daily	none	62	46.8	56.6 ± 10.8	31.4 ± 5.2	7.4 ± 6.6	9 ± 1.3	14 ± 4
Rosenstock 2002[25]	16	Glitazone	troglitazone 600 mg daily	none	151	37	57 ± NA	NA	NA	8.1 ± 1	10.3 ± 2.3
		Placebo	placebo	none	148	41	58 ± NA	NA	NA	8.2 ± 1.2	10.4 ± 2.5
Veleba 2015 (EudraCT number 2009-011106-42)[26]	24	Glitazone	pioglitazone 15 mg daily	metformin	18	NA	62.4 ± 4	32.4 ± 4.7	NA	NA	7.7 ± 1.1
		Placebo	placebo	metformin	17	NA	61.6 ± 5.8	30.6 ± 4.8	NA	NA	7.3 ± 1.6
Esteghamati 2014 (NCT02027103)[27]	12	Glitazone	pioglitazone 30 mg daily	none	54	50	51.5 ± 8.7	NA	NA	8 ± 1.3	9.9 ± 2.8
Raman 2023[28]	52	Glitazone	pioglitazone 15 mg daily	metformin and DPP-4 inhibitor	54	53.7	58.2 ± 6.6	25.3 ± 3.3	8.6 ± 4.2	8 ± 1.1	9.7 ± 1.5
		Glitazone	lobeglitazone 0.5 mg daily	metformin and DPP-4 inhibitor	51	52.9	56.5 ± 6.6	25.6 ± 5.8	8.1 ± 4.8	8.1 ± 1	9.8 ± 1.6
Kato 2009[29]	12	Glitazone	pioglitazone	none	25	52	51.4 ± 15.2	28.4 ± 6.4	NA	7.4 ± 1.8	7.3 ± 1.4

Takihata 2013 (UMIN 000004716)[30]	24	Glitazone	15 mg daily pioglitazone 15-30 mg daily	metformin and/or sulfonylurea	65	43.9	60.7 ± 9.5	25.8 ± 4.8	NA	7.4 ± 0.6	7.9 ± 1.9
Jameshorani 2017 (IRCT201506161955 4N4)[31]	12	Glitazone	pioglitazone 30 mg daily	metformin	80	71.3	55.1 ± 5.8	NA	NA	8.6 ± 0.6	9.5 ± 3.1
Yoneda 2021[32]	24	Glitazone	pioglitazone 15-30 mg daily	none	19	57.9	58.8 ± 8.1	30.8 ± 1.1	NA	7.1 ± 0.6	8 ± 2.3
Erem 2014[33]	52	Glitazone	pioglitazone 15-45 mg daily	none	19	73.7	52.5 ± 5.2	31.3 ± 4.7	NA	8 ± 1.7	9.2 ± 1.9
Hamann 2007[34]	52	Glitazone	rosiglitazone 4 mg daily	metformin	294	47	58.5 ± 9.6	33 ± 5.9	6.3 ± 5.4	8 ± 0.9	10.5 ± 2.8
Perriello 2006[35]	52	Glitazone	pioglitazone 30-45 mg daily	none	146	33.6	58 ± 8	29.2 ± 3.1	9.8 ± 5.4	8.8 ± 0.8	10.5 ± 1.9
Strowig 2002[36]	16	Glitazone	troglitazone 200-600 mg daily	insulin	30	56.7	51.7 ± 8	36.4 ± 6	11.6 ± 6.8	8.5 ± 1.2	10.3 ± 3.4
Yamanouchi 2005[37]	52	Glitazone	pioglitazone 30-45 mg daily	none	38	52.6	55.2 ± 9.2	25.8 ± 4.2	NA	10.2 ± 0.8	12 ± 1.9
Fujitaka 2011[38]	26	Glitazone	pioglitazone 30 mg daily	none	30	33.4	61 ± 11	27.7 ± 4.5	NA	6.2 ± 0.8	7.2 ± 2.9
Nagasaka 2003[39]	16	Glitazone	pioglitazone 15-30 mg	sulfonylurea	35	47.2	56 ± 9	25.4 ± 1.5	6.3 ± 3.4	8.3 ± 0.6	8.9 ± 0.6

			daily								
Xu 2015 (NCT01147627)[40]	48	Glitazone	pioglitazone 30-45 mg daily	none	136	44.9	NA	25.9 ± 3.3	NA	8 ± 1.1	9.2 ± 2.2
Yoon 2011[41]	48	Glitazone	rosiglitazone NA mg daily	none	117	47.9	50.1 ± 8.2	25.8 ± 3.3	NA	7.8 ± 0.8	8 ± 1.1
Baksi 2003[42]	26	Glitazone	rosiglitazone 8 mg daily	sulfonylurea	225	42.7	61.1 ± 9.1	30.2 ± 5.7	6.5 ± 5.9	8.5 ± 1.5	10.3 ± 2.5
Kim 2016 (NCT01882907)[43]	16	Glitazone	pioglitazone 15 mg daily	metformin	111	56.8	53.9 ± 9.1	25 ± 3.3	5 ± 4.9	8.1 ± 1	8.4 ± 2
Bolli 2008[44]	52	Glitazone	pioglitazone 30 mg daily	metformin	281	35.9	57 ± 9.7	32.1 ± 5.1	6.4 ± 5.2	8.4 ± 0.9	11 ± 2.7
Reynolds 2007[45]	24	Glitazone	rosiglitazone 4-8 mg daily	metformin and sulfonylurea	20	NA	NA	30.7 ± 5.1	NA	9.1 ± 0.9	NA
Virtanen 2003[46]	26	Glitazone	rosiglitazone 8 mg daily	none	14	28.6	58 ± 7.5	29.1 ± 4.1	NA	6.8 ± 0.7	7.2 ± 1
		Placebo	placebo	none	14	28.6	58 ± 7.5	30.3 ± 4.4	NA	6.3 ± 0.3	7.2 ± 1
Derosa 2009[47]	65	Glitazone	pioglitazone 15-45 mg daily	none	69	53.6	54 ± 6	27.5 ± 1.7	NA	9.2 ± 1.3	9.1 ± 1.6
Miyazaki 2002[48]	26	Glitazone	pioglitazone 15-45 mg daily	none	34	32.3	54.4 ± 9.9	31.1 ± 4.4	NA	8.5 ± 1.3	11.7 ± 2.9
		Placebo	placebo	none	11	72.7	58 ± 9.9	32.8 ± 5.3	NA	8.6 ± 1.7	11 ± 4.1
Iwamoto 1995[49]	12	Glitazone	troglitazone 200 mg daily	sulfonylurea	122	49.2	57.8 ± 9	23.7 ± 3.4	NA	9.2 ± 1.4	10.8 ± 2

Rajagopalan 2015[50]	12	Glitazone	pioglitazone 15-30 mg daily	metformin and/or sulfonylurea	60	48.3	51.6 ± 9.6	26.4 ± 4.1	3.5 ± 1.7	8.4 ± 0.8	9.3 ± 1.6
Fonseca 2000[51]	26	Glitazone	pioglitazone 4-8 mg daily	metformin	226	34.9	57.9 ± 9.7	30 ± 4	7.9 ± 6.3	8.9 ± 1.4	12 ± 3.1
		Placebo	placebo	none	116	25.7	58.8 ± 9.2	30.3 ± 4.4	7.3 ± 5.7	8.6 ± 1.3	11.9 ± 2.9
Yang 2014[52]	12	Glitazone	pioglitazone 30 mg daily	none	30	33.4	47 ± 10	26 ± 2	NA	8.3 ± 0.3	8.8 ± 1.4
Derosa 2010[53]	40	Glitazone	pioglitazone 15-45 mg daily	metformin and sulfonylurea	175	51.4	55 ± 8	26.2 ± 0.6	NA	7.9 ± 0.5	7.4 ± 0.7
Pavo 2003[54]	32	Glitazone	pioglitazone 30-45 mg daily	none	105	43.8	54.2 ± 9.1	31.3 ± 4.2	0.5 ± 0.3	8.6 ± 1.1	11.8 ± 3.3
Hao 2005[55]	12	Glitazone	pioglitazone 15 mg daily	sulfonylurea	35	45.7	62.5 ± 9.5	24.2 ± 2.9	6.2 ± 5.7	9.3 ± 1.5	10.6 ± 2.9
		Placebo	placebo	sulfonylurea	35	48.6	64.4 ± 10	23.9 ± 3.2	6.5 ± 5.2	9.2 ± 1.5	10.3 ± 2.2
Genovese 2013[56]	16	Glitazone	pioglitazone 30-45 mg daily	none	24	51.7	59.1 ± 6.8	31.1 ± 3.2	NA	6.9 ± 0.9	8.5 ± 2.2
Miyazaki 2001[57]	12	Glitazone	rosiglitazone 8 mg daily	none	15	53.3	54 ± 7.7	30 ± 4.3	NA	8.7 ± 1.5	10.8 ± 2.4
		Placebo	placebo	none	14	40	56 ± 7.5	30.1 ± 3.7	NA	8.3 ± 1.5	10.4 ± 1.7
Carey 2002[58]	16	Glitazone	rosiglitazone 8 mg daily	none	16	12.5	54.2 ± 11.1	29.8 ± 4	3.3 ± 4.5	7.8 ± 1.3	9.3 ± 1.3
		Placebo	placebo	none	17	23.5	57.9 ± 10	31.3 ± 3.6	3.1 ± 3.3	7.1 ± 1.4	7.8 ± 2.3
Goldstein 2006 (NCT00280865) [59]	12	Glitazar	tesaglitazar 0.5-1 mg	none	143	43.4	57.5 ± 10	30.5 ± 5.1	NA	7.3 ± 1.1	9.5 ± 1.8

		Glitazone	daily pioglitazone 45 mg daily	none	72	34.7	58.9 ± 11.7	29.7 ± 4.4	NA	7 ± 0.9	9.2 ± 1.7
		Placebo	placebo	none	70	38.6	56.1 ± 10.6	30.6 ± 3.9	NA	7 ± 0.8	9.3 ± 1.8
Henry 2009 (NCT00388518)[60]	16	Glitazar	aleglitazar 0.15-0.6 mg daily	none	165	57.3	55.5 ± 9.8	31.5 ± 7	2.8 ± 0.5	7.9 ± 0.7	9.7 ± 2.1
		Glitazone	pioglitazone 45 mg daily	none	57	58	54.4 ± 8.8	30.8 ± 5.5	2.7 ± NA	8 ± 0.8	10.2 ± 1.9
		Placebo	placebo	none	55	58	56.5 ± 8.9	32.5 ± 7.5	2.9 ± NA	8.1 ± 0.8	9.8 ± 2.5
Triwatana 2022[61]	16	Glitazone	pioglitazone 15 mg daily	not specified	22	27.3	66.6 ± 8.3	26.1 ± 3.7	NA	6.7 ± 0.6	7.4 ± 1.5
		Placebo	placebo	not specified	24	33.3	71.8 ± 6.5	25.5 ± 2.7	NA	6.7 ± 1	7 ± 1.9
Derosa 2006[62]	52	Glitazone	pioglitazone 15 mg daily	sulfonylurea	45	53.3	53 ± 6	24.4 ± 0.8	5 ± 2	8.2 ± 0.7	9.1 ± 1.7
		Glitazone	rosiglitazone 4 mg daily	sulfonylurea	42	47.6	54 ± 5	24.3 ± 0.7	6 ± 3	8 ± 0.8	8.8 ± 1.8
Hartemann-Heurtier 2009[63]	24	Glitazone	pioglitazone 30-45 mg daily	metformin and sulfonylurea	14	35.7	62 ± 10	30 ± 5	12 ± 4.5	8.3 ± 0.5	8.5 ± 2.2
Sathyanarayana 2011[64]	52	Glitazone	pioglitazone 30-45 mg daily	diet or metformin	10	NA	NA	29.7 ± 1.9	NA	8.3 ± 1.3	10.9 ± 3.7
Wang 2004[65]	12	Glitazone	pioglitazone 15-30 mg daily	sulfonylurea	27	40.7	56.3 ± 11.8	24.6 ± 2.9	NA	9.3 ± 2	9.1 ± 1.5
Bhagat 2022[66]	16	Glitazone	pioglitazone	metformin	25	36	52.2 ± 9.5	28.7 ± 3.7	4.5 ± 3.6	8.2 ± 0.8	9.8 ± 1.7

Defronzo 2010[67]	20	Glitazone	30 mg daily rosiglitazone 4-8 mg daily	metformin	45	51	56 ± 10	32.5 ± 4.3	4.7 ± 3.7	7.9 ± 0.7	8.5 ± 1.8
Xiao 2015[68]	24	Glitazone	pioglitazone 15-45 mg daily	metformin	34	41.2	54.1 ± 4.9	26.5 ± 1.3	NA	8.7 ± 0.8	9.3 ± 0.8
Naka 2012[69]	26	Glitazone	pioglitazone 30 mg daily	sulfonylurea	15	67	63 ± 10	31.9 ± 4.2	7.5 ± 6.3	7.8 ± 0.9	8.1 ± 1.6
Teramoto 2007[70]	24	Glitazone	pioglitazone 15-30 mg daily	none	46	28.3	57 ± 10.7	24.7 ± 3.4	NA	8 ± 1.3	10.5 ± 4.2
Sourij 2006[71]	12	Glitazone	pioglitazone 30 mg daily	none	21	NA	NA	NA	NA	6.1 ± 0.6	6.2 ± 0.9
		Placebo	placebo	none	21	NA	NA	NA	NA	6.1 ± 0.5	5.9 ± 0.7
Weissman 2005[72]	24	Glitazone	rosiglitazone 4-8 mg daily	metformin	382	48.9	55.5 ± 11.2	34.4 ± 7.3	NA	8.1 ± 1.2	10.3 ± 3.1
Iwamoto 1996[73]	12	Glitazone	troglitazone 400 mg daily	none	136	49.3	54.6 ± 10.1	24.1 ± 3.5	6.3 ± 4.4	8.6 ± 1.5	10.1 ± 1.6
		Placebo	placebo	none	126	46.8	54.7 ± 9.3	24.7 ± 3.4	7.5 ± 5.4	8.5 ± 1.5	10.1 ± 1.8
Erande 2013[74]	12	Glitazone	pioglitazone 15 mg daily	metformin	65	NA	49.2 ± 9.6	25.4 ± 3.9	NA	8.4 ± 0.9	7.8 ± 2.1
Liu 2013 (NCT01195090)[75]	24	Glitazone	pioglitazone 30 mg daily	metformin and sulfonylurea	60	61.7	58.1 ± 8.3	25.7 ± 3.7	7.8 ± 3.9	8.5 ± 1	10.1 ± 2.1
Ji 2021 (NCT02121717)[76]	24	Glitazar	chiglitazar 32-48 mg daily	none	333	38.1	51.6 ± 9.7	26.2 ± 3.2	1.3 ± 2.3	8.5 ± 0.7	9.3 ± 2.1

		Placebo	placebo	none	202	38.6	51.2 ± 10	26.1 ± 3	1.4 ± 2.5	8.6 ± 0.7	9.3 ± 2.3
Jones 2012[77]	26	Glitazone	pioglitazone 45 mg daily	none	163	40.5	55.3 ± 11	31.1 ± 5.3	2.7 ± 3.7	8.5 ± 1.2	NA
Bergenstal 2010 (NCT00637273)[78]	26	Glitazone	pioglitazone 45 mg daily	metformin	165	52	53 ± 10	32 ± 6	6 ± 5	8.5 ± 1.1	9.1 ± 2.4
Kim 2014 (NCT01001611)[79]	24	Glitazone	lobeglitazone 0.5 mg daily	none	115	42.6	56.4 ± 9.3	25.2 ± 2.8	NA	7.8 ± 0.9	8.4 ± 2.1
		Placebo	placebo	none	58	44.8	54.7 ± 9.7	25.1 ± 2.2	NA	8.1 ± 0.9	9.1 ± 3.6
Rubin 2009 (NCT00240383)[80]	12	Glitazar	muraglitazar 5 mg daily	none	245	NA	NA	NA	NA	8.2 ± NA	10.1 ± 2.8
		Glitazone	pioglitazone 15 mg daily	none	251	NA	NA	NA	NA	8.3 ± NA	10.1 ± 3.2
Kaku 2015[81]	16	Glitazone	pioglitazone 15-30 mg daily	DPP-4 inhibitor	141	28.4	60 ± 10.3	25.6 ± 4.2	8 ± 6.3	8 ± 1	8.5 ± 2
		Placebo	placebo	DPP-4 inhibitor	69	29	60.5 ± 8.7	24.8 ± 3.2	7.7 ± 6	8.2 ± 0.9	8.6 ± 2.1
Rosenstock 2002[82]	16	Glitazone	pioglitazone 15-30 mg daily	insulin	379	51.7	57.2 ± 10.2	33.8 ± 5.8	NA	9.8 ± 1.3	12.3 ± 3.9
		Placebo	placebo	insulin	187	54.5	56.7 ± 9.4	33.2 ± 5.2	NA	9.8 ± 1.3	12.2 ± 3.9
Kim 2020 (NCT02426294)[83]	26	Glitazone	pioglitazone 15-30 mg daily	metformin and DPP-4 inhibitor	69	50.7	60.7 ± 9.1	24.4 ± 3.2	10.6 ± 8.2	8.2 ± 0.6	9.5 ± 1.7
Schernthaner 2004[84]	52	Glitazone	pioglitazone 30-45 mg daily	none	597	47.4	57 ± 9.4	31.2 ± 4.9	3.4 ± 4.3	8.7 ± 1	11.4 ± 2.7

Perez 2009 (NCT00727857)[85]	24	Glitazone	pioglitazone 30 mg daily	none	189	65.1	54 ± 12.8	31.2 ± 5.5	NA	8.7 ± 1	9.5 ± 3.1
Perez 2002[86]	26	Glitazone	rosiglitazone 4-8 mg daily	metformin	71	76.1	53 ± 9.1	27.8 ± 3.6	10.9 ± 7	10 ± 1.8	13 ± NA
		Placebo	placebo	metformin	34	70.6	53.4 ± 7.5	28.5 ± 3.9	9.1 ± 5.6	9.8 ± 1.6	12.5 ± NA
Scott 2007 (NCT00541775)[87]	18	Glitazone	rosiglitazone 8 mg daily	metformin	87	37	54.8 ± 10.5	30.4 ± 5.5	4.6 ± 4	7.7 ± 0.8	8.7 ± 1.8
		Placebo	placebo	metformin	92	41	55.3 ± 9.3	30 ± 4.5	5.4 ± 3.7	7.7 ± 0.9	8.9 ± 2.1
Rodrigues 2022[88]	12	Glitazone	pioglitazone 15 mg daily	metformin and sulfonylurea	25	33.4	57.6 ± 7.7	25.8 ± 2.6	NA	8.8 ± 0.8	9.8 ± 0.9
Henriksen 2011 (NCT00515632)[89]	26	Glitazone	pioglitazone 45 mg daily	insulin	102	31	60.1 ± 8.6	33.2 ± 5	13.8 ± 7.4	8.7 ± 1.4	9.7 ± 3.3
		Glitazone	balaglitazone 10-20 mg daily	insulin	192	36.5	60.8 ± 8.9	34.1 ± 5.9	13.9 ± 6.9	8.6 ± 1.3	9.6 ± 3.6
		Placebo	placebo	insulin	106	38	60.9 ± 7.8	33.9 ± 5.5	12.6 ± 7.3	8.5 ± 1.3	8.9 ± 2.9
Rosenstock 2007 (NCT00101803)[90]	24	Glitazone	pioglitazone 30 mg daily	none	161	36	52.4 ± 10.3	28.9 ± 5.5	2.2 ± 3.3	8.7 ± 1	10.5 ± 3.1
Kikuchi 2012 (NCT00297063)[91]	28	Glitazone	rosiglitazone 4-8 mg daily	none	159	37.1	55 ± 10.6	24.5 ± 3.7	0.4 ± 0.4	8.9 ± 1.3	10.2 ± 2.4
		Glitazone	pioglitazone 15-45 mg daily	none	159	37.7	56 ± 10.3	24.9 ± 3.5	0.3 ± 0.4	8.8 ± 1.3	10.6 ± 2.6
Henry 2015 (NCT01691755, NCT01691846, NCT01691989)[92]	26	Glitazar	aleglitazar 0.15 mg daily	not specified	293	59.7	54.7 ± 10.3	30.8 ± 5.1	5.7 ± 5.1	8 ± 0.8	8.7 ± 1.9

Ratner 2007 (NCT00242372)[93]	24	Glitazar	tesaglitazar 0.5 mg daily	insulin	192	52.1	57.7 ± NA	35.4 ± 8	11.6 ± NA	8.5 ± 0.7	9.2 ± 3.6
		Placebo	placebo	insulin	200	44	55 ± NA	34.9 ± 6.5	10.5 ± NA	8.4 ± 0.6	8.9 ± 3.3
Umpierrez 2006[94]	26	Glitazone	pioglitazone 30-45 mg daily	metformin	107	44	55.7 ± 9.7	33.8 ± 6.6	5.9 ± 6.1	8.3 ± 0.8	10.2 ± 2.3
Bae 2020 (NCT04013581)[95]	24	Glitazone	pioglitazone 15-30 mg daily	metformin, sulfonylurea and DPP-4 inhibitor	59	44.1	61.9 ± 9	26.2 ± 3.7	13.9 ± 8.4	8 ± 0.8	8.1 ± 2.7
Dailey 2004[96]	24	Glitazone	rosiglitazone 4 mg daily	metformin or sulfonylurea	181	42	57 ± 9	32 ± 5	9 ± 7	8.1 ± 0.9	9.9 ± 2.5
		Placebo	placebo	metformin or sulfonylurea	184	39	57 ± 10	32 ± 5	9 ± 6	8.1 ± 0.8	9.6 ± 2.6
Miyazaki 2001[97]	16	Glitazone	pioglitazone 45 mg daily	sulfonylurea	12	8.3	54 ± 10.4	28.7 ± 3.8	5.8 ± 4.8	8.9 ± 1	10.2 ± 2.9
		Placebo	placebo	sulfonylurea	11	45.5	55 ± 13.3	29.5 ± 4.3	4.7 ± 4.6	7.9 ± 1	8.8 ± 2.4
Goke 2002[98]	24	Glitazone	pioglitazone 45 mg daily	none	129	46.5	58.9 ± 9.1	30.9 ± 5.3	4.8 ± 4.6	9 ± 1.2	10.4 ± 2.5
Rubin 2008 (NCT00095030)[99]	52	Glitazar	muraglitazar 2.5-5 mg daily	metformin	1214	53.5	54.4 ± 9.2	30.8 ± 4.9	NA	8.1 ± 1	9.2 ± 2.5
Kendall 2006[100]	24	Glitazar	muraglitazar 5 mg daily	metformin	587	54.2	55.3 ± 8.6	32 ± 4.6	6 ± 5	8.1 ± 1	NA
		Glitazone	pioglitazone 30 mg daily	metformin	572	51	54.1 ± 9	32 ± 4.6	5.8 ± 5.1	8.1 ± 1.1	NA

Chou 2008[101]	28	Glitazone	rosiglitazone 4-8 mg daily	none	227	40	53.6 ± 10.7	31.3 ± 5.8	2 ± NA	NA	11.8 ± 3.7
Zhang 2005[102]	24	Glitazone	rosiglitazone 4-8 mg daily	none	16	NA	69.5 ± 2.6	24.1 ± 0.6	NA	8.1 ± 0.4	9.6 ± 0.6
Nauck 2016 (NCT01183013)[103]	30	Glitazone	pioglitazone 15-45 mg daily	none	409	46.7	56.6 ± 10.9	32.8 ± 5.5	NA	8.1 ± 0.9	9.3 ± 2.1
Jin 2015 (NCT01106131)[104]	24	Glitazone	lobeglitazone 0.5 mg daily	metformin	128	57	56.3 ± 9.1	25.7 ± 3.5	7.3 ± 5	7.9 ± 0.8	8.6 ± 2.6
		Glitazone	pioglitazone 15 mg daily	metformin	125	49.6	57.5 ± 9.6	25.2 ± 3.1	7.8 ± 5.9	8 ± 0.7	8.7 ± 2.2
Jain 2006[105]	56	Glitazone	pioglitazone 15-45 mg daily	none	251	47	52.1 ± 11.3	32.5 ± 5.8	0.8 ± 1.3	9.2 ± 1.3	10.5 ± 2.9
Matthews 2005[106]	52	Glitazone	pioglitazone 15-45 mg daily	metformin	317	49.2	56 ± 9.2	32.6 ± 5	5.8 ± 5.1	8.7 ± 1	11.8 ± 3.1
Fernandez 2011[107]	16	Glitazar	muraglitazar 5 mg daily	not specified	20	35	50 ± 8.9	33 ± 3.1	3.7 ± 2.2	8.5 ± 1.8	10.2 ± 2.2
		Placebo	placebo	not specified	7	57.1	54 ± 7.9	29.4 ± 3.7	3.1 ± 2.5	9.3 ± 0.7	10.6 ± 2.2
Scherbaum 2002[108]	26	Glitazone	pioglitazone 15-30 mg daily	none	159	41.2	58.8 ± NA	29.5 ± NA	NA	9.2 ± 1.2	12.4 ± 2.8
		Placebo	placebo	none	84	44	59.1 ± NA	29.2 ± NA	NA	8.9 ± 1	11.5 ± 2.5
Buse 2005[109]	24	Glitazar	muraglitazar 2.5-5 mg	none	114	43.5	52.5 ± 10.2	31.3 ± 5.2	2.2 ± 3.4	7.9 ± 1	9.4 ± 3

			daily							
		Placebo	placebo	none	115	54	50.4 ± 10	31.5 ± NA	2.3 ± 3.9	8 ± 1	9 ± 2.3
Hanefeld 2004[110]	52	Glitazone	pioglitazone 15-45 mg daily	sulfonylurea	319	46.4	60 ± 8.8	30.2 ± 4.4	7 ± 5.6	8.8 ± 1	11.8 ± 2.7
Kho 2007[111]	12	Glitazone	pioglitazone 15 mg daily	none	21	47.6	52 ± 11	27.7 ± 2.9	NA	7.3 ± 1.6	8.3 ± 2.8
		Placebo	placebo	none	19	52.6	58 ± 11	27.8 ± 3.5	NA	6.9 ± 2.6	8.6 ± 2.5
Kawamori 1998[112]	12	Glitazone	pioglitazone 30 mg daily	not specified	21	33.4	57.6 ± 8.5	23 ± 1.8	12.5 ± 9.1	8.4 ± 1.4	8.7 ± 2.1
		Placebo	placebo	not specified	9	44.4	60.6 ± 10	22 ± 3	11.9 ± 8.1	8.7 ± 1.3	7.8 ± 1.3
Einhorn 2000[113]	16	Glitazone	pioglitazone 30 mg daily	metformin	168	45.2	55.5 ± 10.3	32.1 ± 5.3	NA	9.9 ± 1.4	14 ± 3.9
		Placebo	placebo	metformin	160	40	55.7 ± 9.9	32.1 ± 5.5	NA	9.8 ± 1.3	14.4 ± 3.8
Kipnes 2001[114]	23	Glitazone	pioglitazone 15-30 mg daily	sulfonylurea	373	40.5	56.6 ± 10	31.9 ± 6.2	NA	9.9 ± 1.4	13.5 ± 3.5
		Placebo	placebo	sulfonylurea	187	41.7	56.9 ± 8.9	32 ± 4.9	NA	9.9 ± 1.4	13.1 ± 3.4
Ohira 2014[115]	26	Glitazone	pioglitazone 15 mg daily	metformin	30	36.7	63.7 ± 7.9	23.6 ± 4.1	NA	8.5 ± 1.1	12.3 ± 4.5
Gupta 2009 (NCT00219440)[116]	16	Glitazone	pioglitazone 30 mg daily	metformin and sulfonylurea	32	54.5	57.2 ± 10	34.9 ± 6.2	NA	6.3 ± 2	NA
Papathanassio 2009[117]	26	Glitazone	pioglitazone 30 mg daily	metformin	14	78.6	62.8 ± 7.2	33.9 ± 7	NA	7.7 ± 0.7	8.7 ± 2.5
Xing 2012[118]	12	Glitazone	pioglitazone 15-45 mg	metformin and/or	44	52.3	52.6 ± 9.4	24 ± 3.5	6.5 ± 4.7	8.4 ± 2	10.3 ± 1.9

			daily	acarbose							
Kim 2005[119]	12	Glitazone	rosiglitazone 4 mg daily	metformin and sulfonylurea	63	66.7	58.8 ± 8.8	23.9 ± 2.5	12 ± 6.4	9.8 ± 1.8	11.9 ± 2.5
		Placebo	placebo	metformin and sulfonylurea	62	62.9	58.1 ± 9.5	24.5 ± 3	10.1 ± 6.3	9.3 ± 1.3	11.5 ± 2.2
Saad 2004[120]	12	Glitazar	ragaglitazar 4 mg daily	none	62	50	53.4 ± NA	31 ± NA	NA	8.5 ± NA	10.8 ± 2.4
		Glitazone	pioglitazone 30 mg daily	none	28	60.7	55 ± NA	31 ± NA	NA	8.5 ± NA	11.9 ± 2.4
		Placebo	placebo	none	30	40	54 ± NA	31 ± NA	NA	8.1 ± NA	11.5 ± 2.3
Sykes 2014 (NCT00500331)[121]	12	Glitazone	pioglitazone 30 mg daily	none	49	50	54.5 ± 9.4	NA	NA	8.1 ± 0.6	NA
Raskin 2000[122]	22	Glitazone	troglitazone 200-600 mg daily	none	85	30.6	57 ± 11.2	29.6 ± 5	5.6 ± 5.6	8.7 ± 1	11.9 ± 2.6
Naka 2011[123]	26	Glitazone	rosiglitazone 8 mg daily	insulin	17	82.4	64.7 ± 7.6	28.8 ± 3.9	20.1 ± 8.3	8.8 ± 1.1	NA
		Placebo	placebo	insulin	17	71.4	67.3 ± 6.4	29 ± 4	17.1 ± 6.2	8.8 ± 1	NA
Patel 1999[124]	12	Glitazone	rosiglitazone 4 mg daily	none	80	31.2	59.7 ± 10	28.4 ± 4.1	5.8 ± NA	9 ± NA	11.7 ± NA
		Placebo	placebo	none	75	30.7	56.8 ± 11.5	28.9 ± 4	4.2 ± NA	9.1 ± NA	11.9 ± NA
Lebovitz 2001[125]	26	Glitazone	rosiglitazone 4-8 mg daily	none	327	34.3	60.5 ± 9.6	29.6 ± 4	5.1 ± 5.9	8.9 ± 1.6	12.4 ± 3.5
		Placebo	placebo	none	158	34.2	59 ± 10.9	29.9 ± 4.1	4.6 ± 4.8	9 ± 1.7	12.7 ± 3.3
Khanolkar 2007[126]	24	Glitazone	rosiglitazone 4 mg daily	metformin	25	40	59 ± NA	34.5 ± 8.8	NA	7.3 ± 0.5	NA

Derosa 2008[127]	26	Glitazone	rosiglitazone 8 mg daily	metformin	56	53.6	55 ± 4	28.6 ± 1.9	3 ± 1	7.8 ± 0.7	8.8 ± 1.1
Ko 2006[128]	52	Glitazone	rosiglitazone 2-8 mg daily	metformin and/or sulfonylurea	56	42.9	56.6 ± 10.1	25.3 ± 3.8	11.8 ± 7.7	10.1 ± 1	12.5 ± 4
Satirapoj 2018 (TCTR20180424002)[129]	24	Glitazone	pioglitazone 15 mg daily	not specified	37	54.1	61.8 ± 11.6	27.3 ± 5.1	NA	9.2 ± 1.8	11.1 ± 4.8
Khaloo 2018 (NCT03125694)[130]	52	Glitazone	pioglitazone 30 mg daily	metformin and sulfonylurea	125	58.5	62.7 ± 8.2	29 ± 4.8	14.3 ± 6.9	9 ± 1.2	10 ± 2.6
Tan 2004[131]	52	Glitazone	pioglitazone 30-45 mg daily	none	91	38	60 ± 8.5	30.2 ± 5.6	4.8 ± 4.7	8.4 ± 0.7	10.7 ± 2
Wilding 2007 (NCT00251940) [132]	24	Glitazar	tesaglitazar 0.5-1 mg daily	sulfonylurea	386	45.1	60.9 ± NA	28.4 ± NA	7.8 ± NA	8 ± 0.8	9.6 ± 1.8
		Placebo	placebo	sulfonylurea	182	45.1	60.2 ± NA	29 ± NA	8.3 ± NA	7.9 ± 0.7	9.4 ± 2
Esteghamati 2015 (NCT01963663)[133]	12	Glitazone	pioglitazone 30 mg daily	none	42	55	51.8 ± 8.4	29.4 ± 3.7	NA	8.1 ± 1.3	9.7 ± 2.9
Yale 2001[134]	24	Glitazone	troglitazone 400 mg daily	metformin and sulfonylurea	101	45	58 ± 9	30.1 ± 5	11.9 ± 8	9.6 ± 1	13.1 ± 3
		Placebo	placebo	metformin and sulfonylurea	99	42	60 ± 9	30 ± 4	10.8 ± 6	9.7 ± 1	12.9 ± 2.8
Gastaldelli 2006[135]	16	Glitazone	pioglitazone 45 mg daily	sulfonylurea	10	NA	53 ± 12.6	30.2 ± 3.5	6 ± 6.3	9 ± 1.3	10 ± 2.5

		Placebo	placebo	sulfonylurea	10	NA	53 ± 12.6	28.6 ± 4.1	4 ± 3.2	7.8 ± 0.9	9.3 ± 1.9
Jung 2003[136]	26	Glitazone	rosiglitazone 4 mg daily	sulfonylurea	14	57.1	60 ± 8	23.3 ± 2.6	9 ± 5	9.6 ± 1.5	11.4 ± 2.1
Erdem 2008[137]	12	Glitazone	pioglitazone 15-45 mg daily	none	21	61.9	54.9 ± 7.8	30.4 ± 4.2	NA	6.3 ± 1.2	7.1 ± 1.3
Fidan 2011[138]	12	Glitazone	rosiglitazone 4-8 mg daily	none	20	45	54.1 ± 9	30.9 ± 4.8	NA	7.9 ± 0.8	8.3 ± 1.7
Jung 2005[139]	26	Glitazone	rosiglitazone 4 mg daily	sulfonylurea	15	57.1	60 ± 8	23.3 ± 2.6	9 ± 5	9.3 ± 0.9	11.4 ± 2.1
Gupta 2012[140]	12	Glitazone	rosiglitazone 4-8 mg daily	treatment naïve or metformin or sulfonylurea	55	45	58.5 ± 9.9	27.5 ± 3.6	NA	8.8 ± 1.6	10.1 ± 3.6
Goke 2007 (NCT00251953) [141]	24	Glitazar	tesaglitazar 0.5-1 mg daily	metformin	390	42.6	58.7 ± 10.5	30.4 ± 5.9	6.2 ± NA	7.7 ± 0.7	9 ± 1.7
		Placebo	placebo	metformin	200	46	60.1 ± 10.2	30.5 ± 6.5	7.8 ± NA	7.8 ± 0.7	8.9 ± 1.7
Pan 2002[142]	12	Glitazone	pioglitazone 30 mg daily	none	141	NA	NA	24.7 ± 2.9	NA	8.5 ± 1.3	8.8 ± 1.3
		Placebo	placebo	none	142	NA	NA	25.4 ± 2.8	NA	8.5 ± 1.1	9.2 ± 1.4
Rosenblatt 2001[143]	16	Glitazone	pioglitazone 30 mg daily	none	101	47	53.8 ± 10	31.5 ± 4.7	NA	10.7 ± 1.8	15.3 ± 3.9
		Placebo	placebo	none	96	43.8	55.2 ± 10	30.7 ± 5	NA	10.4 ± 1.7	15.1 ± 4
Tan 2007[144]	24	Glitazone	rosiglitazone 4-8 mg daily	metformin and/or sulfonylurea	32	34.4	55.4 ± 9.8	25.4 ± 3.6	10.1 ± 5	8.7 ± 0.7	8.7 ± 1.5

Jovanovic 2003[145]	24	Glitazone	pioglitazone 30 mg daily	none	62	50	56.2 ± 12.2	32.1 ± 5.3	6.1 ± 3.9	9.1 ± NA	14.1 ± NA
Taslimi 2013 (IRCT201102275917N1)[146]	12	Glitazone	pioglitazone 15-30 mg daily	none	30	56.7	56 ± 11	28.4 ± 4	3 ± 0.8	8.2 ± 1.8	9.9 ± 3.6
Kirk 1999[147]	12	Glitazone	troglitazone 400 mg daily	none	16	68.8	54.5 ± 9.1	34.1 ± 5.8	5.3 ± 5.6	10 ± NA	11.7 ± NA

Data are presented as mean ± standard deviation. NA: Not available; BMI: Body mass index; HbA1c: Hemoglobin A1c; DPP-4=dipeptidyl peptidase 4.

Section S3 Risk of bias assessment of included studies for each outcome

Table S3 Risk of bias assessment of included studies for HbA1c change

**Study identifier**	**Randomization process**	**Deviations from intended intervention**	**Missing outcome data**	**Measurement of the outcome**	**Selection of the reported result**	**Overall bias**
Davidson 2007	Low	Low	Low	Low	Low	Low
Chou 2012	Some concerns	Low	Some concerns	Low	Low	Some concerns
Truitt 2010	Some concerns	Low	High	Low	Low	High
Goldberg 2005	Low	Some concerns	High	Low	Low	High
John 2002	Low	Some concerns	Low	Low	Low	Some concerns
Bays 2007	Low	Low	Low	Low	Low	Low
Ryang 2022	Low	Some concerns	Low	Low	Low	Some concerns
Hanefeld 2007	Low	Low	Some concerns	Low	Low	Some concerns
Raskin 2001	Low	Low	Low	Low	Low	Low
Herz 2003	Low	Low	Low	Low	Low	Low
Kong 2011	Low	Low	Low	Low	Low	Low
Wolffenbuttel 2000	Low	Low	High	Low	Low	High
Davidson 2006	Low	Low	High	Low	Low	High
Zhu 2003	Low	Low	Some concerns	Low	Low	Some concerns
Stirban 2016	Low	Low	Low	Low	Low	Low
Wallace 2003	Low	Low	Low	Low	Low	Low
Vongthavaravat 2002	Low	Low	Low	Low	Low	Low
Satoh 2003	High	Low	High	Low	Low	High
Kadoglou 2007	Low	Low	Low	Low	Low	Low
Bertrand 2010	Low	Low	Low	Low	Low	Low
Jia 2021	Low	Low	Low	Low	Low	Low
Wei 2013	Low	Low	Low	Low	Low	Low
Raskin 2004	Low	Low	High	Low	Low	High
Rosenstock 2002	Low	Low	Low	Low	Some concerns	Some concerns
Esteghamati 2014	Some concerns	Some concerns	Low	Low	Low	Some concerns
Raman 2023	Low	Some concerns	Low	Low	Low	Some concerns
Kato 2009	Low	Low	Low	Low	Low	Low
Takihata 2013	Low	Some concerns	Low	Low	Low	Some concerns

Jameshorani 2017	Some concerns	Low	Low	Low	Low	Some concerns
Yoneda 2021	Low	Low	Low	Low	Low	Low
Erem 2014	Low	Low	Low	Low	Low	Low
Hamann 2007	Low	Low	Low	Low	Low	Low
Perriello 2006	Low	Low	Low	Low	Low	Low
Strowig 2002	Low	Low	Low	Low	Low	Low
Yamanouchi 2005	Low	Low	Low	Low	Low	Low
Fujitaka 2011	Low	Low	Low	Low	Low	Low
Nagasaka 2003	Low	Low	Some concerns	Low	Low	Some concerns
Xu 2015	Low	Some concerns	Low	Low	Low	Some concerns
Yoon 2011	Low	Low	Some concerns	Low	Low	Some concerns
Baksi 2003	Low	Low	Low	Low	Low	Low
Kim 2016	Low	Low	Low	Low	Low	Low
Bolli 2008	Low	Low	High	Low	High	High
Reynolds 2007	Low	Low	Low	Low	Low	Low
Virtanen 2003	High	Low	Low	Low	Low	High
Derosa 2009	Some concerns	Low	Low	Low	Low	Some concerns
Miyazaki 2002	Some concerns	Low	Low	Low	Low	Some concerns
Iwamoto 1995	Some concerns	High	Some concerns	Low	Low	High
Rajagopalan 2015	Low	Low	Low	Low	Low	Low
Fonseca 2000	Low	Low	Some concerns	Low	Low	Some concerns
Derosa 2010	Low	Low	Low	Low	Low	Low
Pavo 2003	Low	Low	Low	Low	Low	Low
Genovese 2013	Low	Low	Low	Low	Low	Low
Miyazaki 2001	Low	Low	Low	Low	Low	Low
Carey 2002	Low	Low	Low	Low	Low	Low
Goldstein 2006	Some concerns	Low	Low	Low	Low	Some concerns
Henry 2009	Low	Low	Low	Low	Low	Low
Triwatana 2022	Some concerns	Low	Low	Low	Low	Some concerns
Derosa 2006	Low	Low	Low	Low	Low	Low
Hartemann-Heurtier 2009	Low	Low	Low	Low	Low	Low
Sathyanarayana 2011	Some concerns	Low	Low	Low	Low	Some concerns
Bhagat 2022	Low	Low	Low	Low	Low	Low
Defronzo 2010	Low	Low	Low	Low	Low	Low
Xiao 2015	Low	Some concerns	Low	Low	Low	Some concerns

Naka 2012	High	Low	Low	Low	Low	High
Teramoto 2007	Low	Low	Low	Low	Low	Low
Sourij 2006	Low	Low	Low	Low	Low	Low
Weissman 2005	Low	Low	Low	Low	Low	Low
Iwamoto 1996	Some concerns	Some concerns	Low	Low	Low	Some concerns
Erande 2013	Low	Low	Low	Low	Low	Low
Liu 2013	Low	Low	Low	Low	Low	Low
Ji 2021	Low	Low	Low	Low	Low	Low
Jones 2012	Low	Low	Some concerns	Low	Low	Some concerns
Bergenstal 2010	Low	Low	Low	Low	Low	Low
Kim 2014	Low	Low	Low	Low	Low	Low
Rubin 2009	Low	Low	Some concerns	Low	Low	Some concerns
Kaku 2015	Low	Low	Low	Low	Low	Low
Rosenstock 2002	Low	Low	Low	Low	Low	Low
Kim 2020	Low	Low	Low	Low	Low	Low
Schernthaner 2004	Low	Low	Low	Low	Low	Low
Perez 2009	Low	Low	Some concerns	Low	Low	Some concerns
Perez 2002	Low	Low	High	Low	Low	High
Scott 2007	Low	Low	Low	Low	Low	Low
Rodrigues 2022	Low	Low	Low	Low	Low	Low
Henriksen 2011	Some concerns	Low	Low	Low	Low	Some concerns
Rosenstock 2007	Low	Low	Low	Low	Low	Low
Kikuchi 2012	Low	Low	Low	Low	Low	Low
Henry 2015	Low	Low	Low	Low	Low	Low
Ratner 2007	Low	Low	High	Low	Low	High
Umpierrez 2006	Some concerns	Low	Low	Low	Low	Some concerns
Bae 2020	Low	Low	Low	Low	Low	Low
Dailey 2004	Low	Low	High	Low	Low	High
Miyazaki 2001	High	Low	Low	Low	Low	High
Goke 2002	Low	Low	Low	Low	Low	Low
Rubin 2008	Low	Low	Low	Low	Low	Low
Kendall 2006	Low	Low	Low	Low	Low	Low
Chou 2008	Low	Low	Low	Low	Low	Low
Nauck 2016	Low	Low	Low	Low	Low	Low
Jin 2015	Low	Low	Low	Low	Low	Low
Jain 2006	Low	Low	Low	Low	Low	Low
Matthews 2005	Low	Low	Low	Low	Low	Low
Fernandez 2011	Some concerns	Low	Low	Low	Low	Some concerns
Scherbaum 2002	Low	Low	Low	Low	Low	Low
Hanefeld 2004	Low	Low	Low	Low	Low	Low
Kawamori 1998	Low	Low	Low	Low	Low	Low

Einhorn 2000	Low	Low	Some concerns	Low	Low	Some concerns
Kipnes 2001	Low	Low	Low	Low	Low	Low
Ohira 2014	Low	Low	Low	Low	Low	Low
Gupta 2009	Low	Some concerns	Low	Low	Low	Some concerns
Papathanassio 2009	Low	Low	Low	Low	Low	Low
Xing 2012	Low	Some concerns	Low	Low	Low	Some concerns
Kim 2005	Low	Some concerns	Low	Low	Low	Some concerns
Sykes 2014	Low	Low	Low	Low	Low	Low
Raskin 2000	Low	Some concerns	High	Low	Low	High
Naka 2011	Low	Some concerns	Low	Low	Low	Some concerns
Patel 1999	Low	Some concerns	Low	Low	High	High
Lebovitz 2001	Low	Low	Low	Low	Low	Low
Khanolkar 2007	Low	Low	Low	Low	Low	Low
Derosa 2008	Low	Low	Low	Low	Low	Low
Ko 2006	Low	Low	Low	Low	Low	Low
Satirapoj 2018	Low	Low	Low	Low	Low	Low
Khaloo 2018	Some concerns	Low	Low	Low	Low	Some concerns
Tan 2004	Low	Low	High	Low	Low	High
Wilding 2007	Low	Low	Low	Low	Low	Low
Esteghamati 2015	Low	Some concerns	Low	Low	Low	Some concerns
Yale 2001	Low	Low	Low	Low	Low	Low
Gastaldelli 2006	Low	Low	Low	Low	Low	Low
Erdem 2008	Low	Some concerns	Low	Low	Low	Some concerns
Fidan 2011	Low	Low	Low	Low	Low	Low
Jung 2005	Low	Some concerns	Low	Low	Low	Some concerns
Gupta 2012	Low	Some concerns	Low	Low	Low	Some concerns
Goke 2007	Low	Low	Low	Low	Low	Low
Rosenblatt 2001	Low	Low	Low	Low	Low	Low
Tan 2007	Low	Low	Low	Low	Low	Low
Jovanovic 2003	Low	Low	High	Low	Low	High
Taslimi 2013	Low	Low	Low	Low	Low	Low
Kirk 1999	Low	Low	Low	Low	Low	Low
Lü 2011	Low	Low	Low	Low	Low	Low
Yang 2014	Low	Low	Low	Low	Low	Low
Hao 2005	Low	Low	Low	Low	Low	Low
Wang 2004	Low	Low	Low	Low	Low	Low

Zhang 2005	Low	Low	Low	Some concerns	Low	Some concerns
Jung 2003	Low	Low	Low	Low	Low	Low
Kho 2007	Low	Low	Low	Low	Low	Low
Pan 2002	Low	Low	Low	Low	Low	Low

Table S4 Risk of bias assessment of included studies for FPG change

Study identifier	Randomization process	Deviations from intended intervention	Missing outcome data	Measurement of the outcome	Selection of the reported result	Overall bias
Davidson 2007	Low	Low	Low	Low	Low	Low
Chou 2012	Some concerns	Low	Some concerns	Low	Low	Some concerns
Truitt 2010	Some concerns	Low	High	Low	Low	High
Goldberg 2005	Low	Some concerns	High	Low	Low	High
Bays 2007	Low	Low	Low	Low	Low	Low
Hanefeld 2007	Low	Low	Some concerns	Low	Low	Some concerns
Raskin 2001	Low	Low	Low	Low	Low	Low
Kong 2011	Low	Low	Low	Low	Low	Low
Davidson 2006	Low	Low	High	Low	Low	High
Zhu 2003	Low	Low	Some concerns	Low	Low	Some concerns
Stirban 2016	Low	Low	Low	Low	Low	Low
Wallace 2003	Low	Low	Low	Low	Low	Low
Vongthavaravat 2002	Low	Low	Low	Low	Low	Low
Satoh 2003	High	Low	High	Low	Low	High
Kadoglou 2007	Low	Low	Low	Low	Low	Low
Bertrand 2010	Low	Low	Low	Low	Low	Low
Jia 2021	Low	Low	Low	Low	Low	Low
Wei 2013	Low	Low	Low	Low	Low	Low
Raskin 2004	Low	Low	High	Low	Low	High
Veleba 2015	Low	Low	Low	Low	Low	Low
Esteghamati 2014	Some concerns	Some concerns	Low	Low	Low	Some concerns
Raman 2023	Low	Some concerns	Low	Low	Low	Some concerns
Kato 2009	Low	Low	Low	Low	Low	Low
Takihata 2013	Low	Some concerns	Low	Low	Low	Some concerns
Jameshorani 2017	Some concerns	Low	Low	Low	Low	Some concerns
Yoneda 2021	Low	Low	Low	Low	Low	Low
Erem 2014	Low	Low	Low	Low	Low	Low
Hamann 2007	Low	Low	Low	Low	Low	Low
Perriello 2006	Low	Low	Low	Low	Low	Low
Strowig 2002	Low	Low	Low	Low	Low	Low
Yamanouchi 2005	Low	Low	Low	Low	Low	Low
Fujitaka 2011	Low	Low	Low	Low	Low	Low
Xu 2015	Low	Some concerns	Low	Low	Low	Some concerns

Yoon 2011	Low	Low	Some concerns	Low	Low	Some concerns
Baksi 2003	Low	Low	Low	Low	Low	Low
Kim 2016	Low	Low	Low	Low	Low	Low
Bolli 2008	Low	Low	High	Low	High	High
Virtanen 2003	High	Low	Low	Low	Low	High
Derosa 2009	Some concerns	Low	Low	Low	Low	Some concerns
Miyazaki 2002	Some concerns	Low	Low	Low	Low	Some concerns
Iwamoto 1995	Some concerns	High	Some concerns	Low	Low	High
Rajagopalan 2015	Low	Low	Low	Low	Low	Low
Fonseca 2000	Low	Low	Some concerns	Low	Low	Some concerns
Derosa 2010	Low	Low	Low	Low	Low	Low
Pavo 2003	Low	Low	Low	Low	Low	Low
Genovese 2013	Low	Low	Low	Low	Low	Low
Miyazaki 2001	Low	Low	Low	Low	Low	Low
Carey 2002	Low	Low	Low	Low	Low	Low
Goldstein 2006	Some concerns	Low	Low	Low	Low	Some concerns
Henry 2009	Low	Low	Low	Low	Low	Low
Triwatana 2022	Some concerns	Low	Low	Low	Low	Some concerns
Derosa 2006	Low	Low	Low	Low	Low	Low
Hartemann-Heurtier 2009	Low	Low	Low	Low	Low	Low
Sathyanarayana 2011	Some concerns	Low	Low	Low	Low	Some concerns
Bhagat 2022	Low	Low	Low	Low	Low	Low
Defronzo 2010	Low	Low	Low	Low	Low	Low
Xiao 2015	Low	Some concerns	Low	Low	Low	Some concerns
Naka 2012	High	Low	Low	Low	Low	High
Teramoto 2007	Low	Low	Low	Low	Low	Low
Sourij 2006	Low	Low	Low	Low	Low	Low
Weissman 2005	Low	Low	Low	Low	Low	Low
Iwamoto 1996	Some concerns	Some concerns	Low	Low	Low	Some concerns
Erande 2013	Low	Low	Low	Low	Low	Low
Liu 2013	Low	Low	Low	Low	Low	Low
Ji 2021	Low	Low	Low	Low	Low	Low
Bergenstal 2010	Low	Low	Low	Low	Low	Low
Kim 2014	Low	Low	Low	Low	Low	Low
Rubin 2009	Low	Low	Some concerns	Low	Low	Some concerns
Kaku 2015	Low	Low	Low	Low	Low	Low

Rosenstock 2002	Low	Low	Low	Low	Low	Low
Schernthaner 2004	Low	Low	Low	Low	Low	Low
Perez 2009	Low	Low	Some concerns	Low	Low	Some concerns
Perez 2002	Low	Low	High	Low	Low	High
Scott 2007	Low	Low	Low	Low	Low	Low
Rodrigues 2022	Low	Low	Low	Low	Low	Low
Henriksen 2011	Some concerns	Low	Low	Low	Low	Some concerns
Rosenstock 2007	Low	Low	Low	Low	Low	Low
Kikuchi 2012	Low	Low	Low	Low	Low	Low
Henry 2015	Low	Low	Low	Low	Low	Low
Ratner 2007	Low	Low	High	Low	Low	High
Umpierrez 2006	Some concerns	Low	Low	Low	Low	Some concerns
Bae 2020	Low	Low	Low	Low	Low	Low
Dailey 2004	Low	Low	High	Low	Low	High
Miyazaki 2001	High	Low	Low	Low	Low	High
Goke 2002	Low	Low	Low	Low	Low	Low
Rubin 2008	Low	Low	Low	Low	Low	Low
Chou 2008	Low	Low	Low	Low	Low	Low
Nauck 2016	Low	Low	Low	Low	Low	Low
Jin 2015	Low	Low	Low	Low	Low	Low
Matthews 2005	Low	Low	Low	Low	Low	Low
Fernandez 2011	Some concerns	Low	Low	Low	Low	Some concerns
Scherbaum 2002	Low	Low	Low	Low	Low	Low
Buse 2005	Low	Low	High	Low	Low	High
Hanefeld 2004	Low	Low	Low	Low	Low	Low
Kawamori 1998	Low	Low	Low	Low	Low	Low
Einhorn 2000	Low	Low	Some concerns	Low	Low	Some concerns
Kipnes 2001	Low	Low	Low	Low	Low	Low
Ohira 2014	Low	Low	Low	Low	Low	Low
Papathanassio 2009	Low	Low	Low	Low	Low	Low
Xing 2012	Low	Some concerns	Low	Low	Low	Some concerns
Kim 2005	Low	Some concerns	Low	Low	Low	Some concerns
Saad 2004	Low	Some concerns	Some concerns	Low	Low	Some concerns
Sykes 2014	Low	Low	Low	Low	Low	Low
Raskin 2000	Low	Some concerns	High	Low	Low	High
Patel 1999	Low	Some concerns	Low	Low	High	High
Lebovitz 2001	Low	Low	Low	Low	Low	Low
Derosa 2008	Low	Low	Low	Low	Low	Low

Ko 2006	Low	Low	Low	Low	Low	Low
Satirapoj 2018	Low	Low	Low	Low	Low	Low
Khaloo 2018	Some concerns	Low	Low	Low	Low	Some concerns
Tan 2004	Low	Low	High	Low	Low	High
Wilding 2007	Low	Low	Low	Low	Low	Low
Esteghamati 2015	Low	Some concerns	Low	Low	Low	Some concerns
Yale 2001	Low	Low	Low	Low	Low	Low
Gastaldelli 2006	Low	Low	Low	Low	Low	Low
Erdem 2008	Low	Some concerns	Low	Low	Low	Some concerns
Fidan 2011	Low	Low	Low	Low	Low	Low
Jung 2005	Low	Some concerns	Low	Low	Low	Some concerns
Gupta 2012	Low	Some concerns	Low	Low	Low	Some concerns
Goke 2007	Low	Low	Low	Low	Low	Low
Rosenblatt 2001	Low	Low	Low	Low	Low	Low
Tan 2007	Low	Low	Low	Low	Low	Low
Jovanovic 2003	Low	Low	High	Low	Low	High
Taslimi 2013	Low	Low	Low	Low	Low	Low
Kirk 1999	Low	Low	Low	Low	Low	Low
Lü 2011	Low	Low	Low	Low	Low	Low
Yang 2014	Low	Low	Low	Low	Low	Low
Hao 2005	Low	Low	Low	Low	Low	Low
Wang 2004	Low	Low	Low	Low	Low	Low
Zhang 2005	Low	Low	Low	Some concerns	Low	Some concerns
Jung 2003	Low	Low	Low	Low	Low	Low
Kho 2007	Low	Low	Low	Low	Low	Low
Pan 2002	Low	Low	Low	Low	Low	Low

Fig. S1 Summary of domain-specific risk of bias assessments

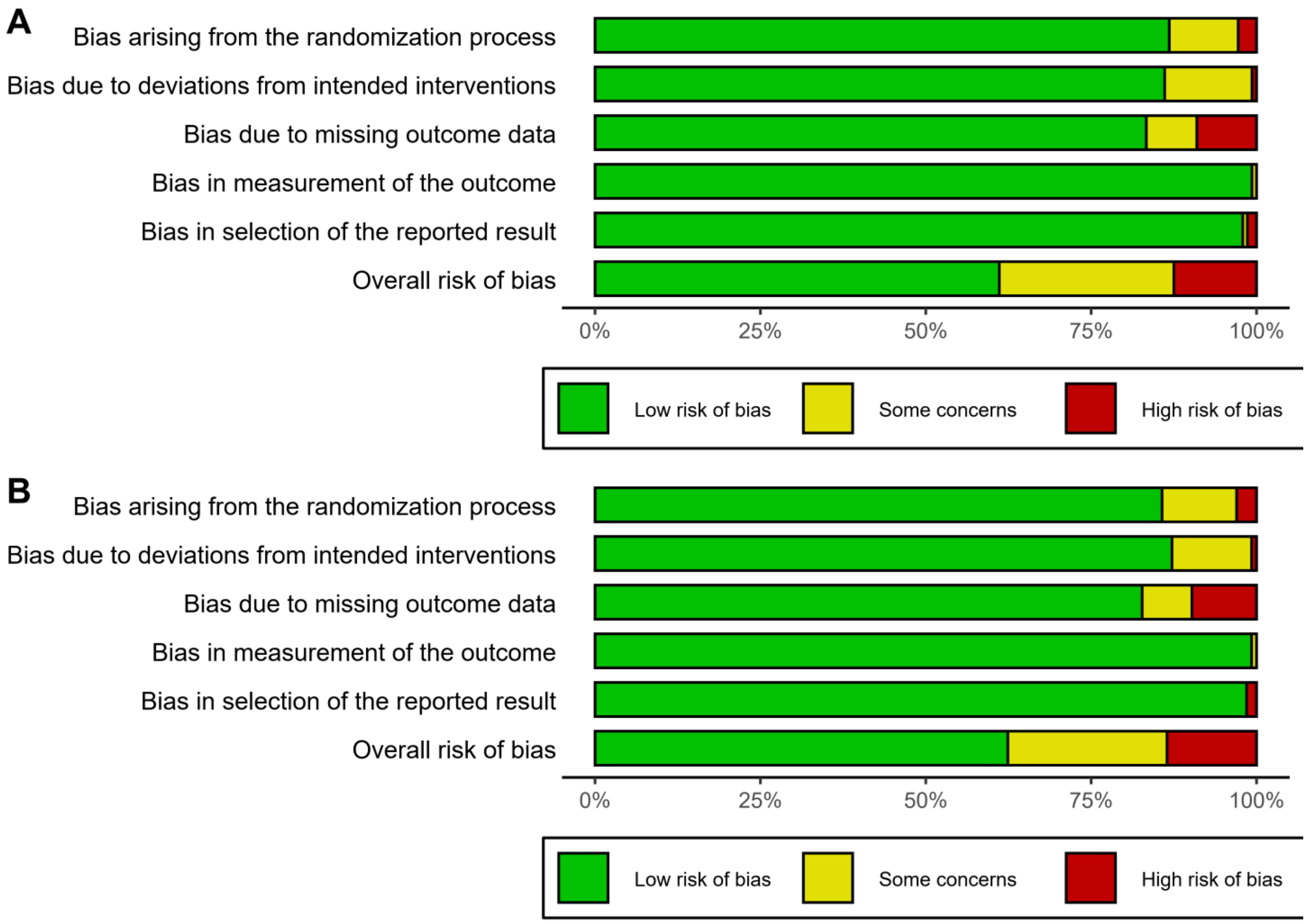

(A) Domain-specific risk of bias summary for studies reporting HbA1c. (B) Domain specific risk of bias summary for studies reporting FPG

Section S4 Other main results

Fig. S2 Funnel plot of HbA1c change.

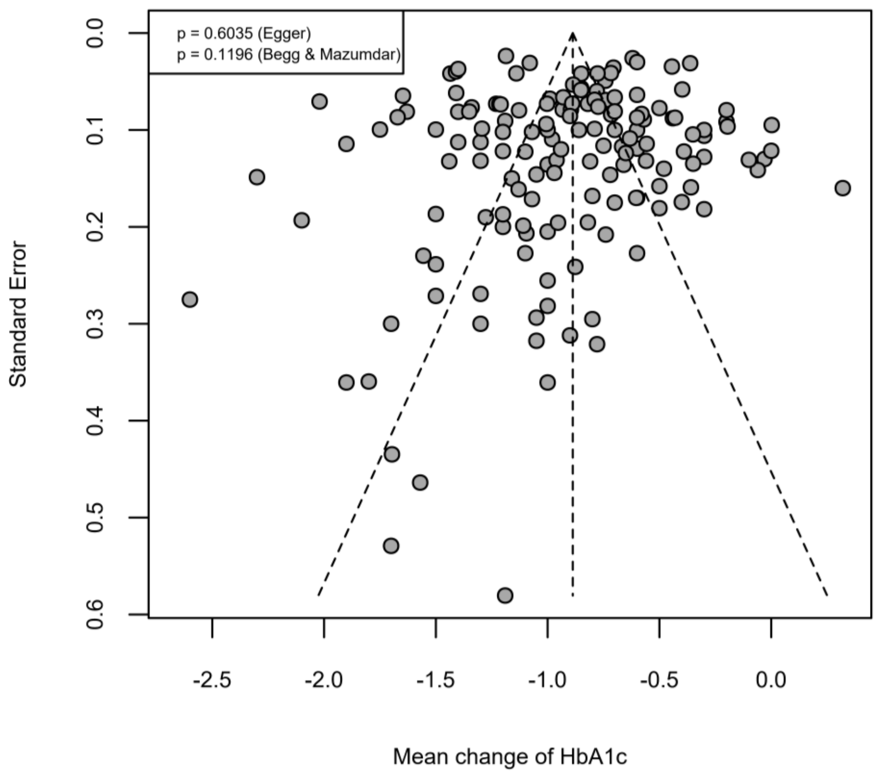

Fig. S3 Funnel plot of FPG change.

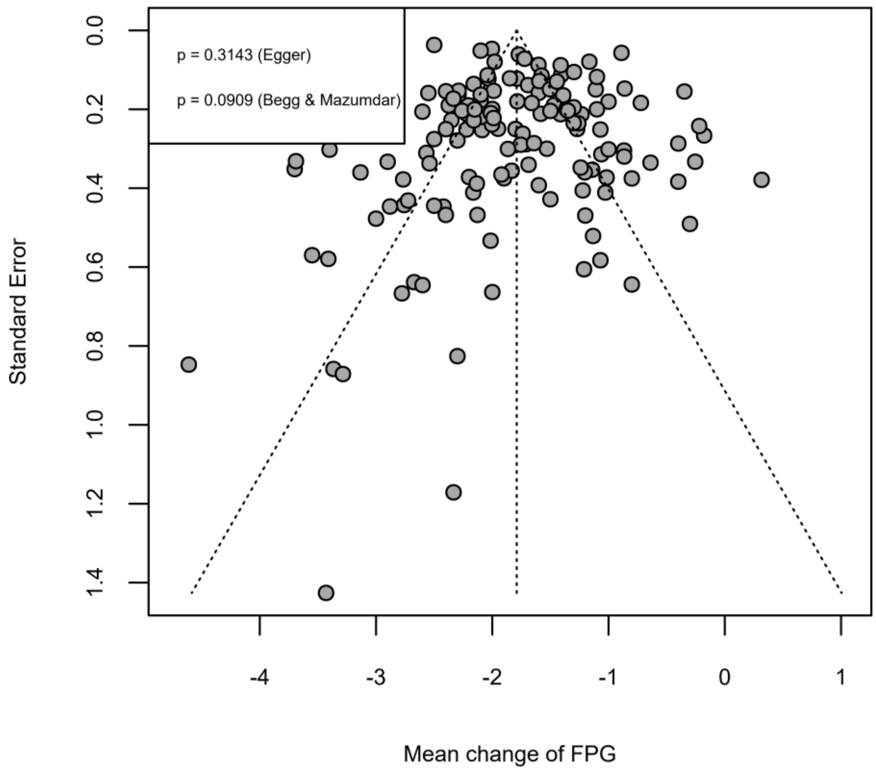

FPG: fasting plasma glucose.

Fig. S4 Forest plot of HbA1c change

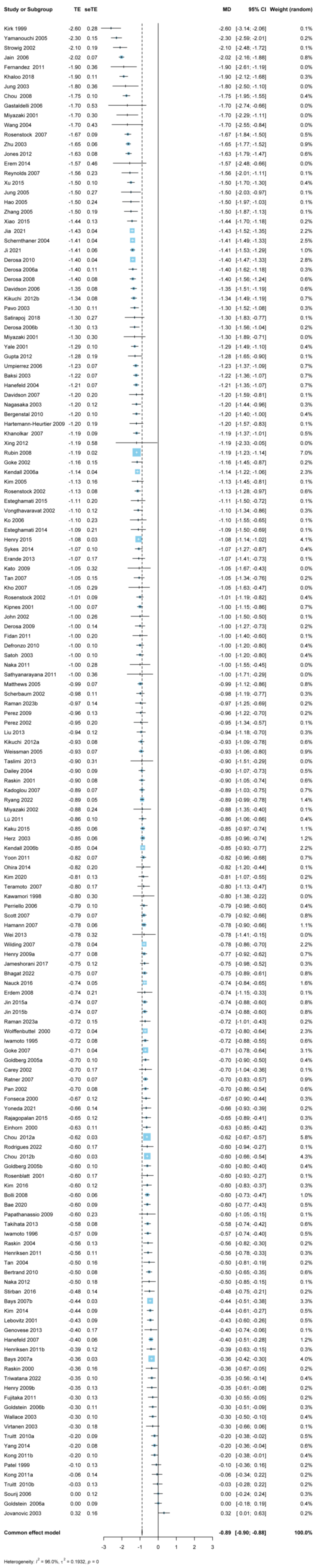

TE: treatment effect; seTE: standard error of treatment effect; CI: confidence interval; MD: mean difference.

Fig. S5 Forest plot of FPG change

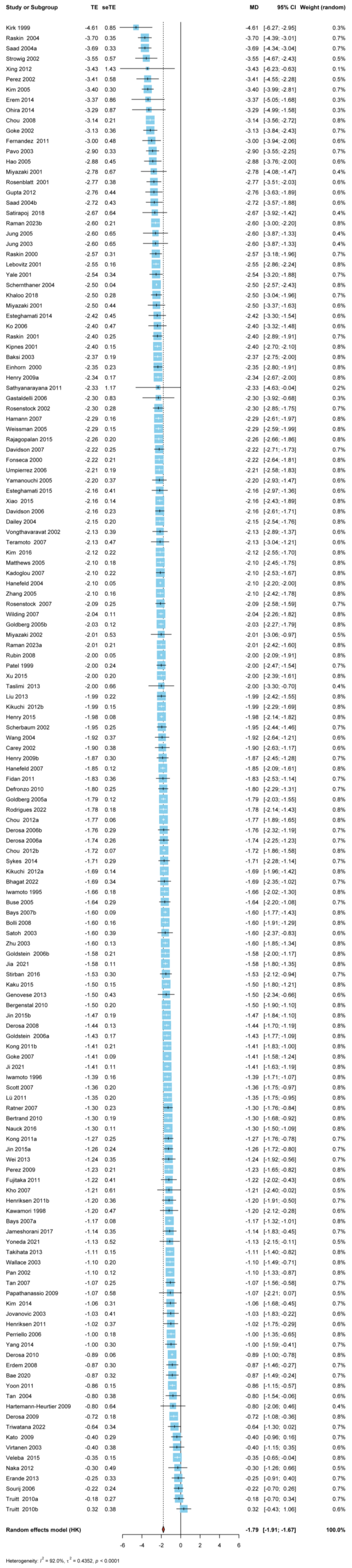

TE: treatment effect; seTE: standard error of treatment effect; CI: confidence interval; MD: mean difference; FPG: fasting plasma glucose.

Table S5 Univariable meta-regression analysis of factors associated with treatment response (unadjusted)

	HbA1c, %					FPG, mmol/L				
	β	95% CI	*p*	adj.*P*	N	*β*	95% CI	*p*	adj.*P*	N
Baseline HbA1c/FPG, %/ (mmol/L)	0.2713	[0.2005, 0.3421]	<.0001	<.0001	154	0.3054	[0.2571, 0.3537]	<.0001	<.0001	144
Proportion of female participants, %	0.0095	[0.0038, 0.0152]	0.0011	0.0039	148	0.0105	[0.0011, 0.0198]	0.0279	0.06	138
Age, year	-0.0315	[-0.0497, -0.0133]	0.0007	0.0026	149	-0.0181	[-0.0484, 0.0121]	0.2405	0.294	140
Baseline BMI, kg/m2	-0.0073	[-0.028, 0.0134]	0.4901	0.6739	149	0.0248	[-0.0068, 0.0564]	0.124	0.1949	141
Baseline SBP, mmHg	0.0003	[-0.0141, 0.0148]	0.9633	0.9633	53	0.0014	[-0.0251, 0.0279]	0.9178	0.9178	49
Baseline DBP, mmHg	0.0304	[0.0061, 0.0547]	0.0143	0.0315	52	0.0299	[-0.017, 0.0768]	0.2115	0.2908	48
Baseline HOMA-IR	0.0519	[-0.006, 0.1099]	0.0792	0.1244	57	0.0816	[0.0067, 0.1565]	0.0327	0.06	51
Baseline LDL-C, mmol/L	-0.0486	[-0.2364, 0.1391]	0.6116	0.7475	113	0.4078	[0.0935, 0.722]	0.011	0.0302	106
Baseline HDL-C, mmol/L	-1.0598	[-1.6706, -0.449]	0.0007	0.0026	124	-2.4327	[-3.45, -1.4154]	<.0001	<.0001	116
Baseline TG, mmol/L	-0.0266	[-0.1816, 0.1284]	0.7366	0.8103	118	0.4748	[0.2193, 0.7303]	0.0003	0.001	111
Baseline fasting insulin, uIU/mL	0.0231	[0.0028, 0.0435]	0.0258	0.0473	77	0.0171	[-0.0164, 0.0507]	0.3174	0.3879	72

P values were adjusted by the Benjamini-Hochberg method. BMI: body mass index; FPG: fasting plasma glucose; SBP: systolic blood pressure; DBP: diastolic blood pressure; HOMA-IR: homeostatic model assessment of insulin resistance; LDL-C: low-density lipoprotein cholesterol; HDL-C: high-density lipoprotein cholesterol, TG: triglyceride.

Table S6 Univariable meta-regression analysis of factors associated with treatment response(multi-variable adjusted)

	*Reduction in HbA1c, %					‡Reduction in FPG, mmol/L				
	β	95% CI	*p*	adj.*P*	N	*β*	95% CI	*p*	adj.*P*	N
Proportion of female participants, %	0.0062	[0.0011, 0.0112]	0.0177	0.0885	141	0.0084	[0.0009, 0.016]	0.0287	0.1585	131
Age, year	-0.0378	[-0.056, -0.0196]	0	0.0005	142	-0.0241	[-0.0507, 0.0025]	0.0761	0.1902	133
Baseline BMI, kg/m2	-0.0009	[-0.0203, 0.0186]	0.9315	0.9631	140	-0.001	[-0.0305, 0.0285]	0.9477	0.9477	133
Baseline SBP, mmHg	-0.0081	[-0.0232, 0.0069]	0.289	0.4128	52	-0.0135	[-0.0385, 0.0114]	0.2886	0.481	48
Baseline DBP, mmHg	0.0246	[-0.0012, 0.0503]	0.0612	0.1531	51	0.0251	[-0.0193, 0.0694]	0.2683	0.481	47
Baseline HOMA-IR	0.0083	[-0.0434, 0.0601]	0.7527	0.9409	52	-0.0368	[-0.1125, 0.039]	0.3417	0.4881	46
Baseline LDL-C, mmol/L	-0.0001	[-0.0047, 0.0044]	0.9631	0.9631	108	0.0027	[-0.0048, 0.0102]	0.482	0.6025	102
Baseline HDL-C, mmol/L	-0.0113	[-0.0256, 0.003]	0.1227	0.2455	116	-0.0241	[-0.046, -0.0021]	0.0317	0.1585	109
Baseline TG, mmol/L	-0.0011	[-0.0026, 0.0005]	0.1828	0.3047	110	-0.0005	[-0.003, 0.002]	0.7096	0.7885	104
Baseline fasting insulin, uIU⁄mL	0.0186	[0.0015, 0.0356]	0.0326	0.1088	73	0.0303	[-0.0013, 0.0618]	0.0605	0.1902	68

* represents adjustment for baseline HbA1c, duration of the trial, background therapy and type of the drug; ‡ represent adjustment for baseline FPG, duration of the trial, background therapy and type of the drug. *P* values were adjusted by the Benjamini-Hochberg method. BMI: body mass index; FPG: fasting plasma glucose; SBP: systolic blood pressure; DBP: diastolic blood pressure; HOMA-IR: homeostatic model assessment of insulin resistance; LDL-C: low-density lipoprotein cholesterol; HDL-C: high-density lipoprotein cholesterol, TG: triglyceride.

Section S5 Sensitivity analysis

Fig. S6 Statistically significant factors associated with HbA1c and FPG reduction in glitazone and glitazar subgroups

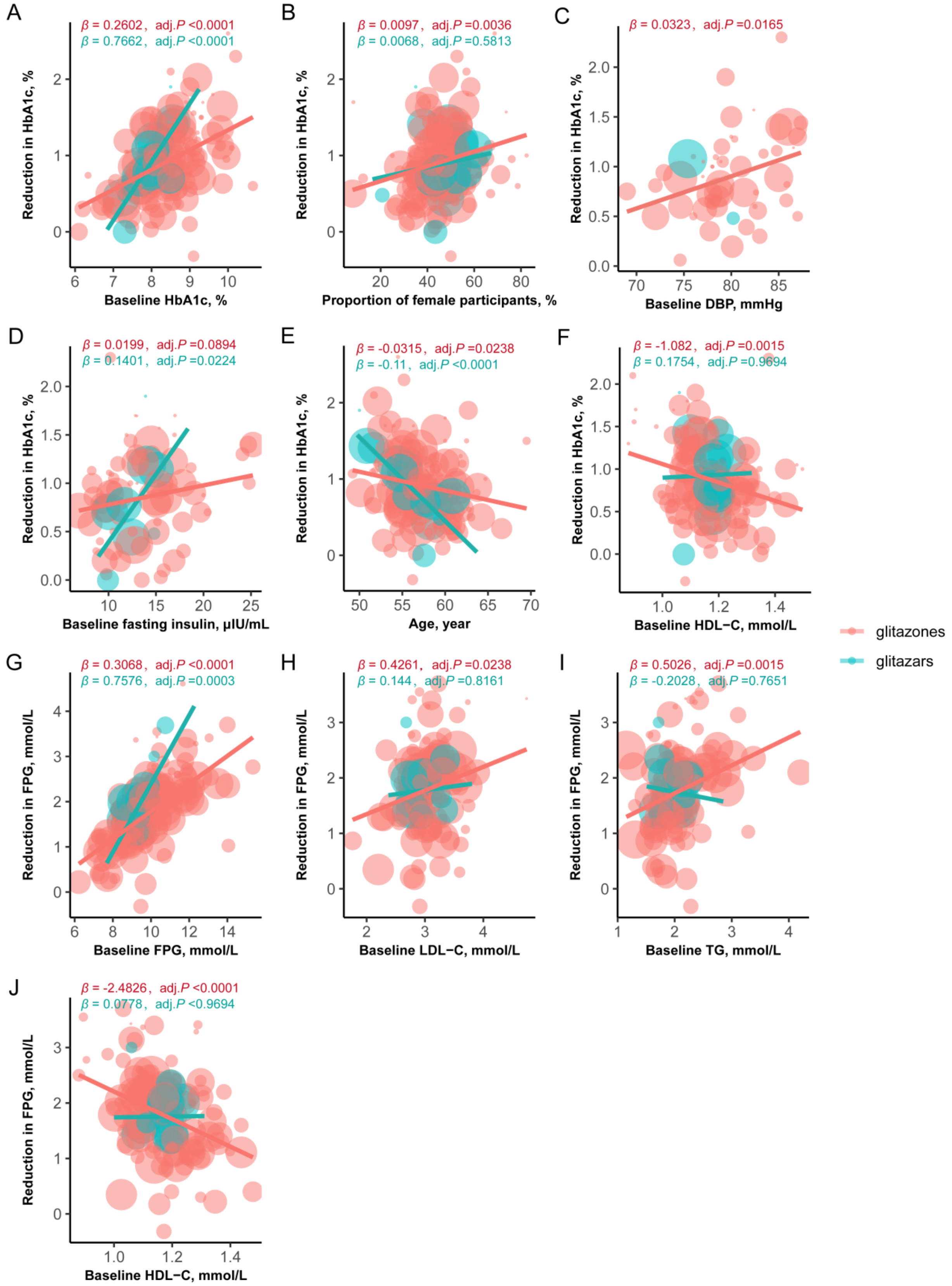

Association between HbA1c decline and baseline HbA1c (A), female proportion (B), age (C)， baseline DBP (D) and LDL-C (E). Association between FPG decline and baseline FPG (F), baseline LDL-C (G), baseline HDL-C (H) and baseline TG (I). FPG: fasting plasma glucose; LDL-C: low-density lipoprotein cholesterol; HDL-C: high-density lipoprotein cholesterol, TG: triglyceride. *P* values were adjusted by the Benjamini-Hochberg method.

Table S7 Sensitivity analysis of univariable meta regression in glitazone subgroup

	*Reduction in HbA1c, %					‡Reduction in FPG, mmol/L				
	β	95% CI	*p*	adj.*P*	N	*β*	95% CI	*p*	adj.*P*	N
Proportion of female participants, %	0.0071	[0.0016, 0.0127]	0.0115	0.0495	134	0.0063	[-0.0011, 0.0138]	0.0954	0.4769	123
Age, year	-0.0155	[-0.0336, 0.0026]	0.094	0.1566	135	-0.0043	[-0.028, 0.0194]	0.7235	0.8592	125
Baseline BMI, kg/m2	-0.0043	[-0.0241, 0.0155]	0.671	0.671	135	-0.0152	[-0.0414, 0.011]	0.2568	0.5749	127
Baseline SBP, mmHg	-0.0041	[-0.0189, 0.0106]	0.5814	0.6609	51	-0.0135	[-0.0324, 0.0054]	0.1607	0.5356	47
Baseline DBP, mmHg	0.0272	[0.0043, 0.0502]	0.0202	0.0495	50	0.0153	[-0.0163, 0.0468]	0.3431	0.5749	46
Baseline HOMA-IR	0.0238	[-0.0253, 0.073]	0.342	0.4886	51	0.0043	[-0.0509, 0.0594]	0.8789	0.8789	46
Baseline LDL-C, mmol/L	-0.0503	[-0.2358, 0.1352]	0.5948	0.6609	101	0.0384	[-0.2228, 0.2995]	0.7733	0.8592	95
Baseline HDL-C, mmol/L	-0.6938	[-1.279, -0.1086]	0.0201	0.0505	111	-1.227	[-2.0317, -0.4222]	0.0028	0.0281	104
Baseline TG, mmol/L	-0.1917	[-0.3348, -0.0486]	0.0087	0.0505	105	-0.1036	[-0.3185, 0.1114]	0.345	0.5749	99
Baseline fasting insulin, uIU/mL	0.0176	[-0.0001, 0.0353]	0.0515	0.1031	69	0.0057	[-0.0213, 0.0328]	0.6773	0.8592	64

* represents adjustment for baseline HbA1c; ‡ represent adjustment for baseline FPG. *P* values were adjusted by the Benjamini-Hochberg method. BMI: body mass index; FPG: fasting plasma glucose; SBP: systolic blood pressure; DBP: diastolic blood pressure; HOMA-IR: homeostatic model assessment of insulin resistance; LDL-C: low-density lipoprotein cholesterol; HDL-C: high-density lipoprotein cholesterol, TG: triglyceride.

Table S8 Sensitivity analysis of univariable meta regression in glitazar subgroup

	*Reduction in HbA1c, %					‡Reduction in FPG, mmol/L				
	β	95% CI	*p*	adj.*P*	N	*β*	95% CI	*p*	adj.*P*	N
Proportion of female participants, %	-0.0014	[-0.0138, 0.0111]	0.8316	0.8316	13	0.015	[-0.0028, 0.0327]	0.0985	0.3446	14
Age, year	-0.0596	[-0.0983, -0.0208]	0.0026	0.0207	13	-0.0044	[-0.0711, 0.0623]	0.8975	0.9783	14
Baseline BMI, kg/m2	-0.0335	[-0.0806, 0.0136]	0.1632	0.2611	13	0.0382	[-0.0385, 0.1149]	0.3291	0.69	14
Baseline LDL-C, mmol/L	-0.7222	[-1.4374, -0.007]	0.0478	0.0956	11	-0.4193	[-1.5649, 0.7263]	0.4732	0.69	11
Baseline HDL-C, mmol/L	0.4453	[-2.2566, 3.1472]	0.7467	0.8316	12	1.5529	[-2.8857, 5.9915]	0.4929	0.69	12
Baseline TG, mmol/L	0.5395	[0.0982, 0.9808]	0.0166	0.0663	12	-0.0145	[-1.0598, 1.0308]	0.9783	0.9783	12
Baseline fasting insulin, uIU/mL	0.066	[0.0028, 0.1293]	0.0407	0.0956	8	0.0935	[0.0088, 0.1782]	0.0306	0.214	8

* represents adjustment for baseline HbA1c; ‡ represent adjustment for baseline FPG. *P* values were adjusted by the Benjamini-Hochberg method. BMI: body mass index; FPG: fasting plasma glucose; LDL-C: low-density lipoprotein cholesterol; HDL-C: high-density lipoprotein cholesterol, TG: triglyceride.

Table S9 Sensitivity analysis of univariable meta regression in pioglitazone subgroup

	*Reduction in HbA1c, %					‡Reduction in FPG, mmol/L				
	β	95% CI	*p*	adj.*P*	N	*β*	95% CI	*p*	adj.*P*	N
Proportion of female participants, %	0.0037	[-0.0043, 0.0116]	0.3658	0.732	82	0.0032	[-0.007, 0.0134]	0.5397	0.6747	76
Age, year	-0.022	[-0.0464, 0.0034]	0.0901	0.301	82	-0.017	[-0.0501, 0.0157]	0.3062	0.6125	77
Baseline BMI, kg/m2	-0.019	[-0.0468, 0.0081]	0.1671	0.418	83	-0.042	[-0.0786, -0.0058]	0.0232	0.1161	79
Baseline SBP, mmHg	-0.003	[-0.0201, 0.015]	0.7792	0.97	34	-0.015	[-0.0374, 0.0069]	0.1776	0.444	33
Baseline DBP, mmHg	0.0262	[-0.0002, 0.0525]	0.0519	0.259	33	0.0144	[-0.0253, 0.0542]	0.4767	0.6747	32
Baseline HOMA-IR	0.0036	[-0.0708, 0.078]	0.9241	0.97	35	0	[-0.0857, 0.0857]	0.9995	0.9995	32
Baseline LDL-C, mmol/L	-0.005	[-0.2625, 0.2526]	0.97	0.97	61	0.1274	[-0.212, 0.4668]	0.462	0.6747	59
Baseline HDL-C, mmol/L	-0.267	[-1.1373, 0.6034]	0.5478	0.783	67	-1.273	[-2.3561, -0.1901]	0.0212	0.1161	64
Baseline TG, mg/dL	-0.365	[-0.5508, -0.1781]	0.0001	0.001	62	-0.212	[-0.5031, 0.0797]	0.1544	0.444	60
Baseline fasting insulin, uIU⁄mL	0.0091	[-0.0153, 0.0335]	0.4628	0.771	46	0.0065	[-0.0305, 0.0436]	0.7296	0.8107	43

* represents adjustment for baseline HbA1c; ‡ represent adjustment for baseline FPG. *P* values were adjusted by the Benjamini-Hochberg method. BMI: body mass index; FPG: fasting plasma glucose; LDL-C: low-density lipoprotein cholesterol; HDL-C: high-density lipoprotein cholesterol, TG: triglyceride.

Table S10 Sensitivity analysis of univariable meta regression in rosiglitazone subgroup

	*Reduction in HbA1c, %					‡Reduction in FPG, mmol/L				
	β	95% CI	*p*	adj.*P*	N	*β*	95% CI	*p*	adj.*P*	N
Proportion of female participants, %	0.0113	[0.0029, 0.0197]	0.0082	0.082	37	0.0052	[-0.0055, 0.0159]	0.3427	0.8569	34
Age, year	-0.002	[-0.0317, 0.0282]	0.908	0.968	38	0.0056	[-0.0242, 0.0354]	0.7135	0.9578	35
Baseline BMI, kg/m2	-0.027	[-0.0683, 0.0141]	0.197	0.394	38	0.0318	[-0.0036, 0.0673]	0.0784	0.7837	35
Baseline SBP, mmHg	-0.0007	[-0.0364, 0.035]	0.9676	0.968	11	-0.003	[-0.0771, 0.071]	0.9363	0.9578	9
Baseline DBP, mmHg	0.037	[-0.0011, 0.0752]	0.0572	0.191	11	-0.009	[-0.0691, 0.0514]	0.7733	0.9578	9
Baseline HOMA-IR	0.0261	[-0.0545, 0.1068]	0.5252	0.75	12	0.0055	[-0.0694, 0.0805]	0.8847	0.9578	11
Baseline LDL-C, mmol/L	-0.127	[-0.446, 0.1925]	0.4365	0.728	31	-0.28	[-0.6749, 0.1155]	0.1654	0.827	28
Baseline HDL-C, mmol/L	-0.231	[-1.1986, 0.737]	0.6402	0.8	33	-0.695	[-2.1096, 0.7203]	0.3359	0.8569	30
Baseline TG, mg/dL	-0.159	[-0.3719, 0.0546]	0.1448	0.362	32	-0.063	[-0.3439, 0.2174]	0.6586	0.9578	29
Baseline fasting insulin, uIU⁄mL	0.0293	[0.0004, 0.0581]	0.0466	0.191	18	0.0009	[-0.0325, 0.0343]	0.9578	0.9578	17

* represents adjustment for baseline HbA1c; ‡ represent adjustment for baseline FPG. *P* values were adjusted by the Benjamini-Hochberg method. BMI: body mass index; FPG: fasting plasma glucose; LDL-C: low-density lipoprotein cholesterol; HDL-C: high-density lipoprotein cholesterol, TG: triglyceride.

Table S11 Variance inflation factors in the multivariate regression model

**Variable**	**VIF in HbA1c model**	**VIF in FPG model**
Baseline HbA1c/FPG, %/mmol/L	1.4720	1.4885
Proportion of female participants, %	1.1748	1.1260
Age, year	1.1765	1.1767
Baseline BMI, kg/m^2	1.2374	1.1633
Baseline HDL-C, mmol/L	1.3637	1.3023
Baseline TG, mmol/L	1.3818	1.6046
Background Therapy	1.3271	1.1546
Duration of the trial, week	1.0635	1.0579

VIF: variance inflation factor; FPG: fasting plasma glucose; BMI: body mass index; HDL-C: high-density lipoprotein cholesterol, TG: triglyceride.

Table S12 Sensitivity analysis of univariable meta regression by excluding trials with high risk of bias

	*Reduction in HbA1c, %					‡Reduction in FPG, mmol/L				
	β	95% CI	*p*	adj.*P*	N	*β*	95% CI	*p*	adj.*P*	N
Proportion of female participants, %	0.0076	[0.0024, 0.0129]	0.0044	0.0221	127	0.0079	[0.0007, 0.0151]	0.032	0.16	117
Age, year	-0.0186	[-0.0344, -0.0028]	0.0213	0.0434	128	-0.005	[-0.0268, 0.0167]	0.6494	0.7444	119
Baseline BMI, kg/m2	0.0055	[-0.0134, 0.0243]	0.5692	0.6322	128	0.01	[-0.016, 0.036]	0.4506	0.7444	121
Baseline SBP, mmHg	-0.0035	[-0.0179, 0.0109]	0.6322	0.6322	47	-0.0091	[-0.0293, 0.0112]	0.3806	0.7444	43
Baseline DBP, mmHg	0.0278	[0.0041, 0.0516]	0.0217	0.0434	46	0.015	[-0.0201, 0.0502]	0.4019	0.7444	42
Baseline HOMA-IR	0.0352	[-0.0158, 0.0861]	0.1764	0.252	53	0.0017	[-0.062, 0.0654]	0.9583	0.9583	47
Baseline LDL-C, mmol/L	-0.0748	[-0.2479, 0.0983]	0.3971	0.4964	96	0.0598	[-0.184, 0.3037]	0.6305	0.7444	90
Baseline HDL-C, mmol/L	-0.7456	[-1.346, -0.1452]	0.0149	0.0434	106	-1.1615	[-1.998, -0.3249]	0.0065	0.0651	99
Baseline TG, mmol/L	-0.1507	[-0.2979, -0.0035]	0.0448	0.0746	100	-0.0916	[-0.3097, 0.1266]	0.4106	0.7444	94
Baseline fasting insulin, uIU/mL	0.0246	[0.0081, 0.0411]	0.0036	0.0221	65	0.0056	[-0.0202, 0.0314]	0.67	0.7444	60

* represents adjustment for baseline HbA1c; ‡ represent adjustment for baseline FPG. *P* values were adjusted by the Benjamini-Hochberg method. BMI: body mass index; FPG: fasting plasma glucose; SBP: systolic blood pressure; DBP: diastolic blood pressure; HOMA-IR: homeostatic model assessment of insulin resistance; LDL-C: low-density lipoprotein cholesterol; HDL-C: high-density lipoprotein cholesterol, TG: triglyceride.

Table S13 Sensitivity analysis of univariable meta regression by excluding trials with participants fewer than 15 per treatment arm

	*Reduction in HbA1c, %					‡Reduction in FPG, mmol/L				
	β	95% CI	*p*	adj.*P*	N	*β*	95% CI	*p*	adj.*P*	N
Proportion of female participants, %	0.0089	[0.0035, 0.0143]	0.0012	0.0124	142	0.0084	[0.0011, 0.0157]	0.0246	0.123	132
Age, year	-0.0227	[-0.0391, -0.0062]	0.0069	0.0346	142	-0.0082	[-0.0306, 0.0142]	0.4739	0.8592	133
Baseline BMI, kg/m2	-0.0045	[-0.023, 0.014]	0.6313	0.6313	141	-0.0034	[-0.0283, 0.0214]	0.7874	0.8592	134
Baseline SBP, mmHg	-0.0046	[-0.0188, 0.0096]	0.5222	0.5802	52	-0.0155	[-0.0352, 0.0041]	0.1219	0.4062	48
Baseline DBP, mmHg	0.0257	[0.0027, 0.0488]	0.0284	0.06	51	0.0105	[-0.0237, 0.0448]	0.5463	0.8592	47
Baseline HOMA-IR	0.0248	[-0.0216, 0.0711]	0.2953	0.3831	55	0.0053	[-0.0532, 0.0638]	0.8592	0.8592	49
Baseline LDL-C, mmol/L	-0.0937	[-0.2733, 0.0859]	0.3065	0.3831	108	0.0393	[-0.2139, 0.2926]	0.7608	0.8592	102
Baseline HDL-C, mmol/L	-0.5862	[-1.1683, -0.0042]	0.0484	0.0806	118	-1.1482	[-1.9514, -0.345]	0.0051	0.0508	111
Baseline TG, mmol/L	-0.1751	[-0.3196, -0.0305]	0.0176	0.0587	112	-0.0973	[-0.3106, 0.1159]	0.3708	0.8592	106
Baseline fasting insulin, uIU∕mL	0.0187	[0.0018, 0.0355]	0.03	0.06	72	0.0032	[-0.0225, 0.0289]	0.8069	0.8592	67

* represents adjustment for baseline HbA1c; ‡ represent adjustment for baseline FPG. *P* values were adjusted by the Benjamini-Hochberg method. BMI: body mass index; FPG: fasting plasma glucose; SBP: systolic blood pressure; DBP: diastolic blood pressure; HOMA-IR: homeostatic model assessment of insulin resistance; LDL-C: low-density lipoprotein cholesterol; HDL-C: high-density lipoprotein cholesterol, TG: triglyceride.

Table S14 Sensitivity analysis of univariable meta regression by excluding trials with imputed standard deviation

	*Reduction in HbA1c, %					‡Reduction in FPG, mmol/L				
	β	95% CI	*p*	adj.*P*	N	*β*	95% CI	*p*	adj.*P*	N
Proportion of female participants, %	0.0071	[0.001, 0.0131]	0.0213	0.071	97	0.0084	[0, 0.0167]	0.0487	0.1458	97
Age, year	-0.013	[-0.0319, 0.0051]	0.1554	0.259	98	-0.032	[-0.0604, -0.0037]	0.0268	0.1339	97
Baseline BMI, kg/m2	-0.007	[-0.0283, 0.0144]	0.5252	0.657	99	-0.007	[-0.0367, 0.0222]	0.6281	0.7989	98
Baseline SBP, mmHg	-0.018	[-0.0329, -0.004]	0.0126	0.065	31	-0.023	[-0.0458, 0.0008]	0.0583	0.1458	36
Baseline DBP, mmHg	0.0024	[-0.03, 0.0348]	0.8843	0.939	30	0.0068	[-0.0454, 0.0591]	0.7971	0.8857	35
Baseline HOMA-IR	-0.047	[-0.1115, 0.0172]	0.1513	0.259	34	-0.026	[-0.1252, 0.0727]	0.6029	0.7989	34
Baseline LDL-C, mmol/L	-0.118	[-0.3135, 0.0771]	0.2355	0.337	78	0.1	[-0.1847, 0.3846]	0.4913	0.7989	78
Baseline HDL-C, mmol/L	-0.936	[-1.6753, -0.1969]	0.0131	0.065	83	-1.229	[-2.2074, -0.2496]	0.0139	0.1339	82
Baseline TG, mg/dL	-0.146	[-0.3025, 0.0103]	0.0672	0.168	77	-0.055	[-0.2827, 0.1735]	0.6391	0.7989	77
Baseline fasting insulin, uIU⁄mL	0.0012	[-0.0286, 0.0309]	0.9391	0.939	42	-0.001	[-0.0433, 0.0404]	0.946	0.946	44

* represents adjustment for baseline HbA1c; ‡ represent adjustment for baseline FPG. *P* values were adjusted by the Benjamini-Hochberg method. BMI: body mass index; FPG: fasting plasma glucose; SBP: systolic blood pressure; DBP: diastolic blood pressure; HOMA-IR: homeostatic model assessment of insulin resistance; LDL-C: low-density lipoprotein cholesterol; HDL-C: high-density lipoprotein cholesterol, TG: triglyceride.

Table S15 Sensitivity analysis of univariable meta regression by excluding trials systematically recruiting patients with certain complications

	*Reduction in HbA1c, %					‡Reduction in FPG, mmol/L				
	β	95% CI	p	adj.P	N	β	95% CI	p	adj.P	N
Proportion of female participants, %	0.0066	[0.0012, 0.012]	0.0161	0.05	136	0.0085	[0.0009, 0.0162]	0.0291	0.1455	126
Age, year	-0.023	[-0.0402, -0.0053]	0.0106	0.05	138	-0.015	[-0.0387, 0.0094]	0.2328	0.582	129
Baseline BMI, kg/m2	-0.007	[-0.0256, 0.0118]	0.4708	0.471	137	-0.007	[-0.0325, 0.0186]	0.5932	0.7415	130
Baseline SBP, mmHg	-0.007	[-0.0226, 0.0084]	0.3699	0.411	43	-0.018	[-0.0405, 0.0036]	0.1018	0.3394	39
Baseline DBP, mmHg	0.0287	[0.0029, 0.0546]	0.0294	0.05	42	0.0153	[-0.025, 0.0557]	0.4568	0.7415	38
Baseline HOMA-IR	0.0391	[-0.0125, 0.0906]	0.1376	0.197	51	0.0054	[-0.0588, 0.0695]	0.8699	0.8699	45
Baseline LDL-C, mmol/L	-0.107	[-0.2883, 0.0745]	0.2479	0.31	102	0.0769	[-0.1879, 0.3417]	0.5694	0.7415	96
Baseline HDL-C, mmol/L	-0.768	[-1.3881, -0.1486]	0.0151	0.05	114	-1.104	[-1.9859, -0.2216]	0.0142	0.142	107
Baseline TG, mg/dL	-0.182	[-0.3345, -0.0287]	0.0199	0.05	108	-0.098	[-0.3293, 0.1325]	0.4037	0.7415	102
Baseline fasting insulin, uIU⁄mL	0.0193	[0.0019, 0.0368]	0.0298	0.05	70	0.0034	[-0.0239, 0.0306]	0.8086	0.8699	65

* represents adjustment for baseline HbA1c; ‡ represent adjustment for baseline FPG. *P* values were adjusted by the Benjamini-Hochberg method. BMI: body mass index; FPG: fasting plasma glucose; SBP: systolic blood pressure; DBP: diastolic blood pressure; HOMA-IR: homeostatic model assessment of insulin resistance; LDL-C: low-density lipoprotein cholesterol; HDL-C: high-density lipoprotein cholesterol, TG: triglyceride.

Table S16 Sensitivity analysis of univariable meta regression in monotherapy subgroup

	*Reduction in HbA1c, %					‡Reduction in FPG, mmol/L				
	β	95% CI	*p*	adj.*P*	N	*β*	95% CI	*p*	adj.*P*	N
Proportion of female participants, %	0.0128	[0.0035, 0.022]	0.0067	0.067	65	0.0094	[-0.003, 0.0218]	0.1378	0.5309	63
Age, year	-0.036	[-0.0641, -0.0073]	0.0137	0.068	64	-0.003	[-0.0385, 0.0333]	0.8875	0.9618	62
Baseline BMI, kg/m2	-0.012	[-0.0472, 0.0234]	0.5091	0.819	63	0.0172	[-0.0257, 0.0601]	0.4313	0.8233	64
Baseline SBP, mmHg	0.0039	[-0.021, 0.0287]	0.7618	0.952	20	-0.006	[-0.0382, 0.026]	0.7086	0.8858	19
Baseline DBP, mmHg	0.0308	[-0.0181, 0.0796]	0.2171	0.434	20	0.0232	[-0.0433, 0.0898]	0.494	0.8233	19
Baseline HOMA-IR	-0.055	[-0.1344, 0.0242]	0.1733	0.433	27	0.0529	[-0.0419, 0.1478]	0.2741	0.6853	27
Baseline LDL-C, mmol/L	-0.003	[-0.4205, 0.4145]	0.9888	0.989	46	0.3366	[-0.1321, 0.8053]	0.1593	0.5309	48
Baseline HDL-C, mmol/L	0.2597	[-0.6442, 1.1636]	0.5733	0.819	52	-1.489	[-2.636, -0.3429]	0.0109	0.1089	53
Baseline TG, mg/dL	-0.265	[-0.5099, -0.0194]	0.0344	0.115	48	-0.082	[-0.4122, 0.2484]	0.6269	0.8858	49
Baseline fasting insulin, uIU⁄mL	-0.001	[-0.0345, 0.032]	0.9411	0.989	33	0.0012	[-0.0461, 0.0484]	0.9618	0.9618	33

* represents adjustment for baseline HbA1c; ‡ represent adjustment for baseline FPG. *P* values were adjusted by the Benjamini-Hochberg method. BMI: body mass index; FPG: fasting plasma glucose; SBP: systolic blood pressure; DBP: diastolic blood pressure; HOMA-IR: homeostatic model assessment of insulin resistance; LDL-C: low-density lipoprotein cholesterol; HDL-C: high-density lipoprotein cholesterol, TG: triglyceride.

Table S17 Sensitivity analysis of univariable meta regression in add-on therapy subgroup

	*Reduction in HbA1c, %					‡Reduction in FPG, mmol/L				
	β	95% CI	p	adj.P	N	β	95% CI	p	adj.P	N
Proportion of female participants, %	0.0046	[-0.0006, 0.0098]	0.0817	0.143	82	0.0061	[-0.0025, 0.0147]	0.1653	0.4134	74
Age, year	-0.023	[-0.0411, -0.0055]	0.0104	0.026	84	-0.026	[-0.056, 0.0031]	0.0798	0.3784	77
Baseline BMI, kg/m2	-0.001	[-0.0197, 0.0175]	0.91	0.91	85	-0.016	[-0.0469, 0.0145]	0.3011	0.6021	77
Baseline SBP, mmHg	-0.01	[-0.0265, 0.0062]	0.2234	0.319	33	-0.023	[-0.0465, 0]	0.0498	0.3784	30
Baseline DBP, mmHg	0.0226	[-0.0032, 0.0483]	0.0855	0.143	32	0.0084	[-0.0322, 0.049]	0.6847	0.8559	29
Baseline HOMA-IR	0.0696	[0.0198, 0.1194]	0.0062	0.021	30	0.0035	[-0.0651, 0.0721]	0.9204	0.9204	24
Baseline LDL-C, mmol/L	0.0177	[-0.1617, 0.1971]	0.8466	0.91	66	-0.02	[-0.3565, 0.3164]	0.9069	0.9204	58
Baseline HDL-C, mmol/L	-1.361	[-1.9905, -0.7315]	<0.0001	0.0004	71	-0.927	[-2.075, 0.221]	0.1135	0.3784	63
Baseline TG, mg/dL	-0.069	[-0.2233, 0.085]	0.3794	0.474	69	-0.085	[-0.3709, 0.2001]	0.5577	0.7968	62
Baseline fasting insulin, uIU/mL	0.0342	[0.0191, 0.0492]	<0.0001	<0.0001	44	0.0087	[-0.0194, 0.0368]	0.5447	0.7968	39

* represents adjustment for baseline HbA1c; ‡ represent adjustment for baseline FPG. *P* values were adjusted by the Benjamini-Hochberg method. BMI: body mass index; FPG: fasting plasma glucose; SBP: systolic blood pressure; DBP: diastolic blood pressure; HOMA-IR: homeostatic model assessment of insulin resistance; LDL-C: low-density lipoprotein cholesterol; HDL-C: high-density lipoprotein cholesterol, TG: triglyceride.

Table S18 Sensitivity analysis of placebo-corrected univariable meta regression (unadjusted)

	Reduction in HbA1c, %					Reduction in FPG, mmol/L				
	β	95% CI	*p*	adj.*P*	N	*β*	95% CI	*p*	adj.*P*	N
Baseline HbA1c/FPG, %/ (mmol/L)	-0.2314	[-0.3067, -0.1561]	<.0001	<.0001	59	-0.3327	[-0.4223, -0.2431]	<.0001	<.0001	59
Proportion of female participants, %	-0.0077	[-0.0142, -0.0013]	0.0192	0.0352	56	-0.0256	[-0.0407, -0.0104]	0.001	0.0031	55
Age, year	0.0141	[-0.0115, 0.0396]	0.2802	0.3424	57	0.0726	[0.0132, 0.1321]	0.0167	0.0262	57
Baseline BMI, kg/m2	-0.0044	[-0.0324, 0.0237]	0.7608	0.7608	57	-0.0698	[-0.1376, -0.0019]	0.0439	0.0604	58
Baseline SBP, mmHg	-0.0313	[-0.06, -0.0025]	0.0329	0.0517	12	0.0193	[-0.0482, 0.0869]	0.5747	0.5747	12
Baseline DBP, mmHg	-0.0533	[-0.097, -0.0095]	0.0169	0.0352	12	-0.0336	[-0.1313, 0.064]	0.4996	0.5495	12
Baseline HOMA-IR	-0.039	[-0.1345, 0.0565]	0.4237	0.466	15	-0.268	[-0.4466, -0.0894]	0.0033	0.0072	15
Baseline LDL-C, mg/dL	-0.3678	[-0.6653, -0.0703]	0.0154	0.0352	44	-1.1559	[-1.7492, -0.5626]	0.0001	0.0007	44
Baseline HDL-C, mg/dL	1.1927	[0.3103, 2.0752]	0.0081	0.0296	49	2.7027	[0.655, 4.7504]	0.0097	0.0178	48

P values were adjusted by the Benjamini-Hochberg method. BMI: body mass index; FPG: fasting plasma glucose; SBP: systolic blood pressure; DBP: diastolic blood pressure; HOMA-IR: homeostatic model assessment of insulin resistance; LDL-C: low-density lipoprotein cholesterol; HDL-C: high-density lipoprotein cholesterol, TG: triglyceride.

Table S19 Sensitivity analysis of placebo-corrected univariable meta regression (adjusted for baseline HbA1c/FPG)

	*Reduction in HbA1c, %					‡Reduction in FPG, mmol/L				
	β	95% CI	*p*	adj.*P*	N	*β*	95% CI	*p*	adj.*P*	N
Proportion of female participants, %	-0.001	[-0.0079, 0.0054]	0.7077	0.885	56	-0.01	[-0.0235, 0.0042]	0.1705	0.3084	55
Age, year	-0.001	[-0.0242, 0.022]	0.9255	0.941	57	0.0302	[-0.0176, 0.078]	0.2161	0.3087	57
Baseline BMI, kg/m2	-0.0009	[-0.0249, 0.0231]	0.941	0.941	57	-0.037	[-0.0926, 0.0179]	0.1851	0.3084	58
Baseline SBP, mmHg	-0.021	[-0.0499, 0.0084]	0.1626	0.518	12	0.0254	[-0.025, 0.0758]	0.3236	0.4045	12
Baseline DBP, mmHg	-0.032	[-0.0853, 0.0218]	0.2447	0.518	12	0.0251	[-0.0605, 0.1108]	0.5653	0.6281	12
Baseline HOMA-IR	0.0206	[-0.0448, 0.0859]	0.5375	0.768	15	-0.162	[-0.3354, 0.0121]	0.0682	0.3084	15
Baseline LDL-C, mmol/L	-0.124	[-0.4111, 0.1629]	0.3968	0.661	44	-0.379	[-0.8964, 0.1376]	0.1503	0.3084	44
Baseline HDL-C, mmol/L	0.6169	[-0.2082, 1.442]	0.1428	0.518	49	1.3623	[-0.324, 3.0486]	0.1133	0.3084	48

* represents adjustment for baseline HbA1c; ‡ represent adjustment for baseline FPG. *P* values were adjusted by the Benjamini-Hochberg method. BMI: body mass index; FPG: fasting plasma glucose; SBP: systolic blood pressure; DBP: diastolic blood pressure; HOMA-IR: homeostatic model assessment of insulin resistance; LDL-C: low-density lipoprotein cholesterol; HDL-C: high-density lipoprotein cholesterol, TG: triglyceride.

Table S20 Meta regression of factors associated with placebo response

	Reduction in HbA1c, %					Reduction in FPG, mmol/L				
	β	95% CI	*p*	adj.*P*	N	*β*	95% CI	*p*	adj.*P*	N
Baseline HbA1c/FPG, %/ (mmol/L)	-0.0064	[-0.2185, 0.2056]	0.9525	0.9533	59	-0.0608	[-0.3329, 0.2113]	0.6614	0.9802	59
Proportion of female participants, %	-0.0038	[-0.0203, 0.0127]	0.6544	0.9533	56	-0.0043	[-0.0378, 0.0292]	0.8032	0.9802	55
Age, year	-0.0335	[-0.0928, 0.0258]	0.2683	0.9533	57	0.0258	[-0.0989, 0.1505]	0.685	0.9802	57
Baseline BMI, kg/m2	0.0188	[-0.07, 0.1076]	0.6779	0.9533	57	-0.0244	[-0.2199, 0.171]	0.8065	0.9802	58
Baseline SBP, mmHg	0.0047	[-0.0784, 0.0878]	0.9114	0.9533	12	0.016	[-0.1266, 0.1585]	0.826	0.9802	12
Baseline DBP, mmHg	-0.0169	[-0.1547, 0.1209]	0.8103	0.9533	12	-0.0085	[-0.2127, 0.1957]	0.9353	0.9802	12
Baseline HOMA-IR	-0.0204	[-0.3702, 0.3293]	0.9088	0.9533	15	-0.1237	[-0.953, 0.7056]	0.77	0.9802	15
Baseline LDL-C, mg/dL	-0.631	[-1.4243, 0.1623]	0.119	0.9533	44	-0.1587	[-1.3913, 1.0738]	0.8007	0.9802	44
Baseline HDL-C, mg/dL	-0.6982	[-2.7689, 1.3725]	0.5087	0.9533	49	0.0573	[-4.4637, 4.5782]	0.9802	0.9802	48
Baseline TG, mg/dL	-0.2374	[-0.6556, 0.1807]	0.2658	0.9533	46	-0.0365	[-0.6726, 0.5996]	0.9104	0.9802	45
Baseline fasting insulin, uIU/mL	-0.0025	[-0.0846, 0.0797]	0.9533	0.9533	31	-0.0222	[-0.2076, 0.1632]	0.8143	0.9802	30

P values were adjusted by the Benjamini-Hochberg method. BMI: body mass index; FPG: fasting plasma glucose; SBP: systolic blood pressure; DBP: diastolic blood pressure; HOMA-IR: homeostatic model assessment of insulin resistance; LDL-C: low-density lipoprotein cholesterol; HDL-C: high-density lipoprotein cholesterol, TG: triglyceride.

Table S21 Sensitivity analysis of multivariate meta regression including baseline diastolic blood pressure as covariate

	Reduction in HbA1c, %				Reduction in FPG, mmol/L			
	β	95% CI	*p*	N	*β*	95% CI	*p*	N
Baseline HbA1c/FPG	0.1646	[0.015, 0.3141]	0.031	41	0.2839	[0.1337, 0.4341]	0.0002	38
Proportion of female participants, %	0.0115	[0.0031, 0.0199]	0.0075	41	0.0092	[-0.0042, 0.0227]	0.1781	38
Age, year	-0.0149	[-0.0484, 0.0186]	0.3844	41	-0.009	[-0.0608, 0.0428]	0.7333	38
Baseline BMI, kg/m2	-0.0184	[-0.0564, 0.0196]	0.3431	41	-0.0371	[-0.0989, 0.0247]	0.2396	38
Baseline HDL-C, mmol/L	-0.0179	[-0.0434, 0.0075]	0.1674	41	-0.0632	[-0.0997, -0.0266]	0.0007	38
Baseline TG, mmol/L	0.0003	[-0.0022, 0.0029]	0.7939	41	0.0031	[-0.0011, 0.0074]	0.1499	38
Baseline DBP, mg/dL	0.027	[0, 0.054]	0.05	41	0.0086	[-0.0303, 0.0474]	0.666	38
Duration of the trial, week								
short (<24 weeks)	*Ref*				*Ref*			
middle (>=24 weeks and <48 weeks)	0.0426	[-0.2177, 0.303]	0.7482	41	-0.202	[-0.6291, 0.2251]	0.3539	38
long (>= 48 weeks)	0.4588	[0.1329, 0.7846]	0.0058	41	-0.0738	[-0.5242, 0.3765]	0.7479	38
Background Therapy								
monotherapy	Ref				Ref			
add-on therapy	0.1814	[-0.123, 0.4858]	0.2427	41	0.2866	[-0.1532, 0.7264]	0.2015	38

BMI: body mass index; FPG: fasting plasma glucose; SBP: systolic blood pressure; DBP: diastolic blood pressure; HDL-C: high-density lipoprotein cholesterol, TG: triglyceride.

Fig. S7 Partial dependency plot of baseline characteristics with HbA1c change

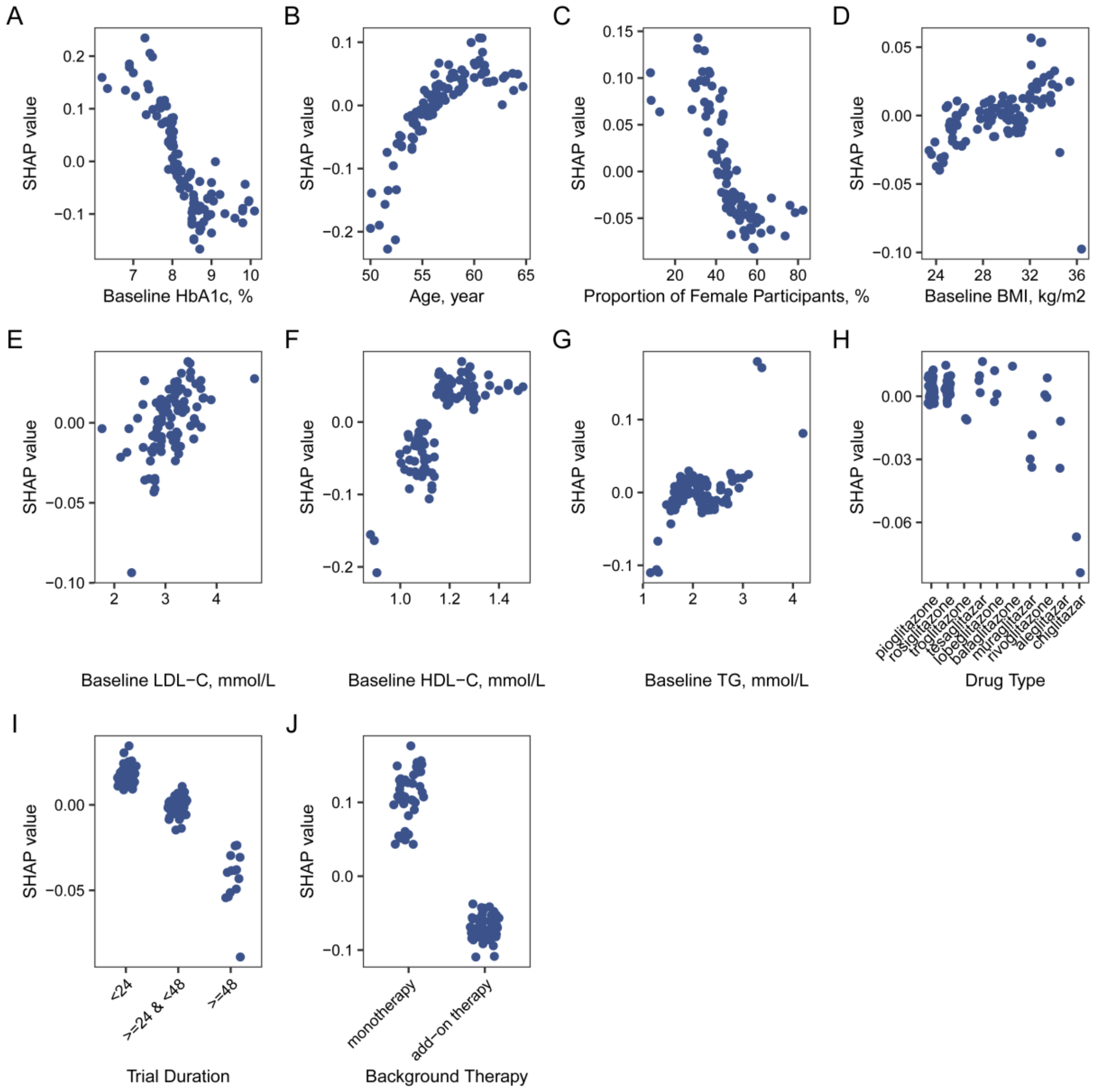

Dependency was quantified by shapley additive explanation (SHAP) values. BMI: body mass index; LDL-C: low-density lipoprotein cholesterol; HDL-C: high-density lipoprotein cholesterol, TG: triglyceride.

Fig. S8 Partial dependency plot of baseline characteristics with FPG change

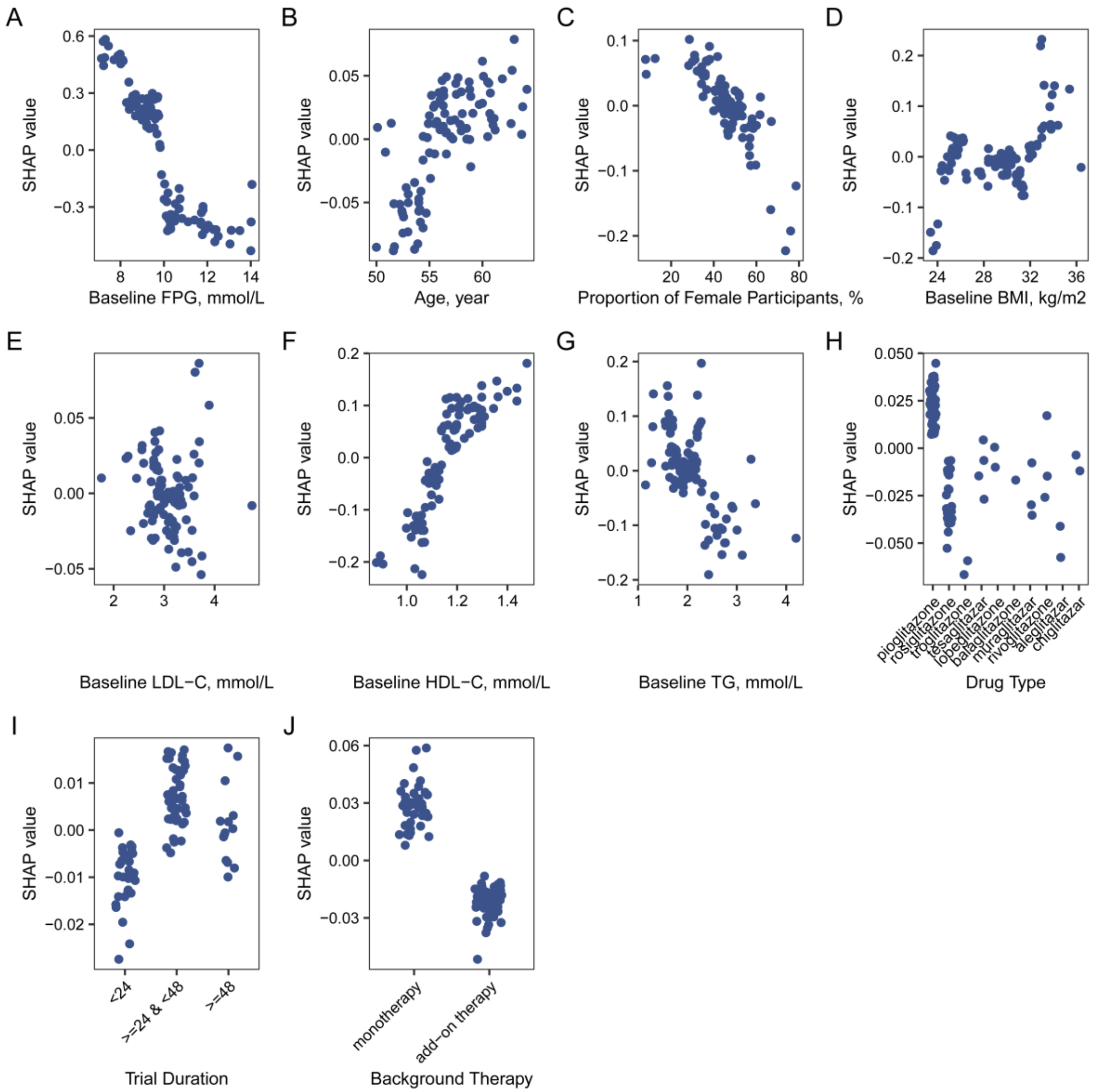

Dependency was quantified by shapley additive explanation (SHAP) values. FPG, fasting plasma glucose; BMI: body mass index; LDL-C: low-density lipoprotein cholesterol; HDL-C: high-density lipoprotein cholesterol, TG: triglyceride.

Section S6 Reference list for included studies

1. Davidson, J.; Mcmorn, S.; Waterhouse, B.; Cobitz, A. A 24-Week, Multicenter, Randomized, Double-Blind, Placebo-Controlled, Parallel-Group Study of the Efficacy and Tolerability of Combination Therapy with Rosiglitazone and Sulfonylurea in African American and Hispanic American Patients with Type 2 Diabetes Inadequately Controlled with Sulfonylurea Monotherapy. Clin. Ther. 2007, 29, 1900–1914. https://doi.org/10.1016/j.clinthera.2007.09.011.

2. Chou, H.S.; Truitt, K.E.; Moberly, J.B.; Merante, D.; Choi, Y.; Mun, Y.; Pfützner, A. A 26-Week, Placebo- and Pioglitazone-Controlled Monotherapy Study of Rivoglitazone in Subjects with Type 2 Diabetes Mellitus. Diabetes Obes. Metab. 2012, 14, 1000–1009. https://doi.org/10.1111/j.1463-1326.2012.01631.x.

3. Truitt, K.E.; Goldberg, R.B.; Rosenstock, J.; Chou, H.S.; Merante, D.; Triscari, J.; Wang, A.C. A 26-Week, Placebo- and Pioglitazone-Controlled, Dose-Ranging Study of Rivoglitazone, a Novel Thiazolidinedione for the Treatment of Type 2 Diabetes. Curr. Med. Res. Opin. 2010, 26, 1321–1331. https://doi.org/10.1185/03007991003715079.

4. Goldberg, R.B.; Kendall, D.M.; Deeg, M.A.; Buse, J.B.; Zagar, A.J.; Pinaire, J.A.; Tan, M.H.; Khan, M.A.; Perez, A.T.; Jacober, S.J. A Comparison of Lipid and Glycemic Effects of Pioglitazone and Rosiglitazone in Patients with Type 2 Diabetes and Dyslipidemia. Diabetes Care 2005, 28, 1547–1554. https://doi.org/10.2337/diacare.28.7.1547.

5. St John Sutton, M.; Rendell, M.; Dandona, P.; Dole, J.F.; Murphy, K.; Patwardhan, R.; Patel, J.; Freed, M. A Comparison of the Effects of Rosiglitazone and Glyburide on Cardiovascular Function and Glycemic Control in Patients with Type 2 Diabetes. Diabetes Care 2002, 25, 2058–2064. https://doi.org/10.2337/diacare.25.11.2058.

6. Bays, H.; McElhattan, J.; Bryzinski, B.S. A Double-Blind, Randomised Trial of Tesaglitazar versus Pioglitazone in Patients with Type 2 Diabetes Mellitus. Diabetes Vasc. Dis. Res. 2007, 4, 181–193. https://doi.org/10.3132/dvdr.2007.039.

7. Ryang, S.; Kim, S.S.; Bae, J.C.; Han, J.M.; Kwon, S.K.; Kim, Y.I.; Nam-Goong, I.S.; Kim, E.S.; Kim, M.K.; Lee, C.W.; et al. A Double-Blind, Randomized Controlled Trial on Glucose-Lowering Effects and Safety of Adding 0.25 or 0.5 Mg Lobeglitazone in Type 2 Diabetes Patients with INadequate Control on Metformin and Dipeptidyl Peptidase-4 Inhibitor Therapy: REFIND Study. Diabetes Obes. Metab. 2022, 24, 1800–1809. https://doi.org/10.1111/dom.14766.

8. Hanefeld, M.; Patwardhan, R.; Jones, N.P. A One-Year Study Comparing the Efficacy and Safety of Rosiglitazone and Glibenclamide in the Treatment of Type 2 Diabetes. Nutr. Metab. Cardiovasc. Dis. 2007, 17, 13–23. https://doi.org/10.1016/j.numecd.2005.12.003.

9. Raskin, P.; Rendell, M.; Riddle, M.C.; Dole, J.F.; Freed, M.I.; Rosenstock, J.; Rosiglitazone Clinical Trials Study, G. A Randomized Trial of Rosiglitazone Therapy in Patients with Inadequately Controlled Insulin-Treated Type 2 Diabetes. Diabetes Care 2001, 24, 1226–1232. https://doi.org/10.2337/diacare.24.7.1226.

10. Lü, Z.; Pan, C.; Gao, Y.; Guo, L.; Ning, G.; Liu, Z.; Lu, J.; Jia, P.; Wang, X.; Sun, S.-Y.; et al. A Randomized, Double Blind, Placebo-Controlled, Parallel and Multicenter Study to Evaluate the Safety and Efficacy of Pioglitazone with Sulphonylurea in Type 2 Diabetic Patients. Zhonghua Nei Ke Za Zhi 2011, 50, 826–830.

11. Herz, M.; Johns, D.; Reviriego, J.; Grossman, L.D.; Godin, C.; Duran, S.; Hawkins, F.; Lochnan, H.; Escobar-Jimenez, F.; Hardin, P.A.; et al. A Randomized, Double-Blind, Placebo-Controlled, Clinical Trial of the Effects of Pioglitazone on Glycemic Control and Dyslipidemia in Oral Antihyperglycemic Medication-Naive Patients with Type 2 Diabetes Mellitus. Clin. Ther. 2003, 25, 1074–1095. https://doi.org/10.1016/s0149-2918(03)80068-1.

12. Kong, A.P.; Yamasaki, A.; Ozaki, R.; Saito, H.; Asami, T.; Ohwada, S.; Ko, G.T.; Wong, C.K.; Leung, G.T.; Lee, K.F.;

et al. A Randomized-Controlled Trial to Investigate the Effects of Rivoglitazone, a Novel PPAR Gamma Agonist on Glucose-Lipid Control in Type 2 Diabetes. Diabetes Obes. Metab. 2011, 13, 806–813. https://doi.org/10.1111/j.1463-1326.2011.01411.x.

13. Wolffenbuttel, B.H.; Gomis, R.; Squatrito, S.; Jones, N.P.; Patwardhan, R.N. Addition of Low-Dose Rosiglitazone to Sulphonylurea Therapy Improves Glycaemic Control in Type 2 Diabetic Patients. Diabet. Med. 2000, 17, 40–47. https://doi.org/10.1046/j.1464-5491.2000.00224.x.

14. Davidson, J.A.; Perez, A.; Zhang, J. Addition of Pioglitazone to Stable Insulin Therapy in Patients with Poorly Controlled Type 2 Diabetes: Results of a Double-Blind, Multicentre, Randomized Study. Diabetes Obes. Metab. 2006, 8, 164–174. https://doi.org/10.1111/j.1463-1326.2005.00499.x.

15. Zhu, X.X.; Pan, C.Y.; Li, G.W.; Shi, H.L.; Tian, H.; Yang, W.Y.; Jiang, J.; Sun, X.C.; Davies, C.; Chow, W.H. Addition of Rosiglitazone to Existing Sulfonylurea Treatment in Chinese Patients with Type 2 Diabetes and Exposure to Hepatitis B or C. Diabetes Technol. Ther. 2003, 5, 33–42. https://doi.org/10.1089/152091503763816445.

16. Stirban, A.O.; Andjelkovic, M.; Heise, T.; Nosek, L.; Fischer, A.; Gastaldelli, A.; Herz, M. Aleglitazar, a Dual Peroxisome Proliferator-Activated Receptor-Alpha/Gamma Agonist, Improves Insulin Sensitivity, Glucose Control and Lipid Levels in People with Type 2 Diabetes: Findings from a Randomized, Double-Blind Trial. Diabetes Obes. Metab. 2016, 18, 711–715. https://doi.org/10.1111/dom.12620.

17. Wallace, T.M.; Levy, J.C.; Matthews, D.R. An Increase in Insulin Sensitivity and Basal Beta-Cell Function in Diabetic Subjects Treated with Pioglitazone in a Placebo-Controlled Randomized Study. Diabet. Med. 2004, 21, 568–576. https://doi.org/10.1111/j.1464-5491.2004.01218.x.

18. Vongthavaravat, V.; Wajchenberg, B.L.; Waitman, J.N.; Quimpo, J.A.; Menon, P.S.; Ben Khalifa, F.; Chow, W.H.; Study, G. An International Study of the Effects of Rosiglitazone plus Sulphonylurea in Patients with Type 2 Diabetes. Curr. Med. Res. Opin. 2002, 18, 456–461. https://doi.org/10.1185/030079902125001236.

19. Satoh, N.; Ogawa, Y.; Usui, T.; Tagami, T.; Kono, S.; Uesugi, H.; Sugiyama, H.; Sugawara, A.; Yamada, K.; Shimatsu, A.; et al. Antiatherogenic Effect of Pioglitazone in Type 2 Diabetic Patients Irrespective of the Responsiveness to Its Antidiabetic Effect. Diabetes Care 2003, 26, 2493–2499. https://doi.org/10.2337/diacare.26.9.2493.

20. Kadoglou, N.P.; Iliadis, F.; Angelopoulou, N.; Perrea, D.; Liapis, C.D.; Alevizos, M. Beneficial Effects of Rosiglitazone on Novel Cardiovascular Risk Factors in Patients with Type 2 Diabetes Mellitus. Diabet. Med. 2008, 25, 333–340. https://doi.org/10.1111/j.1464-5491.2007.02375.x.

21. Bertrand, O.F.; Poirier, P.; Rodes-Cabau, J.; Rinfret, S.; Title, L.M.; Dzavik, V.; Natarajan, M.; Angel, J.; Batalla, N.; Almeras, N.; et al. Cardiometabolic Effects of Rosiglitazone in Patients with Type 2 Diabetes and Coronary Artery Bypass Grafts: A Randomized Placebo-Controlled Clinical Trial. Atherosclerosis 2010, 211, 565–573. https://doi.org/10.1016/j.atherosclerosis.2010.06.005.

22. Jia, W.; Ma, J.; Miao, H.; Wang, C.; Wang, X.; Li, Q.; Lu, W.; Yang, J.; Zhang, L.; Yang, J.; et al. Chiglitazar Monotherapy with Sitagliptin as an Active Comparator in Patients with Type 2 Diabetes: A Randomized, Double-Blind, Phase 3 Trial (CMAS). Sci. Bull. 2021, 66, 1581–1590. https://doi.org/10.1016/j.scib.2021.02.027.

23. Wei, J.; Tang, Q.; Liu, L.; Bin, J. Combination of Peroxisome Proliferator-Activated Receptor Alpha/Gamma Agonists May Benefit Type 2 Diabetes Patients with Coronary Artery Disease through Inhibition of Inflammatory Cytokine Secretion. Exp. Ther. Med. 2013, 5, 783–788. https://doi.org/10.3892/etm.2013.891.

24. Raskin, P.; McGill, J.; Saad, M.F.; Cappleman, J.M.; Kaye, W.; Khutoryansky, N.; Hale, P.M. Combination Therapy for Type 2 Diabetes: Repaglinide plus Rosiglitazone. Diabet. Med. 2004, 21, 329–335. https://doi.org/10.1111/j.1464-5491.2004.01143.x.

25. Rosenstock, J.; Shen, S.G.; Gatlin, M.R.; Foley, J.E. Combination Therapy with Nateglinide and a Thiazolidinedione Improves Glycemic Control in Type 2 Diabetes. Diabetes Care 2002, 25, 1529–1533. https://doi.org/10.2337/diacare.25.9.1529.

26. Veleba, J.; Kopecky, J.; Janovska, P.; Kuda, O.; Malinska, H.; Flachs, P.; Fiserova, E.; Bardova, K.; Bryhn, M.; Kopecky, J.; et al. Combined Intervention with Pioglitazone and N-3 Fatty Acids in Metformin-Treated Diabetic Patients: Improvement of Metabolic Flexibility. Diabetes 2016, 65, A198–A199. https://doi.org/10.2337/db16-652-860.

27. Esteghamati, A.; Afarideh, M.; Feyzi, S.; Noshad, S.; Nakhjavani, M. Comparative Effects of Metformin and Pioglitazone on Fetuin-A and Osteoprotegerin Concentrations in Patients with Newly Diagnosed Diabetes: A Randomized Clinical Trial. Diabetes Metab. Syndr. 2015, 9, 258–265. https://doi.org/10.1016/j.dsx.2014.09.009.

28. Raman, R.B.; Kumari, A.; Singh, S.P. Comparative Study of Efficacy and Safety of Lobeglitazone versus Pioglitazone as Add on Therapy to Metformin and Vildagliptin in Patients of Type 2 Diabetes Mellitus. Int. J. Pharm. Sci. Rev. Res. 2023, 81, 136–141. https://doi.org/10.47583/ijpsrr.2023.v81i02.023.

29. Kato, T.; Sawai, Y.; Kanayama, H.; Taguchi, H.; Terabayashi, T.; Taki, F.; Yamada, K.; Yamazaki, Y.; Hayakawa, N.; Suzuki, A.; et al. Comparative Study of Low-Dose Pioglitazone or Metformin Treatment in Japanese Diabetic Patients with Metabolic Syndrome. Exp. Clin. Endocrinol. Diabetes 2009, 117, 593–599. https://doi.org/10.1055/s-0029-1202792.

30. Takihata, M.; Nakamura, A.; Tajima, K.; Inazumi, T.; Komatsu, Y.; Tamura, H.; Yamazaki, S.; Kondo, Y.; Yamada, M.; Kimura, M.; et al. Comparative Study of Sitagliptin with Pioglitazone in Japanese Type 2 Diabetic Patients: The COMPASS Randomized Controlled Trial. Diabetes Obes. Metab. 2013, 15, 455–462. https://doi.org/10.1111/dom.12055.

31. Jameshorani, M.; Sayari, S.; Kiahashemi, N.; Motamed, N. Comparative Study on Adding Pioglitazone or Sitagliptin to Patients with Type 2 Diabetes Mellitus Insufficiently Controlled with Metformin. Open Access Maced. J. Med. Sci. 2017, 5, 955–962. https://doi.org/10.3889/oamjms.2017.193.

32. Yoneda, M.; Honda, Y.; Ogawa, Y.; Kessoku, T.; Kobayashi, T.; Imajo, K.; Ozaki, A.; Nogami, A.; Taguri, M.; Yamanaka, T.; et al. Comparing the Effects of Tofogliflozin and Pioglitazone in Non-Alcoholic Fatty Liver Disease Patients with Type 2 Diabetes Mellitus (ToPiND Study): A Randomized Prospective Open-Label Controlled Trial. BMJ Open Diabetes Res. Care 2021, 9, e001990. https://doi.org/10.1136/bmjdrc-2020-001990.

33. Erem, C.; Ozbas, H.M.; Nuhoglu, I.; Deger, O.; Civan, N.; Ersoz, H.O. Comparison of Effects of Gliclazide, Metformin and Pioglitazone Monotherapies on Glycemic Control and Cardiovascular Risk Factors in Patients with Newly Diagnosed Uncontrolled Type 2 Diabetes Mellitus. Exp. Clin. Endocrinol. Diabetes 2014, 122, 295–302. https://doi.org/10.1055/s-0034-1370989.

34. Hamann, A.; Garcia-Puig, J.; Paul, G.; Donaldson, J.; Stewart, M. Comparison of Fixed-Dose Rosiglitazone/Metformin Combination Therapy with Sulphonylurea plus Metformin in Overweight Individuals with Type 2 Diabetes Inadequately Controlled on Metformin Alone. Exp. Clin. Endocrinol. Diabetes 2008, 116, 6–13.

https://doi.org/10.1055/s-2007-984441.

35. Perriello, G.; Pampanelli, S.; Di Pietro, C.; Brunetti, P. Comparison of Glycaemic Control over 1 Year with Pioglitazone or Gliclazide in Patients with Type 2 Diabetes. Diabet. Med. 2006, 23, 246–252. https://doi.org/10.1111/j.1464-5491.2006.01801.x.

36. Strowig, S.M.; Aviles-Santa, M.L.; Raskin, P. Comparison of Insulin Monotherapy and Combination Therapy with Insulin and Metformin or Insulin and Troglitazone in Type 2 Diabetes. Diabetes Care 2002, 25, 1691–1698. https://doi.org/10.2337/diacare.25.10.1691.

37. Yamanouchi, T.; Sakai, T.; Igarashi, K.; Ichiyanagi, K.; Watanabe, H.; Kawasaki, T. Comparison of Metabolic Effects of Pioglitazone, Metformin, and Glimepiride over 1 Year in Japanese Patients with Newly Diagnosed Type 2 Diabetes. Diabet. Med. 2005, 22, 980–985. https://doi.org/10.1111/j.1464-5491.2005.01656.x.

38. Fujitaka, K.; Otani, H.; Jo, F.; Jo, H.; Nomura, E.; Iwasaki, M.; Nishikawa, M.; Iwasaka, T. Comparison of Metabolic Profile and Adiponectin Level with Pioglitazone versus Voglibose in Patients with Type-2 Diabetes Mellitus Associated with Metabolic Syndrome. Endocr. J. 2011, 58, 425–432. https://doi.org/10.1507/endocrj.k10e-327.

39. Nagasaka, S.; Aiso, Y.; Yoshizawa, K.; Ishibashi, S. Comparison of Pioglitazone and Metformin Efficacy Using Homeostasis Model Assessment. Diabet. Med. 2004, 21, 136–141. https://doi.org/10.1111/j.1464-5491.2004.01083.x.

40. Xu, W.; Bi, Y.; Sun, Z.; Li, J.; Guo, L.; Yang, T.; Wu, G.; Shi, L.; Feng, Z.; Qiu, L.; et al. Comparison of the Effects on Glycaemic Control and Beta-Cell Function in Newly Diagnosed Type 2 Diabetes Patients of Treatment with Exenatide, Insulin or Pioglitazone: A Multicentre Randomized Parallel-Group Trial (the CONFIDENCE Study). J. Intern. Med. 2015, 277, 137–150. https://doi.org/10.1111/joim.12293.

41. Yoon, K.H.; Shin, J.A.; Kwon, H.S.; Lee, S.H.; Min, K.W.; Ahn, Y.B.; Yoo, S.J.; Ahn, K.J.; Park, S.W.; Lee, K.W.; et al. Comparison of the Efficacy of Glimepiride, Metformin, and Rosiglitazone Monotherapy in Korean Drug-Naive Type 2 Diabetic Patients: The Practical Evidence of Antidiabetic Monotherapy Study. Diabetes Metab. J. 2011, 35, 26–33. https://doi.org/10.4093/dmj.2011.35.1.26.

42. Baksi, A.; James, R.E.; Zhou, B.; Nolan, J.J. Comparison of Uptitration of Gliclazide with the Addition of Rosiglitazone to Gliclazide in Patients with Type 2 Diabetes Inadequately Controlled on Half-Maximal Doses of a Sulphonylurea. Acta Diabetol. 2004, 41, 63–69. https://doi.org/10.1007/s00592-004-0146-y.

43. Kim, J.H.; Kim, S.S.; Baek, H.S.; Lee, I.K.; Chung, D.J.; Sohn, H.S.; Bae, H.Y.; Kim, M.K.; Park, J.H.; Choi, Y.S.; et al. Comparison of Vildagliptin and Pioglitazone in Korean Patients with Type 2 Diabetes Inadequately Controlled with Metformin. Diabetes Metab. J. 2016, 40, 230–239. https://doi.org/10.4093/dmj.2016.40.3.230.

44. Bolli, G.; Dotta, F.; Colin, L.; Minic, B.; Goodman, M. Comparison of Vildagliptin and Pioglitazone in Patients with Type 2 Diabetes Inadequately Controlled with Metformin. Diabetes Obes. Metab. 2009, 11, 589–595. https://doi.org/10.1111/j.1463-1326.2008.01023.x.

45. Reynolds, L.R.; Kingsley, F.J.; Karounos, D.G.; Tannock, L.R. Differential Effects of Rosiglitazone and Insulin Glargine on Inflammatory Markers, Glycemic Control, and Lipids in Type 2 Diabetes. Diabetes Res. Clin. Pract. 2007, 77, 180–187. https://doi.org/10.1016/j.diabres.2006.12.011.

46. Virtanen, K.A.; Hällsten, K.; Parkkola, R.; Janatuinen, T.; Lönnqvist, F.; Viljanen, T.; Rönnemaa, T.; Knuuti, J.; Huupponen, R.; Lönnroth, P.; et al. Differential Effects of Rosiglitazone and Metformin on Adipose Tissue Distribution

and Glucose Uptake in Type 2 Diabetic Subjects. Diabetes 2003, 52, 283–290. https://doi.org/10.2337/diabetes.52.2.283.

47. Derosa, G.; Maffioli, P.; Salvadeo, S.A.; Ferrari, I.; Gravina, A.; Mereu, R.; Palumbo, I.; D’Angelo, A.; Cicero, A.F. Direct Comparison among Oral Hypoglycemic Agents and Their Association with Insulin Resistance Evaluated by Euglycemic Hyperinsulinemic Clamp: The 60’s Study. Metabolism 2009, 58, 1059–1066. https://doi.org/10.1016/j.metabol.2009.03.007.

48. Miyazaki, Y.; Matsuda, M.; DeFronzo, R.A. Dose-Response Effect of Pioglitazone on Insulin Sensitivity and Insulin Secretion in Type 2 Diabetes. Diabetes Care 2002, 25, 517–523. https://doi.org/10.2337/diacare.25.3.517.

49. Iwamoto, Y.; Kosaka, K.; Kuzuya, T.; Akanuma, Y.; Shigeta, Y.; Kaneko, T. Effect of Combination Therapy of Troglitazone and Sulphonylureas in Patients with Type 2 Diabetes Who Were Poorly Controlled by Sulphonylurea Therapy Alone. Diabet. Med. 1996, 13, 365–370. https://doi.org/10.1002/(SICI)1096-9136(199604)13:4<365::AID-DIA19>3.0.CO;2-M.

50. Rajagopalan, S.; Dutta, P.; Hota, D.; Bhansali, A.; Srinivasan, A.; Chakrabarti, A. Effect of Low Dose Pioglitazone on Glycemic Control and Insulin Resistance in Type 2 Diabetes: A Randomized, Double Blind, Clinical Trial. Diabetes Res. Clin. Pract. 2015, 109, e32–e35. https://doi.org/10.1016/j.diabres.2015.05.030.

51. Fonseca, V.; Rosenstock, J.; Patwardhan, R.; Salzman, A. Effect of Metformin and Rosiglitazone Combination Therapy in Patients with Type 2 Diabetes Mellitus: A Randomized Controlled Trial. JAMA 2000, 283, 1695–1702. https://doi.org/10.1001/jama.283.13.1695.

52. Yang, L.; Song, M.Q.; Zhang, Q.L.; Shou, L.; Zang, S.F.; Yang, Y.L. Effect of Piglitazone and Metformin on Retinol-Binding Protein-4 and Adiponectin in Patients with Type 2 Diabetes Mellitus Complicated with Non-Alcohol Fatty Acid Liver Diseases. Zhongguo Yi Xue Ke Xue Yuan Xue Bao 2014, 36, 309–312. https://doi.org/10.3881/j.issn.1000-503X.2014.03.015.

53. Derosa, G.; Mereu, R.; D’Angelo, A.; Salvadeo, S.A.; Ferrari, I.; Fogari, E.; Gravina, A.; Palumbo, I.; Maffioli, P.; Randazzo, S.; et al. Effect of Pioglitazone and Acarbose on Endothelial Inflammation Biomarkers during Oral Glucose Tolerance Test in Diabetic Patients Treated with Sulphonylureas and Metformin. J. Clin. Pharm. Ther. 2010, 35, 565–579. https://doi.org/10.1111/j.1365-2710.2009.01132.x.

54. Pavo, I.; Jermendy, G.; Varkonyi, T.T.; Kerenyi, Z.; Gyimesi, A.; Shoustov, S.; Shestakova, M.; Herz, M.; Johns, D.; Schluchter, B.J.; et al. Effect of Pioglitazone Compared with Metformin on Glycemic Control and Indicators of Insulin Sensitivity in Recently Diagnosed Patients with Type 2 Diabetes. J. Clin. Endocrinol. Metab. 2003, 88, 1637–1645. https://doi.org/10.1210/jc.2002-021786.

55. Hao, Y.; Zhang, L.; He, X. Effect of Pioglitazone on Plasminogen Activator Inhibitor-1 of Plasma in Type 2 Diabetic Mellitus Patients. Med. J. Wuhan Univ. 2005, 26, 636–638+645.

56. Genovese, S.; De Berardis, G.; Nicolucci, A.; Mannucci, E.; Evangelista, V.; Totani, L.; Pellegrini, F.; Ceriello, A. Effect of Pioglitazone versus Metformin on Cardiovascular Risk Markers in Type 2 Diabetes. Adv. Ther. 2013, 30, 190–202. https://doi.org/10.1007/s12325-013-0003-x.

57. Miyazaki, Y.; Glass, L.; Triplitt, C.; Matsuda, M.; Cusi, K.; Mahankali, A.; Mahankali, S.; Mandarino, L.J.; DeFronzo, R.A. Effect of Rosiglitazone on Glucose and Non-Esterified Fatty Acid Metabolism in Type II Diabetic Patients. Diabetologia 2001, 44, 2210–2219. https://doi.org/10.1007/s001250100031.

58. Carey, D.G.; Cowin, G.J.; Galloway, G.J.; Jones, N.P.; Richards, J.C.; Biswas, N.; Doddrell, D.M. Effect of Rosiglitazone on Insulin Sensitivity and Body Composition in Type 2 Diabetic Patients. Obes. Res. 2002, 10, 1008–1015. https://doi.org/10.1038/oby.2002.137.

59. Goldstein, B.J.; Rosenstock, J.; Anzalone, D.; Tou, C.; Ohman, K.P. Effect of Tesaglitazar, a Dual PPAR Alpha/Gamma Agonist, on Glucose and Lipid Abnormalities in Patients with Type 2 Diabetes: A 12-Week Dose-Ranging Trial. Curr. Med. Res. Opin. 2006, 22, 2575–2590. https://doi.org/10.1185/030079906x154169.

60. Henry, R.R.; Lincoff, A.M.; Mudaliar, S.; Rabbia, M.; Chognot, C.; Herz, M. Effect of the Dual Peroxisome Proliferator-Activated Receptor-Alpha/Gamma Agonist Aleglitazar on Risk of Cardiovascular Disease in Patients with Type 2 Diabetes (SYNCHRONY): A Phase II, Randomised, Dose-Ranging Study. Lancet 2009, 374, 126–135. https://doi.org/10.1016/S0140-6736(09)60870-9.

61. Triwatana, W.; Satirapoj, B.; Supasyndh, O.; Nata, N. Effect of Pioglitazone on Serum FGF23 Levels among Patients with Diabetic Kidney Disease: A Randomized Controlled Trial. Int. Urol. Nephrol. 2023, 55, 1255–1262. https://doi.org/10.1007/s11255-022-03420-0.

62. Derosa, G.; Cicero, A.F.; D'Angelo, A.; Gaddi, A.; Ciccarelli, L.; Piccinni, M.N.; Salvadeo, S.A.; Pricolo, F.; Ferrari, I.; Gravina, A.; et al. Effects of 1 Year of Treatment with Pioglitazone or Rosiglitazone Added to Glimepiride on Lipoprotein (a) and Homocysteine Concentrations in Patients with Type 2 Diabetes Mellitus and Metabolic Syndrome: A Multicenter, Randomized, Double-Blind, Controlled Clinical Trial. Clin. Ther. 2006, 28, 679–688. https://doi.org/10.1016/j.clinthera.2006.05.012.

63. Hartemann-Heurtier, A.; Halbron, M.; Golmard, J.-L.; Jacqueminet, S.; Bastard, J.-P.; Rouault, C.; Ayed, A.; Pieroni, L.; Clément, K.; Grimaldi, A. Effects of Bed-Time Insulin versus Pioglitazone on Abdominal Fat Accumulation, Inflammation and Gene Expression in Adipose Tissue in Patients with Type 2 Diabetes. Diabetes Res. Clin. Pract. 2009, 86, 37–43. https://doi.org/10.1016/j.diabres.2009.06.028.

64. Sathyanarayana, P.; Jogi, M.; Muthupillai, R.; Krishnamurthy, R.; Samson, S.L.; Bajaj, M. Effects of Combined Exenatide and Pioglitazone Therapy on Hepatic Fat Content in Type 2 Diabetes. Obesity 2011, 19, 2310–2315. https://doi.org/10.1038/oby.2011.152.

65. Wang, H.-Y.; Zou, D.-J.; Lin, Z.-S. Effects of Different Dosages of Pioglitazone Hydrochloride on Insulin Sensitivity of Patients with Diabetes. Pharm. Care Res. 2004, 4, 142–144.

66. Bhagat, P.; Nigam, N.; Jalota, K.; Ahmad, S.S.; Kumar, S. Effects of Either Sitagliptin or Pioglitazone Addition on Metformin in Patients with Uncontrolled Type 2 Diabetic Mellitus. Int. J. Pharm. Clin. Res. 2022, 14, 707–714.

67. DeFronzo, R.A.; Triplitt, C.; Qu, Y.; Lewis, M.S.; Maggs, D.; Glass, L.C. Effects of Exenatide plus Rosiglitazone on β-Cell Function and Insulin Sensitivity in Subjects with Type 2 Diabetes on Metformin. Diabetes Care 2010, 33, 951–957. https://doi.org/10.2337/dc09-1521.

68. Xiao, C.C.; Ren, A.; Yang, J.; Ye, S.D.; Xing, X.N.; Li, S.M.; Chen, C.; Chen, R.P. Effects of Pioglitazone and Glipizide on Platelet Function in Patients with Type 2 Diabetes. Eur. Rev. Med. Pharmacol. Sci. 2015, 19, 963–970.

69. Naka, K.K.; Papathanassiou, K.; Bechlioulis, A.; Pappas, K.; Kazakos, N.; Kanioglou, C.; Kostoula, A.; Vezyraki, P.; Makriyiannis, D.; Tsatsoulis, A.; et al. Effects of Pioglitazone and Metformin on Vascular Endothelial Function in Patients with Type 2 Diabetes Treated with Sulfonylureas. Diabetes Vasc. Dis. Res. 2012, 9, 52–58. https://doi.org/10.1177/1479164111424515.

70. Teramoto, T.; Yamada, N.; Shirai, K.; Saito, Y. Effects of Pioglitazone Hydrochloride on Japanese Patients with Type 2 Diabetes Mellitus. J. Atheroscler. Thromb. 2007, 14, 86–93. https://doi.org/10.5551/jat.14.86.

71. Sourij, H.; Zweiker, R.; Wascher, T.C. Effects of Pioglitazone on Endothelial Function, Insulin Sensitivity, and Glucose Control in Subjects With Coronary Artery Disease and New-Onset Type 2 Diabetes. Diabetes Care 2006, 29, 1039–1045. https://doi.org/10.2337/dc05-2226.

72. Weissman, P.; Goldstein, B.J.; Rosenstock, J.; Waterhouse, B.; Cobitz, A.R.; Wooddell, M.J.; Strow, L.J. Effects of Rosiglitazone Added to Submaximal Doses of Metformin Compared with Dose Escalation of Metformin in Type 2 Diabetes: The EMPIRE Study. Curr. Med. Res. Opin. 2005, 21, 2029–2035. https://doi.org/10.1185/030079905X74844.

73. Iwamoto, Y.; Kosaka, K.; Kuzuya, T.; Akanuma, Y.; Shigeta, Y.; Kaneko, T. Effects of Troglitazone: A New Hypoglycemic Agent in Patients with NIDDM Poorly Controlled by Diet Therapy. Diabetes Care 1996, 19, 151–156. https://doi.org/10.2337/diacare.19.2.151.

74. Erande, S.; Mukhopadhyay, J.; Dange, A.; Deogaonkar, A.; Birla, A.; Doshi, C.; Revankar, S.; B, S.S.; Kumar, N.; Kadam, P.V. Efficacy and Safety of a Fixed-Dose Combination of Vildagliptin and Pioglitazone in Indian Patients With Type 2 Diabetes Mellitus: A Randomized, Open-Label, Comparative, Phase III Study. Cureus 2023, 15, e44548. https://doi.org/10.7759/cureus.44548.

75. Liu, S.-C.; Chien, K.-L.; Wang, C.-H.; Chen, W.-C.; Cleunghen, C.-H. Efficacy and Safety of Adding Pioglitazone or Sitagliptin to Patients with Type 2 Diabetes Insufficiently Controlled with Metformin and a Sulfonylurea. Endocr. Pract. 2013, 19, 980–988. https://doi.org/10.4158/EP13148.OR.

76. Ji, L.; Song, W.; Fang, H.; Li, W.; Geng, J.; Wang, Y.; Guo, L.; Cai, H.; Yang, T.; Li, H.; et al. Efficacy and Safety of Chiglitazar, a Novel Peroxisome Proliferator-Activated Receptor Pan-Agonist, in Patients with Type 2 Diabetes: A Randomized, Double-Blind, Placebo-Controlled, Phase 3 Trial (CMAP). Sci. Bull. 2021, 66, 1571–1580. https://doi.org/10.1016/j.scib.2021.03.019.

77. Russell-Jones, D.; Cuddihy, R.M.; Hanefeld, M.; Kumar, A.; Gonzalez, J.G.; Chan, M.; Wolka, A.M.; Boardman, M.K.; Group, D.-S. Efficacy and Safety of Exenatide Once Weekly versus Metformin, Pioglitazone, and Sitagliptin Used as Monotherapy in Drug-Naive Patients with Type 2 Diabetes (DURATION-4): A 26-Week Double-Blind Study. Diabetes Care 2012, 35, 252–258. https://doi.org/10.2337/dc11-1107.

78. Bergenstal, R.M.; Wysham, C.; MacConell, L.; Malloy, J.; Walsh, B.; Yan, P.; Wilhelm, K.; Malone, J.; Porter, L.E. Efficacy and Safety of Exenatide Once Weekly versus Sitagliptin or Pioglitazone as an Adjunct to Metformin for Treatment of Type 2 Diabetes (DURATION-2): A Randomised Trial. Lancet 2010, 376, 431–439. https://doi.org/10.1016/S0140-6736(10)60590-9.

79. Kim, S.G.; Kim, D.M.; Woo, J.-T.; Jang, H.C.; Chung, C.H.; Ko, K.S.; Park, J.H.; Park, Y.S.; Kim, S.J.; Choi, D.S. Efficacy and Safety of Lobeglitazone Monotherapy in Patients with Type 2 Diabetes Mellitus over 24-Weeks: A Multicenter, Randomized, Double-Blind, Parallel-Group, Placebo Controlled Trial. PLoS ONE 2014, 9, e92843. https://doi.org/10.1371/journal.pone.0092843.

80. Rubin, C.J.; Viraswami-Appanna, K.; Fiedorek, F.T. Efficacy and Safety of Muraglitazar: A Double-Blind, 24-Week, Dose-Ranging Study in Patients with Type 2 Diabetes. Diabetes Vasc. Dis. Res. 2009, 6, 205–215. https://doi.org/10.1177/1479164109336048.

81. Kaku, K.; Katou, M.; Igeta, M.; Ohira, T.; Sano, H. Efficacy and Safety of Pioglitazone Added to Alogliptin in Japanese Patients with Type 2 Diabetes Mellitus: A Multicentre, Randomized, Double-Blind, Parallel-Group, Comparative Study. Diabetes Obes. Metab. 2015, 17, 1198–1201. https://doi.org/10.1111/dom.12555.

82. Rosenstock, J.; Einhorn, D.; Hershon, K.; Glazer, N.B.; Yu, S.; Pioglitazone 014 Study, G. Efficacy and Safety of Pioglitazone in Type 2 Diabetes: A Randomised, Placebo-Controlled Study in Patients Receiving Stable Insulin Therapy. Int. J. Clin. Pract. 2002, 56, 251–257.

83. Kim, J.M.; Kim, S.S.; Kim, J.H.; Kim, M.K.; Kim, T.N.; Lee, S.H.; Lee, C.W.; Park, J.Y.; Kim, E.S.; Lee, K.J.; et al. Efficacy and Safety of Pioglitazone versus Glimepiride after Metformin and Alogliptin Combination Therapy: A Randomized, Open-Label, Multicenter, Parallel-Controlled Study. Diabetes Metab. J. 2020, 44, 67–77. https://doi.org/10.4093/dmj.2018.0274.

84. Schernthaner, G.; Matthews, D.R.; Charbonnel, B.; Hanefeld, M.; Brunetti, P.; Quartet Study, G. Efficacy and Safety of Pioglitazone versus Metformin in Patients with Type 2 Diabetes Mellitus: A Double-Blind, Randomized Trial. J. Clin. Endocrinol. Metab. 2004, 89, 6068–6076. https://doi.org/10.1210/jc.2003-030861.

85. Perez, A.; Zhao, Z.; Jacks, R.; Spanheimer, R. Efficacy and Safety of Pioglitazone/Metformin Fixed-Dose Combination Therapy Compared with Pioglitazone and Metformin Monotherapy in Treating Patients with T2DM. Curr. Med. Res. Opin. 2009, 25, 2915–2923. https://doi.org/10.1185/03007990903350011.

86. Gomez-Perez, F.J.; Fanghanel-Salmon, G.; Antonio Barbosa, J.; Montes-Villarreal, J.; Berry, R.A.; Warsi, G.; Gould, E.M. Efficacy and Safety of Rosiglitazone plus Metformin in Mexicans with Type 2 Diabetes. Diabetes Metab. Res. Rev. 2002, 18, 127–134. https://doi.org/10.1002/dmrr.264.

87. Scott, R.; Loeys, T.; Davies, M.J.; Engel, S.S. Efficacy and Safety of Sitagliptin When Added to Ongoing Metformin Therapy in Patients with Type 2 Diabetes*. Diabetes Obes. Metab. 2008, 10, 959–969. https://doi.org/10.1111/j.1463-1326.2007.00839.x.

88. Rodrigues, A.; Kamath, L.; Choksi, S.; R, R. Efficacy and Safety of Standard Dose Pioglitazone versus Low-Dose Pioglitazone as an Add on Treatment in Type 2 Diabetes Mellitus: A Randomized Controlled Trial. Natl. J. Physiol. Pharm. Pharmacol. 2022, 13, 536–541. https://doi.org/10.5455/njppp.2023.13.07367202230072022.

89. Henriksen, K.; Byrjalsen, I.; Qvist, P.; Beck-Nielsen, H.; Hansen, G.; Riis, B.J.; Perrild, H.; Svendsen, O.L.; Gram, J.; Karsdal, M.A.; et al. Efficacy and Safety of the PPARγ Partial Agonist Balaglitazone Compared with Pioglitazone and Placebo: A Phase III, Randomized, Parallel-Group Study in Patients with Type 2 Diabetes on Stable Insulin Therapy. Diabetes Metab. Res. Rev. 2011, 27, 392–401. https://doi.org/10.1002/dmrr.1187.

90. Rosenstock, J.; Kim, S.W.; Baron, M.A.; Camisasca, R.P.; Cressier, F.; Couturier, A.; Dejager, S. Efficacy and Tolerability of Initial Combination Therapy with Vildagliptin and Pioglitazone Compared with Component Monotherapy in Patients with Type 2 Diabetes. Diabetes Obes. Metab. 2007, 9, 175–185. https://doi.org/10.1111/j.1463-1326.2006.00698.x.

91. Kikuchi, M.; Kaku, K.; Odawara, M.; Momomura, S.; Ishii, R. Efficacy and Tolerability of Rosiglitazone and Pioglitazone in Drug-Naive Japanese Patients with Type 2 Diabetes Mellitus: A Double-Blind, 28 Weeks' Treatment, Comparative Study. Curr. Med. Res. Opin. 2012, 28, 1007–1016. https://doi.org/10.1185/03007995.2012.694361.

92. Henry, R.R.; Buse, J.B.; Wu, H.; Durrwell, L.; Mingrino, R.; Jaekel, K.; El Azzouzi, B.; Andjelkovic, M.; Herz, M. Efficacy, Safety and Tolerability of Aleglitazar in Patients with Type 2 Diabetes: Pooled Findings from Three

Randomized Phase III Trials. Diabetes Obes. Metab. 2015, 17, 560–565. https://doi.org/10.1111/dom.12455.

93. Ratner, R.E.; Parikh, S.; Tou, C.; Group, G.S. Efficacy, Safety and Tolerability of Tesaglitazar When Added to the Therapeutic Regimen of Poorly Controlled Insulin-Treated Patients with Type 2 Diabetes. Diabetes Vasc. Dis. Res. 2007, 4, 214–221. https://doi.org/10.3132/dvdr.2007.042.

94. Umpierrez, G.; Issa, M.; Vlajnic, A. Glimepiride versus Pioglitazone Combination Therapy in Subjects with Type 2 Diabetes Inadequately Controlled on Metformin Monotherapy: Results of a Randomized Clinical Trial. Curr. Med. Res. Opin. 2006, 22, 751–759. https://doi.org/10.1185/030079906X104786.

95. Bae, J.; Huh, J.H.; Lee, M.; Lee, Y.; Lee, B. Glycaemic Control with Add-on Thiazolidinedione or a Sodium-Glucose Co-Transporter-2 Inhibitor in Patients with Type 2 Diabetes after the Failure of an Oral Triple Antidiabetic Regimen: A 24-Week, Randomized Controlled Trial. Diabetes Obes. Metab. 2021, 23, 609–618. https://doi.org/10.1111/dom.14259.

96. Dailey, G.E., 3rd; Noor, M.A.; Park, J.S.; Bruce, S.; Fiedorek, F.T. Glycemic Control with Glyburide/Metformin Tablets in Combination with Rosiglitazone in Patients with Type 2 Diabetes: A Randomized, Double-Blind Trial. Am. J. Med. 2004, 116, 223–229. https://doi.org/10.1016/j.amjmed.2003.07.022.

97. Miyazaki, Y.; Mahankali, A.; Matsuda, M.; Glass, L.; Mahankali, S.; Ferrannini, E.; Cusi, K.; Mandarino, L.J.; DeFronzo, R.A. Improved Glycemic Control and Enhanced Insulin Sensitivity in Type 2 Diabetic Subjects Treated with Pioglitazone. Diabetes Care 2001, 24, 710–719. https://doi.org/10.2337/diacare.24.4.710.

98. Göke, B. Improved Glycemic Control and Lipid Profile in a Randomized Study of Pioglitazone Compared with Acarbose in Patients with Type 2 Diabetes Mellitus. Treat. Endocrinol. 2002, 1, 329–336. https://doi.org/10.2165/00024677-200201050-00005.

99. Rubin, C.J.; Ledeine, J.M.; Fiedorek, F.T. Improvement of Glycaemic and Lipid Profiles with Muraglitazar plus Metformin in Patients with Type 2 Diabetes: An Active-Control Trial with Glimepiride. Diabetes Vasc. Dis. Res. 2008, 5, 168–176. https://doi.org/10.3132/dvdr.2008.028.

100. Kendall, D.M.; Rubin, C.J.; Mohideen, P.; Ledeine, J.M.; Belder, R.; Gross, J.; Norwood, P.; O'Mahony, M.; Sall, K.; Sloan, G.; et al. Improvement of Glycemic Control, Triglycerides, and HDL Cholesterol Levels with Muraglitazar, a Dual (Alpha/Gamma) Peroxisome Proliferator-Activated Receptor Activator, in Patients with Type 2 Diabetes Inadequately Controlled with Metformin Monotherapy: A Double-Blind, Randomized, Pioglitazone-Comparative Study. Diabetes Care 2006, 29, 1016–1023. https://doi.org/10.2337/diacare.2951016.

101. Chou, H.S.; Palmer, J.P.; Jones, A.R.; Waterhouse, B.; Ferreira‐Cornwell, C.; Krebs, J.; Goldstein, B.J. Initial Treatment with Fixed-Dose Combination Rosiglitazone/Glimepiride in Patients with Previously Untreated Type 2 Diabetes. Diabetes Obes. Metab. 2008, 10, 626–637. https://doi.org/10.1111/j.1463-1326.2007.00753.x.

102. Zhang, Y.-N.; Cui, C.; Fan, Y.; Chang, M.-L.; Wu, W.; Yu, W.-G.; Liu, F.-C.; Tan, N.; Zhang, J.-C. Interventional Effects of Rosiglitazone in Type 2 Diabetes Elderly Male Patients Combined with Atherosclerosis. Chin. J. Clin. Rehabil. 2005, 9, 58–61.

103. Nauck, M.A.; di Domenico, M.; Patel, S.; Kobe, M.; Toorawa, R.; Woerle, H.-J. Linagliptin and Pioglitazone Combination Therapy versus Monotherapy with Linagliptin or Pioglitazone: A Randomised, Double-Blind, Parallel-Group, Multinational Clinical Trial. Diabetes Vasc. Dis. Res. 2016, 13, 286–298. https://doi.org/10.1177/1479164116639229.

104. Jin, S.M.; Park, C.Y.; Cho, Y.M.; Ku, B.J.; Ahn, C.W.; Cha, B.S.; Min, K.W.; Sung, Y.A.; Baik, S.H.; Lee, K.W.; et al. Lobeglitazone and Pioglitazone as Add-Ons to Metformin for Patients with Type 2 Diabetes: A 24-Week, Multicentre, Randomized, Double-Blind, Parallel-Group, Active-Controlled, Phase III Clinical Trial with a 28-Week Extension. Diabetes Obes. Metab. 2015, 17, 599–602. https://doi.org/10.1111/dom.12435.

105. Jain, R.; Osei, K.; Kupfer, S.; Perez, A.T.; Zhang, J. Long-Term Safety of Pioglitazone versus Glyburide in Patients with Recently Diagnosed Type 2 Diabetes Mellitus. Pharmacotherapy 2006, 26, 1388–1395. https://doi.org/10.1592/phco.26.10.1388.

106. Matthews, D.R.; Charbonnel, B.H.; Hanefeld, M.; Brunetti, P.; Schernthaner, G. Long-Term Therapy with Addition of Pioglitazone to Metformin Compared with the Addition of Gliclazide to Metformin in Patients with Type 2 Diabetes: A Randomized, Comparative Study. Diabetes Metab. Res. Rev. 2005, 21, 167–174. https://doi.org/10.1002/dmrr.478.

107. Fernandez, M.; Gastaldelli, A.; Triplitt, C.; Hardies, J.; Casolaro, A.; Petz, R.; Tantiwong, P.; Musi, N.; Cersosimo, E.; Ferrannini, E.; et al. Metabolic Effects of Muraglitazar in Type 2 Diabetic Subjects. Diabetes Obes. Metab. 2011, 13, 893–902. https://doi.org/10.1111/j.1463-1326.2011.01429.x.

108. Scherbaum, W.A.; Goke, B.; German Pioglitazone Study, G. Metabolic Efficacy and Safety of Once-Daily Pioglitazone Monotherapy in Patients with Type 2 Diabetes: A Double-Blind, Placebo-Controlled Study. Horm. Metab. Res. 2002, 34, 589–595. https://doi.org/10.1055/s-2002-35421.

109. Buse, J.B.; Rubin, C.J.; Frederich, R.; Viraswami-Appanna, K.; Lin, K.C.; Montoro, R.; Shockey, G.; Davidson, J.A. Muraglitazar, a Dual (Alpha/Gamma) PPAR Activator: A Randomized, Double-Blind, Placebo-Controlled, 24-Week Monotherapy Trial in Adult Patients with Type 2 Diabetes. Clin. Ther. 2005, 27, 1181–1195. https://doi.org/10.1016/j.clinthera.2005.08.005.

110. Hanefeld, M.; Brunetti, P.; Schernthaner, G.H.; Matthews, D.R.; Charbonnel, B.H.; Group, Q.S. One-Year Glycemic Control with a Sulfonylurea plus Pioglitazone versus a Sulfonylurea plus Metformin in Patients with Type 2 Diabetes. Diabetes Care 2004, 27, 141–147. https://doi.org/10.2337/diacare.27.1.141.

111. Kho, J.-S.; Park, S.-J.; Im, S.-I.; Choi, B.-R.; Kwak, C.-H.; Hwang, J.-Y. Peroxisome Proliferator-Activated Receptor Gamma(PPAR-γ) Agonist Improves Endothelial Function in Diabetic Patients with Metabolic Syndrome: Pivotal Role of NOx and Inflammation. Korean Circ. J. 2007, 37, 221–229. https://doi.org/10.4070/kcj.2007.37.5.221.

112. Kawamori, R.; Matsuhisa, M.; Kinoshita, J.; Mochizuki, K.; Niwa, M.; Arisaka, T.; Ikeda, M.; Kubota, M.; Wada, M.; Kanda, T.; et al. Pioglitazone Enhances Splanchnic Glucose Uptake as Well as Peripheral Glucose Uptake in Non-Insulin-Dependent Diabetes Mellitus. AD-4833 Clamp-OGL Study Group. Diabetes Res. Clin. Pract. 1998, 41, 35–43. https://doi.org/10.1016/s0168-8227(98)00056-4.

113. Einhorn, D.; Rendell, M.; Rosenzweig, J.; Egan, J.W.; Mathisen, A.L.; Schneider, R.L. Pioglitazone Hydrochloride in Combination with Metformin in the Treatment of Type 2 Diabetes Mellitus: A Randomized, Placebo-Controlled Study. The Pioglitazone 027 Study Group. Clin. Ther. 2000, 22, 1395–1409. https://doi.org/10.1016/s0149-2918(00)83039-8.

114. Kipnes, M.S.; Krosnick, A.; Rendell, M.S.; Egan, J.W.; Mathisen, A.L.; Schneider, R.L. Pioglitazone Hydrochloride in Combination with Sulfonylurea Therapy Improves Glycemic Control in Patients with Type 2 Diabetes Mellitus: A Randomized, Placebo-Controlled Study. Am. J. Med. 2001, 111, 10–17. https://doi.org/10.1016/s0002-9343(01)00713-6.

115. Ohira, M.; Yamaguchi, T.; Saiki, A.; Ban, N.; Kawana, H.; Nagumo, A.; Murano, T.; Shirai, K.; Tatsuno, I.

Pioglitazone Improves the Cardio-Ankle Vascular Index in Patients with Type 2 Diabetes Mellitus Treated with Metformin. Diabetes Metab. Syndr. Obes. Targets Ther. 2014, 7, 313–319. https://doi.org/10.2147/DMSO.S65275.

116. Gupta, A.K.; Smith, S.R.; Greenway, F.L.; Bray, G.A. Pioglitazone Treatment in Type 2 Diabetes Mellitus When Combined with Portion Control Diet Modifies the Metabolic Syndrome. Diabetes Obes. Metab. 2009, 11, 330–337. https://doi.org/10.1111/j.1463-1326.2008.00965.x.

117. Papathanassiou, K.; Naka, K.K.; Kazakos, N.; Kanioglou, C.; Makriyiannis, D.; Pappas, K.; Katsouras, C.S.; Liveris, K.; Kolettis, T.; Tsatsoulis, A.; et al. Pioglitazone vs Glimepiride: Differential Effects on Vascular Endothelial Function in Patients with Type 2 Diabetes. Atherosclerosis 2009, 205, 221–226. https://doi.org/10.1016/j.atherosclerosis.2008.11.027.

118. Xing, Y.; Ye, S.; Hu, Y.; Chen, Y. Podocyte as a Potential Target of Inflammation: Role of Pioglitazone Hydrochloride in Patients with Type 2 Diabetes. Endocr. Pract. 2012, 18, 493–498. https://doi.org/10.4158/EP11378.OR.

119. Kim, Y.M.; Cha, B.S.; Kim, D.J.; Choi, S.H.; Kim, S.K.; Ahn, C.W.; Lim, S.-K.; Kim, K.R.; Huh, K.B.; Lee, H.C. Predictive Clinical Parameters for Therapeutic Efficacy of Rosiglitazone in Korean Type 2 Diabetes Mellitus. Diabetes Res. Clin. Pract. 2005, 67, 43–52. https://doi.org/10.1016/j.diabres.2004.05.001.

120. Saad, M.F.; Greco, S.; Osei, K.; Lewin, A.J.; Edwards, C.; Nunez, M.; Reinhardt, R.R.; Ragaglitazar Dose-Ranging Study Group. Ragaglitazar Improves Glycemic Control and Lipid Profile in Type 2 Diabetic Subjects: A 12-Week, Double-Blind, Placebo-Controlled Dose-Ranging Study with an Open Pioglitazone Arm. Diabetes Care 2004, 27, 1324–1329. https://doi.org/10.2337/diacare.27.6.1324.

121. Sykes, A.P.; O'Connor-Semmes, R.; Dobbins, R.; Dorey, D.J.; Lorimer, J.D.; Walker, S.; Wilkison, W.O.; Kler, L. Randomized Trial Showing Efficacy and Safety of Twice-Daily Remogliflozin Etabonate for the Treatment of Type 2 Diabetes. Diabetes Obes. Metab. 2015, 17, 94–97. https://doi.org/10.1111/dom.12391.

122. Raskin, P.; Jovanovic, L.; Berger, S.; Schwartz, S.; Woo, V.; Ratner, R. Repaglinide/Troglitazone Combination Therapy: Improved Glycemic Control in Type 2 Diabetes. Diabetes Care 2000, 23, 979–983. https://doi.org/10.2337/diacare.23.7.979.

123. Naka, K.K.; Papathanassiou, K.; Bechlioulis, A.; Pappas, K.; Kazakos, N.; Kanioglou, C.; Papafaklis, M.I.; Kostoula, A.; Vezyraki, P.; Makriyiannis, D.; et al. Rosiglitazone Improves Endothelial Function in Patients with Type 2 Diabetes Treated with Insulin. Diabetes Vasc. Dis. Res. 2011, 8, 195–201. https://doi.org/10.1177/1479164111408628.

124. Patel, J.; Anderson, R.J.; Rappaport, E.B. Rosiglitazone Monotherapy Improves Glycaemic Control in Patients with Type 2 Diabetes: A Twelve-Week, Randomized, Placebo-Controlled Study. Diabetes Obes. Metab. 1999, 1, 165–172. https://doi.org/10.1046/j.1463-1326.1999.00020.x.

125. Lebovitz, H.E.; Dole, J.F.; Patwardhan, R.; Rappaport, E.B.; Freed, M.I. Rosiglitazone Monotherapy Is Effective in Patients with Type 2 Diabetes. J. Clin. Endocrinol. Metab. 2001, 86, 280–288. https://doi.org/10.1210/jcem.86.1.7157.

126. Khanolkar, M.P.; Morris, R.H.; Thomas, A.W.; Bolusani, H.; Roberts, A.W.; Geen, J.; Jackson, S.K.; Evans, L.M. Rosiglitazone Produces a Greater Reduction in Circulating Platelet Activity Compared with Gliclazide in Patients with Type 2 Diabetes Mellitus--an Effect Probably Mediated by Direct Platelet PPARgamma Activation. Atherosclerosis 2008, 197, 718–724. https://doi.org/10.1016/j.atherosclerosis.2007.07.020.

127. Derosa, G.; Salvadeo, S.A.; D'Angelo, A.; Fogari, E.; Ragonesi, P.D.; Ciccarelli, L.; Piccinni, M.N.; Ferrari, I.;

Gravina, A.; Maffioli, P.; et al. Rosiglitazone Therapy Improves Insulin Resistance Parameters in Overweight and Obese Diabetic Patients Intolerant to Metformin. Arch. Med. Res. 2008, 39, 412–419. https://doi.org/10.1016/j.arcmed.2007.12.009.

128. Ko, G.T.; Tsang, P.C.; Wai, H.P.; Kan, E.C.; Chan, H.C. Rosiglitazone versus Bedtime Insulin in the Treatment of Patients with Conventional Oral Antidiabetic Drug Failure: A 1-Year Randomized Clinical Trial. Adv. Ther. 2006, 23, 799–808. https://doi.org/10.1007/BF02850321.

129. Satirapoj, B.; Watanakijthavonkul, K.; Supasyndh, O. Safety and Efficacy of Low Dose Pioglitazone Compared with Standard Dose Pioglitazone in Type 2 Diabetes with Chronic Kidney Disease: A Randomized Controlled Trial. PLoS ONE 2018, 13, e0206722. https://doi.org/10.1371/journal.pone.0206722.

130. Khaloo, P.; Komeleh, S.A.; Alemi, H.; Mansournia, M.A.; Mohammadi, A.; Yadegar, A.; Afarideh, M.; Esteghamati, S.; Nakhjavani, M.; Esteghamati, A. Sitagliptin vs. Pioglitazone as Add-on Treatments in Patients with Uncontrolled Type 2 Diabetes on the Maximal Dose of Metformin plus Sulfonylurea. J. Endocrinol. Investig. 2019, 42, 851–857. https://doi.org/10.1007/s40618-018-0991-0.

131. Tan, M.H.; Johns, D.; Strand, J.; Halse, J.; Madsbad, S.; Eriksson, J.W.; Clausen, J.; Konkoy, C.S.; Herz, M.; Group, G.S. Sustained Effects of Pioglitazone vs. Glibenclamide on Insulin Sensitivity, Glycaemic Control, and Lipid Profiles in Patients with Type 2 Diabetes. Diabet. Med. 2004, 21, 859–866. https://doi.org/10.1111/j.1464-5491.2004.01258.x.

132. Wilding, J.P.; Gause-Nilsson, I.; Persson, A.; Group, G.S. Tesaglitazar, as Add-on Therapy to Sulphonylurea, Dose-Dependently Improves Glucose and Lipid Abnormalities in Patients with Type 2 Diabetes. Diabetes Vasc. Dis. Res. 2007, 4, 194–203. https://doi.org/10.3132/dvdr.2007.040.

133. Esteghamati, A.; Azizi, R.; Ebadi, M.; Noshad, S.; Mousavizadeh, M.; Afarideh, M.; Nakhjavani, M. The Comparative Effect of Pioglitazone and Metformin on Serum Osteoprotegerin, Adiponectin and Intercellular Adhesion Molecule Concentrations in Patients with Newly Diagnosed Type 2 Diabetes: A Randomized Clinical Trial. Exp. Clin. Endocrinol. Diabetes 2015, 123, 289–295. https://doi.org/10.1055/s-0034-1396864.

134. Yale, J.F.; Valiquett, T.R.; Ghazzi, M.N.; Owens-Grillo, J.K.; Whitcomb, R.W.; Foyt, H.L. The Effect of a Thiazolidinedione Drug, Troglitazone, on Glycemia in Patients with Type 2 Diabetes Mellitus Poorly Controlled with Sulfonylurea and Metformin. A Multicenter, Randomized, Double-Blind, Placebo-Controlled Trial. Ann. Intern. Med. 2001, 134, 737–745. https://doi.org/10.7326/0003-4819-134-9_part_1-200105010-00010.

135. Gastaldelli, A.; Miyazaki, Y.; Mahankali, A.; Berria, R.; Pettiti, M.; Buzzigoli, E.; Ferrannini, E.; DeFronzo, R.A. The Effect of Pioglitazone on the Liver: Role of Adiponectin. Diabetes Care 2006, 29, 2275–2281. https://doi.org/10.2337/dc05-2445.

136. Jung, H.S.; Cho, Y.M.; Kim, K.W.; Youn, B.S.; Yu, K.Y.; Park, H.J.; Shin, C.S.; Kim, S.Y.; Lee, H.K.; Park, K.S. The Effects of Insulin Sensitizers on the Plasma Concentrations of Adipokines in Type 2 Diabetic Patients. Diabetes Metab. J. 2003, 27, 476–489.

137. Erdem, G.; Dogru, T.; Tasci, I.; Bozoglu, E.; Muhsiroglu, O.; Tapan, S.; Ercin, C.N.; Sonmez, A. The Effects of Pioglitazone and Metformin on Plasma Visfatin Levels in Patients with Treatment Naive Type 2 Diabetes Mellitus. Diabetes Res. Clin. Pract. 2008, 82, 214–218. https://doi.org/10.1016/j.diabres.2008.07.021.

138. Fidan, E.; Ersoz, H.O.; Yilmaz, M.; Yilmaz, H.; Kocak, M.; Karahan, C.; Erem, C. The Effects of Rosiglitazone and Metformin on Inflammation and Endothelial Dysfunction in Patients with Type 2 Diabetes Mellitus. Acta Diabetol.

2011, 48, 297–302. https://doi.org/10.1007/s00592-011-0276-y.

139. Jung, H.S.; Youn, B.-S.; Cho, Y.M.; Yu, K.-Y.; Park, H.J.; Shin, C.S.; Kim, S.Y.; Lee, H.K.; Park, K.S. The Effects of Rosiglitazone and Metformin on the Plasma Concentrations of Resistin in Patients with Type 2 Diabetes Mellitus. Metab. Clin. Exp. 2005, 54, 314–320. https://doi.org/10.1016/j.metabol.2004.05.019.

140. Gupta, M.; Teoh, H.; Kajil, M.; Tsigoulis, M.; Quan, A.; Braga, M.F.; Verma, S. The Effects of Rosiglitazone on Inflammatory Biomarkers and Adipokines in Diabetic, Hypertensive Patients. Exp. Clin. Cardiol. 2012, 17, 191–196.

141. Göke, B.; Gause-Nilsson, I.; Persson, A. The Effects of Tesaglitazar as Add-on Treatment to Metformin in Patients with Poorly Controlled Type 2 Diabetes. Diabetes Vasc. Dis. Res. 2007, 4, 204–213. https://doi.org/10.3132/dvdr.2007.041.

142. Pan, C.; Gao, Y.; Gao, X.; Li, G.; Luo, B.; Shi, H.; Tian, H.; Jia, P.; Lin, H.; Xing, X.; et al. The Efficacy and Safety of Pioglitazone Hydrochloride in Combination with Sulphonylureas and Metfomin in the Treatment of Type 2 Diabetes Mellitus a 12-Week Randomized Multi-Centres Placebo-Controlled Parallel Study. Zhonghua Nei Ke Za Zhi 2002, 41, 388–392.

143. Rosenblatt, S.; Miskin, B.; Glazer, N.B.; Prince, M.J.; Robertson, K.E. The Impact of Pioglitazone on Glycemic Control and Atherogenic Dyslipidemia in Patients with Type 2 Diabetes Mellitus: Coron. Artery Dis. 2001, 12, 413–423. https://doi.org/10.1097/00019501-200108000-00011.

144. Tan, K.C.B.; Chow, W.S.; Tso, A.W.K.; Xu, A.; Tse, H.F.; Hoo, R.L.C.; Betteridge, D.J.; Lam, K.S.L. Thiazolidinedione Increases Serum Soluble Receptor for Advanced Glycation End-Products in Type 2 Diabetes. Diabetologia 2007, 50, 1819–1825. https://doi.org/10.1007/s00125-007-0759-0.

145. Jovanovic, L.; Hassman, D.R.; Gooch, B.; Jain, R.; Greco, S.; Khutoryansky, N.; Hale, P.M. Treatment of Type 2 Diabetes with a Combination Regimen of Repaglinide plus Pioglitazone. Diabetes Res. Clin. Pract. 2004, 63, 127–134. https://doi.org/10.1016/j.diabres.2003.09.004.

146. Taslimi, S.; Esteghamati, A.; Rashidi, A.; Tavakkoli, H.M.; Nakhjavani, M.; Kebriaee-Zadeh, A. Treatment with Pioglitazone Is Associated with Decreased Preprandial Ghrelin Levels: A Randomized Clinical Trial. Peptides 2013, 40, 89–92. https://doi.org/10.1016/j.peptides.2012.12.020.

147. Kirk, J.K.; Pearce, K.A.; Michielutte, R.; Summerson, J.H. Troglitazone or Metformin in Combination with Sulfonylureas for Patients with Type 2 Diabetes? J. Fam. Pract. 1999, 48, 879–882.
